# Machine learning models for predicting extended length of stay and hospital charges in nontraumatic subarachnoid hemorrhage

**DOI:** 10.3389/fneur.2026.1737503

**Published:** 2026-02-04

**Authors:** Di Wu, Sihan Wang, Cong Wang, Yijia Xiang, Lingyu Hao, Zhen Wang, Xingye Zhai, Yi Wang

**Affiliations:** 1 Department of Neurosurgery, Tianjin Medical University General Hospital, Tianjin, China; 2 Key Laboratory of Post-Trauma Neuro-Repair and Regeneration in Central Nervous System, Ministry of Education and Key Laboratory of Injuries, Variations and Regeneration of Nervous System, Tianjin Neurological Institute, Tianjin, China; 3 Department of Infectious Diseases, Tianjin Medical University General Hospital, Tianjin, China; 4 Department of Neurosurgery, Qinghai University Affiliated Hospital, Xining, China

**Keywords:** nontraumatic subarachnoid hemorrhage, machine learning, length of stay, hospital charges, predictive modeling, SHAP, CatBoost, decision tree

## Abstract

**Background:**

Nontraumatic subarachnoid hemorrhage (SAH) is a critical condition requiring prolonged hospitalization and significant healthcare costs. Identifying factors contributing to extended length of stay (LOS) and predicting associated hospital charges can optimize clinical decision-making and resource allocation. This study aimed to construct and validate machine learning (ML) models to predict extended LOS and total charges in SAH using a national database.

**Methods:**

A retrospective cohort study was conducted using data from the National Inpatient Sample database, including 25,092 adult SAH patients. Twelve ML models were trained to predict extended LOS (defined as >17 days) based on clinical and demographic data. The variable screening process included univariate analysis, Spearman correlation analysis, least absolute shrinkage and selection operator (LASSO) regression, and Recursive Feature Elimination. SHapley Additive exPlanations (SHAP) values were used for model interpretation. Performance was assessed through receiver operating characteristic curves, precision-recall curves, calibration curves, and decision curve analysis (DCA). A decision tree model was also created to predict total hospital charges based on LOS. To identify factors contributing to high hospital charges in patients with extended LOS, univariate analysis, multivariate logistic regression, and LASSO regression were performed to select the most significant predictors.

**Results:**

Among the 12 ML models, the Categorical Boosting (CatBoost) model demonstrated the highest predictive performance, with an area under the receiver operating characteristic curve of 0.904 upon internal validation and 0.910 on hold-out validation. The model’s performance was optimal when 7 features were included, showing strong calibration and clinical applicability per DCA and SHAP. The decision tree model revealed a positive correlation between LOS and hospital charges. Additionally, key factors for predicting extended LOS and hospital charges included hydrocephalus, cerebral vasospasm, mechanical ventilation, and age. In patients with extended LOS, factors associated with high hospital charges were the total number of procedures, respiratory failure, tracheostomy, and hospital region.

**Conclusion:**

We constructed and validated ML models to predict extended LOS and hospital charges in SAH patients. The CatBoost model demonstrated strong predictive accuracy, while the decision tree model provided valuable insights into cost implications. Future multicenter studies are recommended to validate these models across diverse healthcare settings.

## Introduction

Nontraumatic subarachnoid hemorrhage (SAH) is a critical condition characterized by bleeding into the subarachnoid space surrounding the brain, caused primarily by aneurysmal rupture, arteriovenous malformations, and other cerebrovascular anomalies (1). SAH results in significant morbidity and mortality, with global annual incidence rates ranging from 6 to 10 per 100,000 people. The mortality rate is particularly high in the first 24 to 48 h following the hemorrhage, with approximately 30–40% of affected individuals dying within the first month, and up to 50% experiencing significant neurological impairment (2, 3). Prompt diagnosis and early intervention are essential to improve outcomes, yet the severity of the condition often necessitates prolonged hospitalization, leading to substantial morbidity, mortality, and healthcare costs (4).

While effective management strategies have been developed to treat patients with SAH, the length of stay (LOS) in the hospital for these patients is often extended due to various complications such as vasospasm, hydrocephalus, rebleeding, and hospital-acquired infections. These complications not only increase the demand for intensive monitoring and interventions but also significantly elevate healthcare costs (5, 6). Despite the growing body of research on SAH, the factors that contribute to extended LOS are still not fully understood. Previous studies have primarily focused on the impact of specific complications or clinical characteristics in isolation, often overlooking the complex interplay of multiple factors that contribute to prolonged hospitalization. Moreover, traditional statistical models used in these studies often fail to account for nonlinear relationships and interactions among a large number of variables, thus limiting the accuracy and utility of these models in clinical practice (7–9).

Recent advancements in machine learning (ML) have the potential to overcome these limitations. Unlike traditional statistical methods, ML algorithms can process large, complex datasets and identify patterns and relationships that are often too intricate for conventional models to detect (10, 11). By integrating diverse types of data, including patient demographics, clinical characteristics, and treatment details, ML models can provide more accurate predictions of outcomes such as LOS (12, 13). Various ML techniques, such as Random Forest (RF), Support Vector Machine (SVM), and Gradient Boosting Machine (GBM), have been successfully applied in predicting hospital LOS in various medical contexts, including stroke and trauma (14, 15). However, one of the major challenges in applying ML to healthcare data is ensuring the interpretability of the models. While ML can offer highly accurate predictions, it is essential that these models be transparent and understandable to clinicians. Techniques like SHapley Additive exPlanations (SHAP) allow for the explanation of how each feature contributes to the model’s predictions, thus enhancing the clinical applicability and trustworthiness of the model (12, 16).

This study aims to fill these gaps by constructing and validating ML models to predict the risk of prolonged hospitalization in SAH patients using data from the National Inpatient Sample (NIS) database, one of the largest publicly available inpatient healthcare databases (17, 18). In addition to predicting extended LOS, our study will explore the relationship between LOS and hospital charges, providing insights into how extended stays impact healthcare expenditures. By identifying key predictors and leveraging advanced ML techniques, this research aims to create accurate, interpretable models that can be integrated into clinical workflows, helping healthcare providers optimize resource allocation and improve patient outcomes. Furthermore, the findings will offer valuable guidance for cost-management strategies by analyzing factors contributing to both extended LOS and higher hospital charges.

## Materials and methods

The Materials and methods section of this study outlines the data source, patient population, and detailed methodology used for the derivation and validation of predictive models. The study utilized data from the NIS database, focusing on adult patients with SAH between 2016 and 2020. This section provides an overview of the variables considered, including demographic information, clinical conditions, and hospital characteristics, as well as the process of cohort division, data collection, imputation, and analysis. The methodology follows a multi-step process for variable screening, model construction, and evaluation, using various machine learning algorithms. The methodological workflow includes the following key steps, each detailed in Supplementary material 1: (1) Data source and patient population, (2) Data collection, processing, and cohort division, (3) Initial variable screening, model construction, and model comparison for predicting extended LOS, (4) Feature selection, model validation and model explanation for predicting extended LOS, (5) Web-based tool for predicting extended LOS using a streamlit framework, (6) Relationship between LOS and total charges and construction of a model for predicting total charges based on the LOS, and (7) Factors associated with high hospital charges in patients with extended LOS. The statistical software and significance thresholds used are also fully documented in Supplementary material 1.

## Results

### Patient characteristics

**Figure 1:**
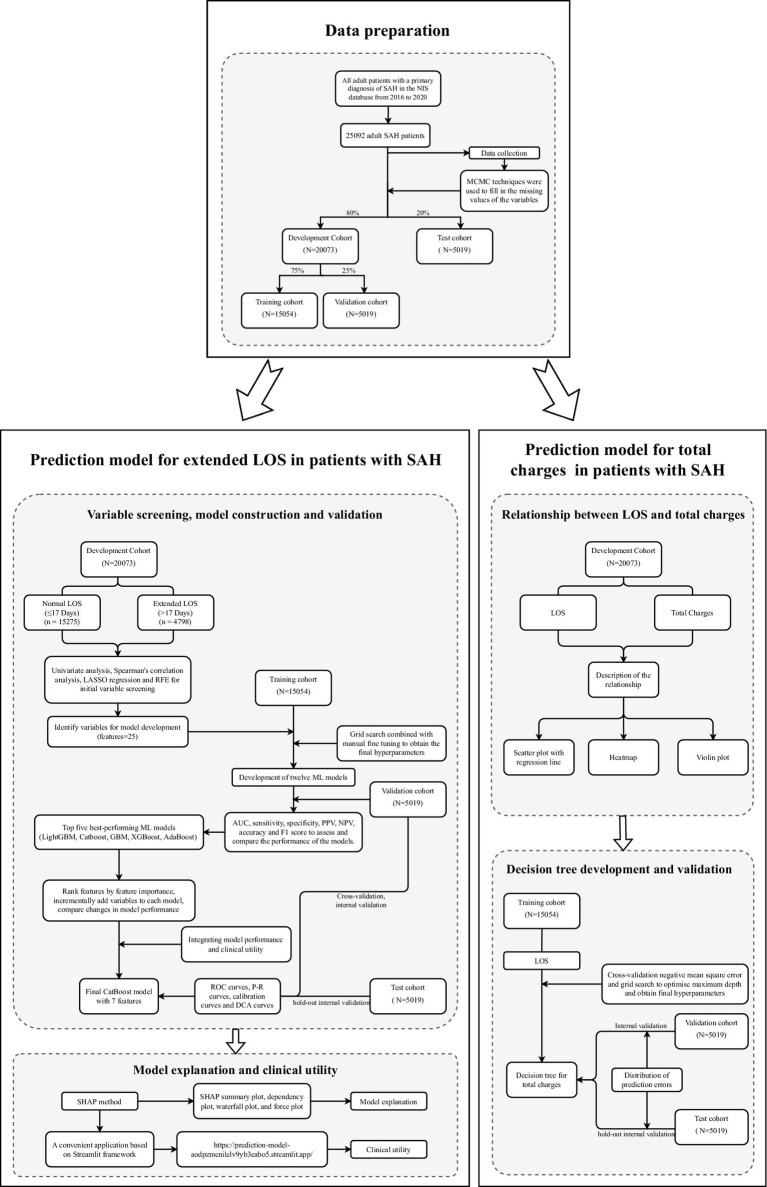
Flow chart of the study design. The figure illustrates the detailed study design and methodology, outlining the sequential steps taken from data collection to the final analysis. AUC, area under the receiver operating characteristic curve; AdaBoost, adaptive boosting; CatBoost, categorical boosting; DCA, decision curve analysis; GBM, gradient boosting machine; LASSO, least absolute shrinkage and selection operator; LightGBM, light gradient boosting machine; LOS, length of stay; McMC, Markov chain Monte Carlo; ML, machine learning; NIS, national inpatient sample; NPV, negative predictive value; P–R, precision–recall; PPV, positive predictive value; ROC, receiver operating characteristic; SAH, nontraumatic subarachnoid hemorrhage; XGBoost, EXtreme Gradient Boosting.

We included a total of 25,092 adult SAH patients from the NIS database, encompassing 90 variables. The actual and missing patient counts for each variable are detailed in Supplementary Table 2. After imputing missing values through Markov chain Monte Carlo techniques, we randomly split the data into an 80% development cohort (*n* = 20,073) and a 20% test cohort (*n* = 5,019). Within the development cohort, we further divided the data into a 75% training cohort (*n* = 15,054) and a 25% validation cohort (*n* = 5,019). The patient characteristics of each cohort are detailed in Supplementary Table 3. The *p* values (P^#^ and P*) in the table are nearly all greater than 0.05, indicating that there were no significant differences between the cohorts and confirming the success of the random allocation. On the basis of the interquartile range of LOS, which was 9.0 (3.0, 17.0) days, we divided the development cohort patients into a normal LOS group (≤17 days, *n* = 15,275) and an extended LOS group (>17 days, *n* = 4,798), as shown in Table 1. The detailed study design and methodology are illustrated in Figure 1.

**Table 1:** Comparison of patient characteristics between normal and extended LOS groups in the development cohort.

Variables (%)	Normal LOS (≤17 Days) (*n* = 15,275)	Extended LOS (>17 Days) (*n* = 4,798)	*P*
Patient demographics			
Age (years)			<0.001
Mean ± SD	60.3 ± 16.7	56.8 ± 14.2	
Median (IQR)	61.0 (50.0, 72.0)	58.0 (48.0, 67.0)	
Gender (%)			<0.001
Females	9,098 (59.6)	3,131 (65.3)	
Males	6,177 (40.4)	1,677 (34.7)	
Race (%)			<0.001
White	9,467 (62.0)	2,620 (54.6)	
Black	2,312 (15.1)	930 (19.4)	
Hispanic	2026 (13.3)	704 (14.7)	
Other	1,470 (9.6)	544 (11.3)	
Length of stay (days)			<0.001
Mean ± SD	6.9 ± 5.1	29.1 ± 18.0	
Median (IQR)	6.0 (2.0, 11.0)	24.0 (20.0, 31.0)	
Total charges (dollars)			<0.001
Mean ± SD	166631.5 ± 171441.4	597331.5 ± 412984.8	
Median (IQR)	114999.0 (43386.0, 229051.0)	492071.5 (327682.5, 739801.3)	
Median household income quartile (%)			0.001
0–25th	4,444 (29.1)	1,515 (31.6)	
26–50th	3,886 (25.4)	1,226 (25.6)	
51–75th	3,647 (23.9)	1,121 (23.4)	
76–100th	3,298 (21.6)	936 (19.5)	
Primary expected payer (%)			<0.001
Medicare	6,245 (40.9)	1,474 (30.7)	
Medicaid	2,194 (14.4)	1,089 (22.7)	
Private insurance	5,221 (34.2)	1763 (36.7)	
Other	1,615 (10.6)	472 (9.8)	
Non-elective admission	14,579 (95.4)	4,641 (96.7)	<0.001
Hospitalization year (%)			0.863
2016	3,048 (20.0)	949 (19.8)	
2017	3,109 (20.4)	980 (24.0)	
2018	3,089 (20.2)	962 (20.1)	
2019	3,123 (20.4)	1,014 (21.1)	
2020	2,906 (19.0)	893 (18.6)	
Hospitalization season (%)			0.426
Spring (March–May)	3,781 (24.8)	1,150 (24.0)	
Summer (June–August)	3,662 (24.0)	1,167 (24.3)	
Fall (September–November)	3,834 (25.1)	1,250 (26.1)	
Winter (December–February)	3,998 (26.2)	1,231 (25.7)	
Hospitalization on weekends (%)	3,990 (26.1)	1,350 (28.1)	0.005
Hospital admission transfer indicator (%)			<0.001
Not transferred/Standard admission	8,971 (58.7)	2,335 (48.7)	
From acute care hospital	5,608 (36.7)	2,268 (47.3)	
From other facility	696 (4.6)	195 (4.1)	
Hospital discharge transfer indicator (%)			<0.001
Not transferred	11,454 (75.0)	1,584 (33.0)	
To acute care hospital	902 (5.9)	117 (2.4)	
To other facility	2,919 (19.1)	3,097 (64.5)	
Died during hospitalization	3,506 (23.0)	242 (5.0)	<0.001
Hospital demographics (%)			
Hospital region			0.004
Northeast	2,530 (16.6)	963 (20.1)	
Midwest	3,095 (20.3)	875 (18.2)	
South	6,019 (39.4)	1849 (38.5)	
West	3,631 (23.8)	1,111 (23.2)	
Hospital bed size			<0.001
Small	1,260 (8.2)	225 (4.7)	
Medium	3,351 (21.9)	837 (17.4)	
Large	10,664 (69.8)	3,736 (77.9)	
Hospital location/teaching status			<0.001
Rural	316 (2.1)	21 (0.4)	
Urban nonteaching	1,555 (10.2)	279 (5.8)	
Urban teaching	13,404 (87.8)	4,498 (93.7)	
Hospital control/ownership (%)			<0.001
Government, nonfederal	2082 (13.6)	772 (16.1)	
Private, not-profit	11,453 (75.0)	3,521 (73.4)	
Private, invest-own	1740 (11.4)	505 (10.5)	
Diagnosis, symptoms and complications on admission and during hospitalization (%)			
Hypertension	10,650 (69.7)	3,435 (71.6)	0.013
Type II diabetes	2,726 (17.8)	782 (16.3)	0.014
Coronary heart disease	2,367 (15.5)	712 (14.8)	0.271
Atrial fibrillation	1,630 (10.7)	459 (9.6)	0.028
Hyperlipidemia	5,061 (33.1)	1,225 (25.5)	<0.001
Elevated blood glucose level	1,229 (8.0)	575 (12.0)	<0.001
Chronic obstructive pulmonary disease	1,185 (7.8)	387 (24.6)	0.488
Hypothyroidism	1,526 (10.0)	344 (7.2)	<0.001
Anxiety	1,442 (9.4)	459 (9.6)	0.794
Depression	1,439 (9.4)	455 (9.5)	0.897
Overweight and obesity	1780 (11.7)	691 (14.4)	<0.001
Tobacco use	5,546 (36.3)	1,651 (34.4)	0.017
Alcohol abuse	719 (4.7)	354 (7.4)	<0.001
History of transient ischemic attack and cerebral infarction	1,168 (7.6)	163 (3.4)	<0.001
Long term (current) use of anticoagulants and antithrombotic/antiplatelets	1,398 (9.2)	201 (4.2)	<0.001
Long term (current) use of aspirin	1872 (12.3)	419 (8.7)	<0.001
Contact with and (suspected) exposure to communicable diseases	757 (5.0)	246 (5.1)	0.635
Kidney failure	160 (1.0)	32 (0.7)	0.018
Hepatic failure	2,403 (15.7)	860 (17.9)	<0.001
Paralytic	192 (1.3)	66 (1.4)	0.525
Disorders of fluid, electrolyte and acid–base balance	5,926 (38.8)	3,506 (73.1)	<0.001
Shock	623 (4.1)	238 (5.0)	0.009
Respiratory failure	4,301 (28.2)	2,984 (62.2)	<0.001
Convulsions	971 (6.4)	451 (9.4)	<0.001
Muscle spasm	81 (0.5)	28 (0.6)	0.661
Pulmonary infection	699 (4.6)	1,243 (25.9)	<0.001
Urinary tract infection	1,172 (7.7)	1,201 (25.0)	<0.001
Intracranial infection	142 (0.9)	312 (6.5)	<0.001
Sepsis	448 (2.9)	569 (11.9)	<0.001
Cerebral edema	3,549 (23.2)	1992 (41.5)	<0.001
Hydrocephalus	3,897 (25.5)	3,394 (70.7)	<0.001
Nausea and vomiting	436 (2.9)	96 (2.0)	0.001
Headache	4,219 (27.6)	3,424 (71.4)	<0.001
Anemia	2,605 (17.1)	1803 (37.6)	<0.001
Gastro-esophageal reflux	310 (2.0)	1,306 (27.2)	<0.001
Dysphagia	916 (6.0)	1,359 (28.3)	<0.001
Aphasia	1,023 (6.7)	687 (14.3)	<0.001
Nontraumatic intracerebral hemorrhage	3,356 (22.0)	1,531 (31.9)	<0.001
Elevated white blood cell count	1,453 (9.5)	552 (11.5)	<0.001
Thrombocytopenia	580 (3.8)	246 (5.1)	<0.001
Facial weakness	804 (5.3)	329 (6.9)	<0.001
Embolism and thrombosis of deep veins of lower extremity	195 (1.3)	367 (7.6)	<0.001
Cerebral aneurysm, no ruptured	1,269 (8.3)	636 (13.3)	<0.001
Cerebrovascular arteriovenous malformation	158 (1.0)	83 (1.7)	<0.001
Disordered phosphorus metabolism	1,601 (10.5)	1,023 (21.3)	<0.001
Disordered magnesium metabolism	723 (4.7)	489 (10.2)	<0.001
Cerebral vasospasm and vasoconstriction	1799 (11.8)	2075 (43.2)	<0.001
Constipation	912 (6.0)	386 (8.0)	<0.001
Total number of diagnoses			<0.001
Mean ± SD	13.9 ± 6.6	20.0 ± 6.5	
Median (IQR)	13.0 (9.0, 18.0)	20.0 (16.0, 25.0)	
Procedures during hospitalization (%)			
Occlusion of intracranial artery	1795 (11.8)	1,346 (28.1)	<0.001
Restriction of intracranial artery	2,410 (15.8)	1974 (41.1)	<0.001
Excision of intracranial artery	78 (0.5)	69 (1.4)	<0.001
Bypass operation of intracranial arteries	10 (0.1)	22 (0.5)	<0.001
Monitoring of arterial pulse	521 (3.4)	294 (6.1)	<0.001
Monitoring of arterial pressure	909 (6.0)	560 (11.7)	<0.001
Monitoring of central nervous electrical activity	477 (3.1)	520 (10.8)	<0.001
Percutaneous ventriculostomy	2,285 (15.0)	2,584 (53.9)	<0.001
Airway intubation	2,381 (15.6)	1,638 (34.1)	<0.001
Tracheostomy	54 (0.4)	538 (11.2)	<0.001
Mechanical ventilation			
Less than 24 consecutive hours	1,489 (9.7)	252 (5.3)	<0.001
24–96 consecutive hours	1789 (11.7)	795 (16.6)	<0.001
Greater than 96 consecutive hours	1,065 (7.0)	1847 (38.5)	<0.001
Lumbar puncture	631 (4.1)	325 (6.8)	<0.001
Insertion of feeding device into stomach	310 (2.0)	1,306 (27.2)	<0.001
Introduction of nutritional substance into upper GI	231 (1.5)	499 (10.4)	<0.001
Insertion of monitoring device into upper artery	1,126 (7.4)	778 (16.2)	<0.001
Insertion of infusion device into superior vena cava	2,103 (13.8)	1797 (37.5)	<0.001
Ultrasonography of superior vena cava	376 (2.5)	345 (7.2)	<0.001
Fluoroscopy of artery	6,127 (40.1)	2,745 (57.2)	<0.001
Administration of thrombolytics and platelet inhibitors	194 (1.3)	161 (3.4)	<0.001
Transfusion of blood and blood products	628 (4.1)	444 (9.3)	<0.001
Total number of procedures			<0.001
Mean ± SD	4.0 ± 3.8	10.5 ± 6.1	
Median (IQR)	3.0 (1.0, 6.0)	10.0 (6.0, 14.0)	

Continuous variables were presented as means [standard deviation (SD)] or medians [interquartile range (IQR)]. Categorical variables were presented as numbers (percentage).

GI, gastrointestinal; LOS, length of stay.

### Initial variable screening for predicting extended LOS

Table 1 presents the univariate analysis of 90 factors between the Normal LOS group and the Extended LOS group in the development cohort. The analysis revealed no statistically significant differences (*p* > 0.05) in factors such as hospitalization year, hospitalization season, coronary heart disease, chronic obstructive pulmonary disease, anxiety, depression, contact with and (suspected) exposure to communicable diseases, paralytic conditions, or muscle spasm. Following the univariate analysis, we performed a Spearman correlation analysis on 81 factors. When two features were highly correlated (correlation coefficient > 0.6), we excluded the feature with the lower correlation to the outcome from the dataset. As illustrated in Supplementary Figure 1, the excluded variables included length of stay, total charges, headache, cerebral aneurysm (not ruptured), monitoring of arterial pulse, insertion of feeding device into the stomach, and total number of procedures. Subsequently, 74 factors were included in the least absolute shrinkage and selection operator (LASSO) regression analysis. Supplementary Figures 2A–C shows that, after LASSO regression and 5-fold cross-validation, the number of variables decreased to 38. These variables were then included in the recursive feature elimination (RFE) process. Supplementary Figures 2D,E shows that the final 25 variables selected after RFE were hospital discharge transfer indicator, hydrocephalus, total number of diagnoses, percutaneous ventriculostomy, cerebral vasospasm and vasoconstriction, mechanical ventilation (greater than 96 consecutive hours), gastroesophageal reflux, died during hospitalization, age, respiratory failure, pulmonary infection, dysphagia, restriction of intracranial artery, disorders of fluid, electrolyte, and acidbase balance, insertion of infusion device into superior vena cava, primary expected payer, tracheostomy, urinary tract infection, airway intubation, anemia, occlusion of intracranial artery, introduction of nutritional substance into upper GI, embolism and thrombosis of deep veins of the lower extremity, sepsis, and intracranial infection.

### Model construction and performance comparison for predicting extended LOS

**Figure 2:**
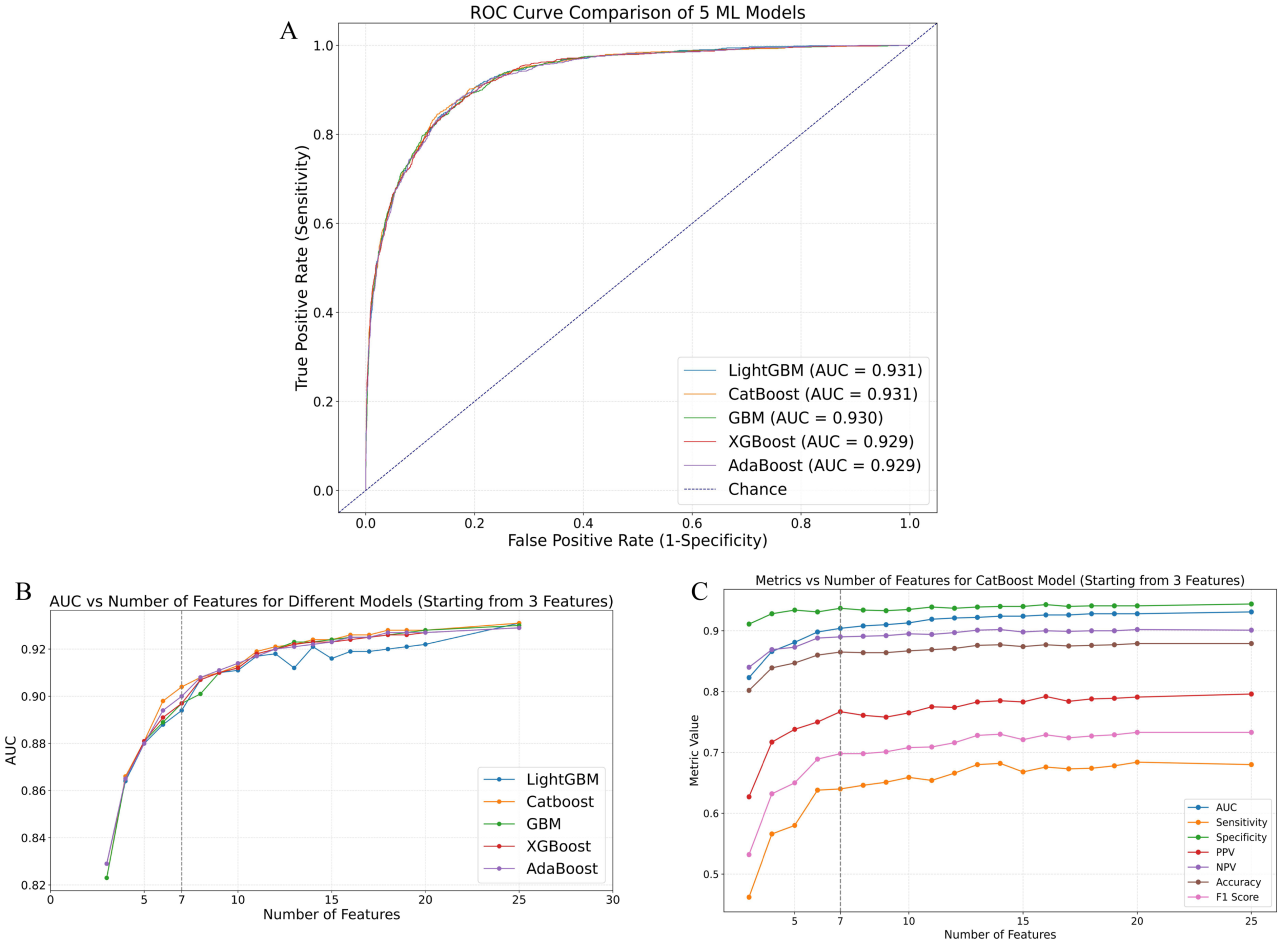
Performance comparison of ML models in predicting extended LOS using the validation cohort. **(A)** ROC curves of the top five best-performing ML models: LightGBM, CatBoost, GBM, XGBoost, and AdaBoost. **(B)** AUC values of these models as the number of features increases, demonstrating the stability and improvement of model performance with additional features. **(C)** Detailed performance metrics of the CatBoost model, including AUC, sensitivity, specificity, PPV, NPV, accuracy, and F1 score, as the number of features increases, highlighting the optimal performance with 7 features. AdaBoost, adaptive boosting; AUC, area under the receiver operating characteristic curve; CatBoost, categorical boosting; GBM, gradient boosting machine; LightGBM, light gradient boosting machine; ML: machine learning; NPV, negative predictive value; PPV, positive predictive value; ROC, receiver operating characteristic; XGBoost, eXtreme Gradient Boosting.

The initially selected 25 variables were incorporated into 12 different ML models to predict the occurrence of extended LOS. The performance metrics of these 12 models are listed in Supplementary Table 4, and their corresponding confusion matrices are presented in Supplementary Figure 3. The top five ML models with the best performance were Light Gradient Boosting Machine (LightGBM), Categorical Boosting (CatBoost), GBM, eXtreme Gradient Boosting (XGBoost), and Adaptive Boosting (AdaBoost), with AUCs of 0.931, 0.931, 0.930, 0.929, and 0.929, respectively. The receiver operating characteristic (ROC) curves for these top five models are shown in Figure 2A. The variable importance plots for beeswarm and the plots for the top 20 features of the top five ML models are depicted in Supplementary Figures 4A–E, 5A–E, respectively.

The area under the receiver operating characteristic curve (AUC) values for the top five performing ML models as the number of included features increased are presented in Supplementary Table 5. Additionally, Figure 2B illustrates the AUC trends of these models as the number of variables increased. As shown in these figures and tables, except for LightGBM, which showed some fluctuations in AUC values as the number of features increased, the other four models (CatBoost, GBM, XGBoost, and AdaBoost) exhibited consistent improvements in AUC with an increasing number of features. Notably, these four models showed minimal AUC differences when the number of included features ranged from 1–5 or 8–20 or equaled 25. However, the CatBoost model displayed superior predictive performance compared with the other models, especially when 6 or 7 features were included. Figure 2C and Supplementary Table 6 present the performance of the CatBoost model with varying numbers of features. The sensitivity, specificity, positive predictive values (PPV), negative predictive values (NPV), accuracy, and F1 score were calculated at the optimal cutoff value, which maximized the Youden index. This further confirmed the excellent performance of the CatBoost model when seven features were included, ensuring its robustness and clinical applicability.

### Identification and validation of the final model for predicting extended LOS

The final model was determined by comprehensively considering the changes in AUC values as the number of included features increased while maintaining clinical applicability through controlled feature selection. As shown in Figures 2B,C, the CatBoost model with 7 features met our initial model selection criteria, not only demonstrating excellent predictive performance but also maintaining clinical applicability through a manageable number of features. Supplementary Figures 6A–D presents the CatBoost model’s ROC curves, precision–recall (P-R) curves, decision curve analysis (DCA), and calibration curves with the top 3, 6, 7, 8, and 25 features included. Although the CatBoost model with 25 features achieved the highest AUC and P-R AUC values (0.931 and 0.832, respectively), the model with 7 features still exhibited high predictive performance (AUC = 0.904, P-R AUC = 0.781). Within a broad threshold probability range (0.05–0.95), the 7-feature model demonstrated substantial clinical net benefit and good calibration (HL *p* value = 0.302).

**Figure 3:**
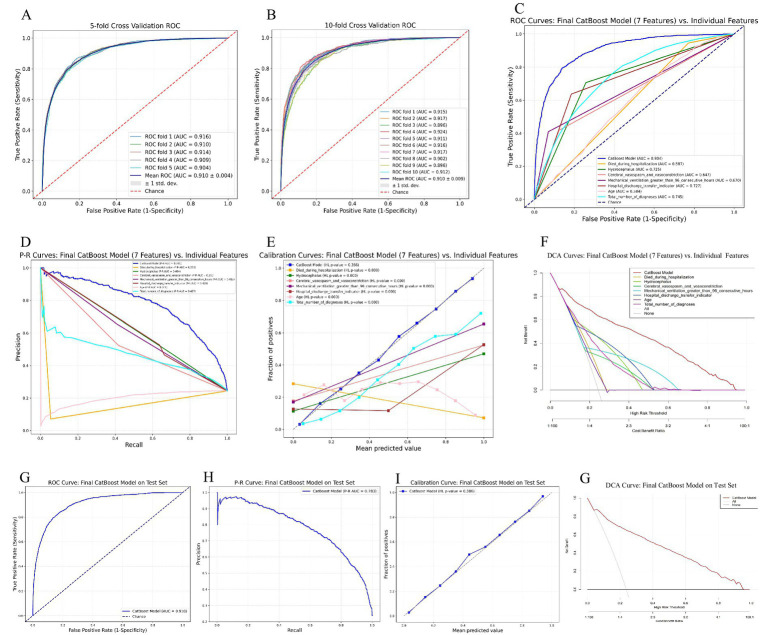
Performance evaluation and validation of the 7-feature CatBoost model for predicting extended LOS. **(A,B)** Five-fold and ten-fold cross-validation of the 7-feature CatBoost model using the validation cohort, showing robust stability with mean AUCs of 0.910 ± 0.004 and 0.910 ± 0.009, respectively. **(C,D)** ROC and P-R curves comparing the predictive performance of each individual feature in the 7-feature model against the full 7-feature CatBoost model within the validation cohort, demonstrating the superior predictive ability of the combined model. **(E,F)** Calibration and decision curves comparing the 7-feature CatBoost model with individual features within the validation cohort, highlighting the model’s superior calibration and higher clinical utility. **(G–J)** External validation of the 7-feature CatBoost model using the test cohort, including ROC curve **(G)**, P-R curve **(H)**, calibration curve **(I)**, and DCA **(J)**, demonstrating that the final model maintained good accuracy and high clinical utility in the external cohort. AUC, area under the receiver operating characteristic curve; CatBoost, categorical boosting; DCA, decision curve analysis; LOS, length of stay; P-R, precision–recall; ROC, receiver operating characteristic.

To effectively estimate the generalization ability (test error) of the 7-feature CatBoost model determined by using the training cohort, we performed cross-validation with the validation cohort. As shown in Figures 3A,B, the final model demonstrated robust stability in five-fold and ten-fold cross-validation, with mean AUC values of 0.910 ± 0.004 and 0.910 ± 0.009, respectively.

We subsequently compared the predictive performance of each variable in the model with that of the 7-feature CatBoost model. As illustrated in Figures 3C,D, the predictive abilities of individual variables such as Died during hospitalization (ΔAUC = 0.317, *p* < 0.01; ΔP-R AUC = 0.546), Hydrocephalus (ΔAUC = 0.179, *p* < 0.01; ΔP-R AUC = 0.377), Cerebral vasospasm and vasoconstriction (ΔAUC = 0.257, *p* < 0.01; ΔP-R AUC = 0.420), Mechanical ventilation greater than 96 consecutive hours (ΔAUC = 0.234, *p* < 0.01; ΔP-R AUC = 0.369), Hospital discharge transfer indicator (ΔAUC = 0.177, *p* < 0.01; ΔP-R AUC = 0.351), Age (ΔAUC = 0.320, *p* < 0.01; ΔP-R AUC = 0.580), and Total number of diagnoses (ΔAUC = 0.159, *p* < 0.01; ΔP-R AUC = 0.324) were all inferior to the final model in internal validation. The calibration curves indicated that the final model had superior calibration compared with that of the individual variables. Moreover, the DCA showed that the final model had greater clinical application value than the individual variables did.

Finally, to assess the practical generalizability and applicability of the final model, we performed hold-out internal validation using the test cohort, which was not included in the model construction. As shown in Figures 3G,H, the final model achieved AUC and P-R AUC values of 0.910 and 0.793, respectively, in the hold-out internal validation, which were similar to or slightly greater than the internal validation results (ΔAUC = −0.006, *p* = 0.175; ΔP-R AUC = −0.002). These results indicated that the final model performed well in both the internal and the hold-out internal validation. Figures 3I,J show that the calibration and decision curves for the test cohort were consistent with those for the validation cohort, which indicated that the final model maintained good accuracy and high clinical utility in the external cohort.

In addition to the performance metrics reported above, we also compared the predictive performance of the model that utilized a comprehensive set of 7 variables selected from the entire hospitalization period with the model based on 25 early hospitalization-related variables. The comparison of these two models’ results, including key performance metrics such as AUC, sensitivity, specificity, PPV, NPV, accuracy, and F1 score, is summarized in Supplementary Table 7. The results show that the model using all hospitalization-related variables (7 variables) performed significantly better, highlighting the importance of these variables for predicting extended LOS.

### Explanation of the final model for predicting extended LOS

Since clinicians often require prediction models to be directly explainable and interpretable, we utilized the SHAP method to interpret the output of the final model by calculating the contribution of each variable to the prediction. This approach provides two types of explanations: global explanations at the feature level and local explanations at the individual level.

**Figure 4:**
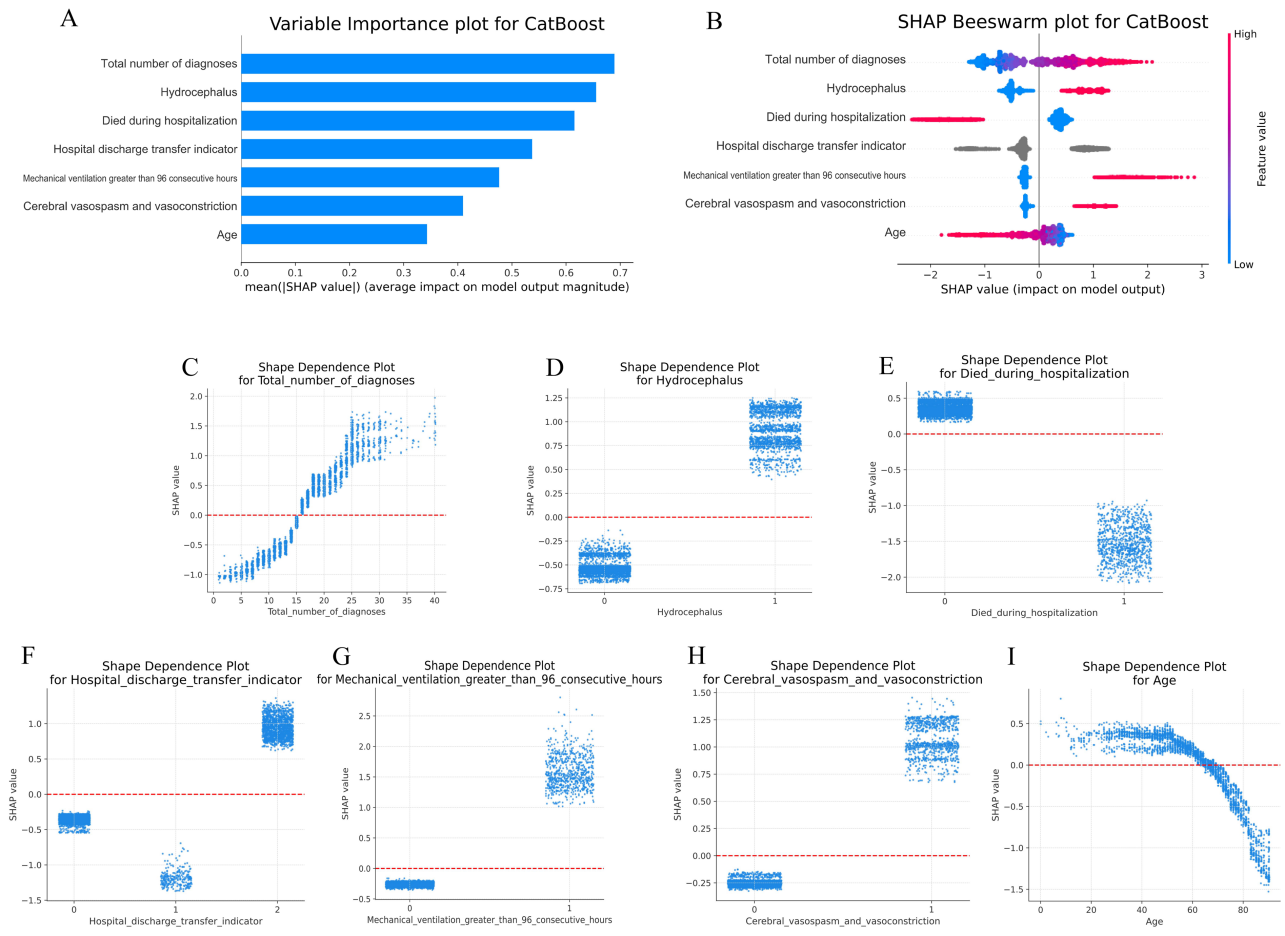
Global SHAP analysis of the 7-feature CatBoost model for predicting extended LOS. **(A)** Variable importance plot: This plot ranks the top 7 features in the CatBoost model based on their average SHAP values, providing an overall indication of each feature’s contribution to the prediction of extended LOS. Features are displayed in descending order of their importance, offering a global perspective on the model’s decision-making process. **(B)** SHAP beeswarm plot: Each dot in this plot represents an individual patient’s SHAP value for a specific feature. The color gradient (red for higher values, blue for lower values) indicates the actual value of the feature, with dots stacked vertically to show the density of the feature’s impact on the model’s predictions. This plot visually demonstrates how each feature influences the model’s predictions across the patient population. **(C–I)** SHAP dependence plot: These plots illustrate how the actual values of the top 7 features affect the model’s output. The x-axis represents the actual feature values, while the y-axis shows the corresponding SHAP values. A SHAP value above zero indicates a greater likelihood of predicting extended LOS, providing insight into the model’s behavior for specific features. CatBoost, categorical boosting; LOS, length of stay; SHAP: SHapley Additive exPlanations.

Global explanations describe the overall functionality of the model. As shown in the SHAP summary plots (the variable importance plots in Figure 4A and the beeswarm plots in Figure 4B), the contributions of each feature to the model were evaluated via average SHAP values and are displayed in descending order. Additionally, SHAP dependence plots were used to understand how a single feature affected the output of the prediction model. Figures 4C–I illustrates the relationship between the actual values and SHAP values for the 7 features, where SHAP values above zero corresponded to positive class predictions in the model, indicating a greater likelihood of extended LOS. For example, the presence of Hydrocephalus or Cerebral vasospasm and vasoconstriction during hospitalization pushed the decision toward the “Extended LOS” class. Conversely, when the Total number of diagnoses was ≤15, the decision was pushed toward the “normal LOS” class.

**Figure 5:**
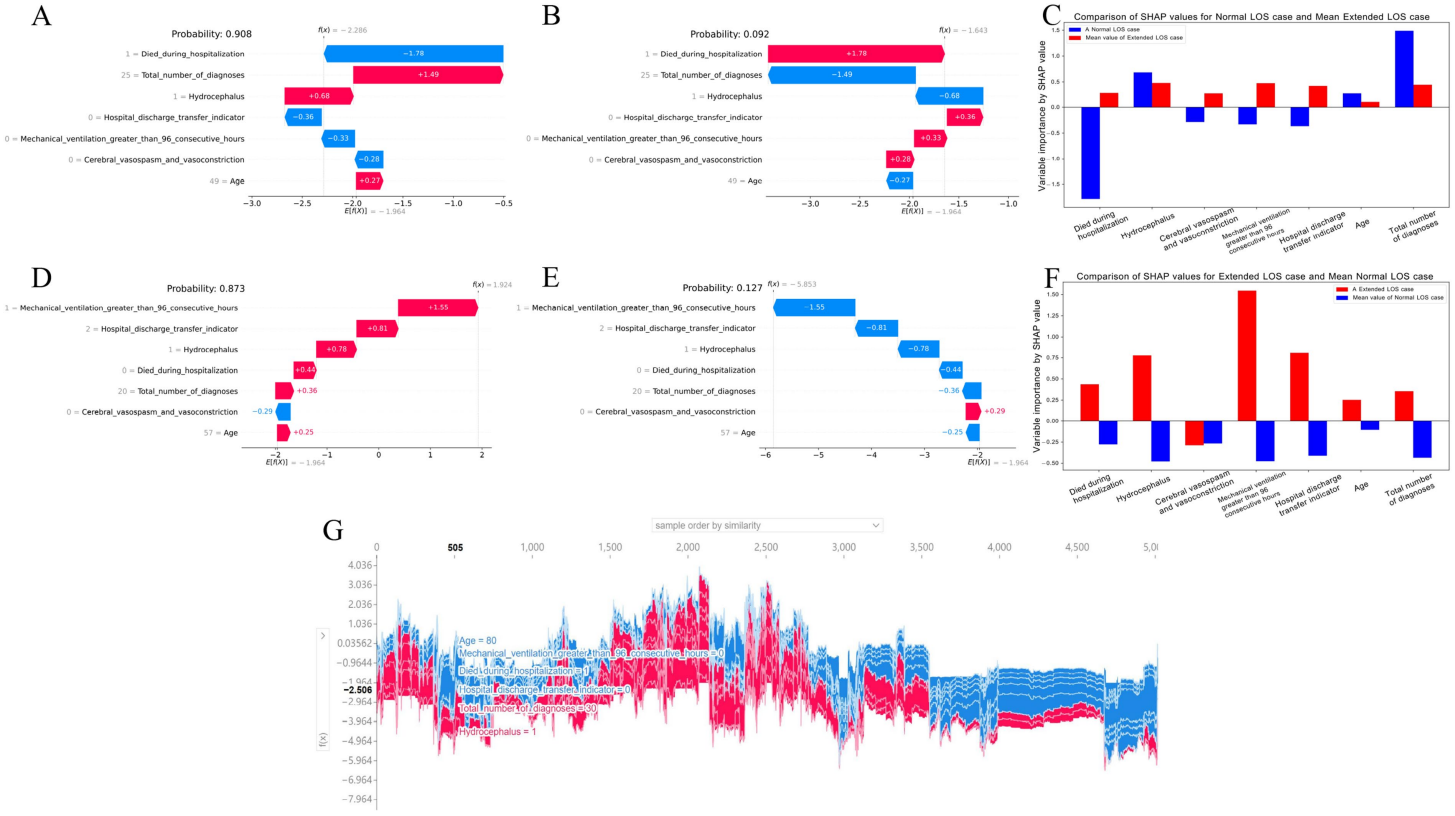
Local SHAP analysis of the 7-feature CatBoost model for predicting extended LOS **(A–C)** Local explanation for a patient with normal LOS: **(A)** Waterfall plot for normal LOS prediction: This plot illustrates the contributions of each feature to a patient’s predicted normal LOS. Positive SHAP values push the prediction toward normal LOS, while negative values push it toward extended LOS, showing the balance of factors influencing this outcome. **(B)** Waterfall plot for extended LOS prediction: This plot presents the contributions of each feature for the same patient, but in the context of a potential extended LOS, providing a comparative view against the normal LOS scenario. **(C)** Comparison of standardized SHAP values: This plot compares the SHAP values of the features for this patient with normal LOS against the average SHAP values for patients with extended LOS, highlighting the differences in feature impact. **(D–F)** Local explanation for a patient with extended LOS: **(D)** Waterfall plot for extended LOS prediction: This plot shows the SHAP values for a patient with a predicted extended LOS, indicating how each feature contributes to this outcome. **(E)** Waterfall plot for normal LOS prediction: Displays the feature contributions in the context of a potential normal LOS for the same patient, contrasting it with the extended LOS prediction. **(F)** Comparison of standardized SHAP values: Compares the SHAP values for this patient with extended LOS against the average SHAP values for patients with normal LOS, offering insight into the distinctive feature contributions. **(G)** Force plot for the internal validation cohort: This plot visualizes feature contributions for all patients in the internal validation cohort. The x-axis represents individual patients, and the y-axis shows the magnitude of feature contributions. A larger red area indicates a stronger push toward extended LOS. This force plot is available for interactive exploration at https://walkerdii.github.io/shap-force-plot/. CatBoost, categorical boosting; LOS, length of stay; SHAP, SHapley Additive exPlanations.

Local explanations analyze how a specific prediction was made for an individual by incorporating individualized input data. Figures 5A–C show a patient with a normal LOS. According to the prediction model, Figure 5A indicates a 90.8% probability toward the “Normal LOS” class, and Figure 5B indicates a 9.2% probability toward the “Extended LOS” class. The actual measured values of the features are displayed in the waterfall plots in Figures 5A,B. The values for died during hospitalization, hospital discharge transfer indicator, Mechanical ventilation greater than 96 consecutive hours, and Cerebral vasospasm and vasoconstriction pushed the decision toward the “normal LOS” class, whereas Total number of diagnoses, Hydrocephalus, and Age pushed it toward “Extended LOS.” Figure 5C compares the standardized SHAP values of each feature for this normal LOS patient with the average SHAP values for all patients with extended LOS.

Figures 5D–F illustrate a case with an extended LOS. Figures 5D,E display the features and their actual measured values that pushed or pulled the decision toward the “Extended LOS” class. The decision for this case leaned toward “Extended LOS” with an 87.3% probability and “Normal LOS” with a 12.7% probability. Figure 5F compares the standardized SHAP values of each feature for this extended LOS patient with the average SHAP values for all patients with a normal LOS.

Additionally, Figure 5G shows a force plot of interpretation for patients in the internal validation cohort. The x-axis represents each patient, and the y-axis represents the contributions of the features. An increased red area for an individual patient indicates a greater probability toward the decision of “Extended LOS.”

### Convenient application for clinical utility of the model in predicting extended LOS

**Figure 6:**
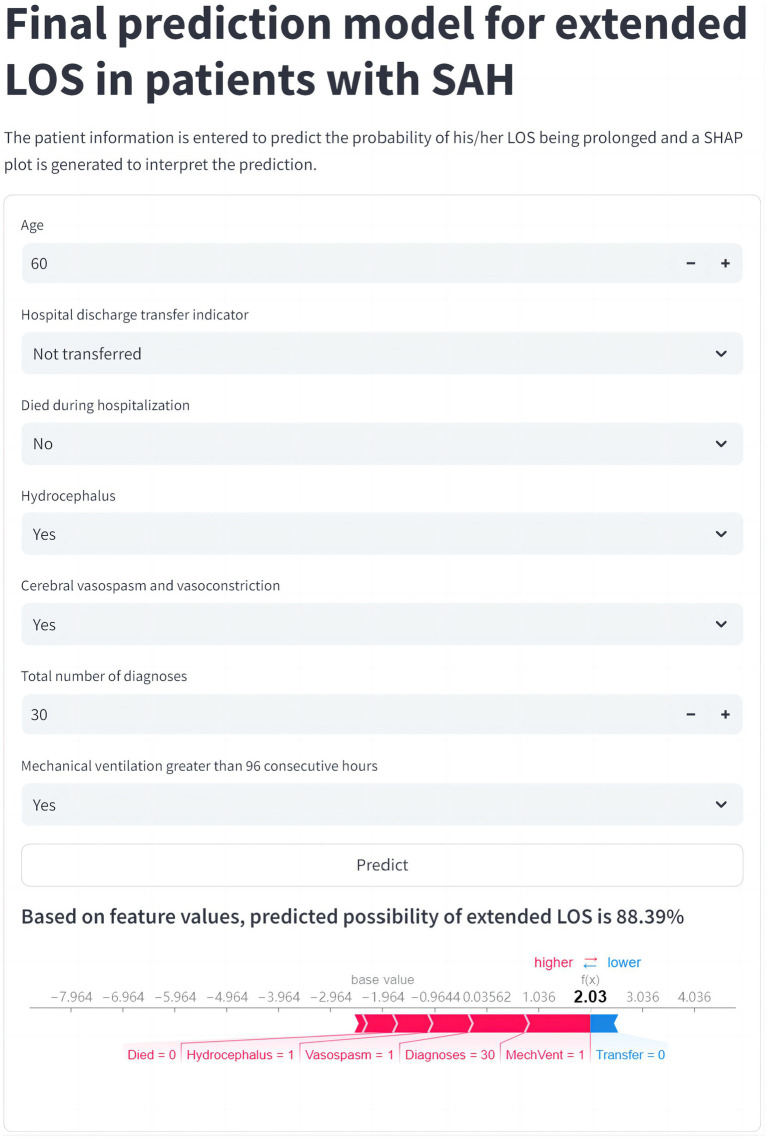
Web application for clinical utility of the extended LOS prediction model. The final CatBoost model, designed to predict the risk of extended LOS, has been integrated into a user-friendly web application to support clinical decision-making. Upon entering the actual values for the 7 key features, the application automatically generates a risk prediction for extended LOS for the individual patient. The resulting force plot, displayed within the application, visually represents the features influencing the prediction. Red features on the left indicate factors pushing the prediction toward extended LOS, while blue features on the right represent those pushing toward normal LOS. This web application can be accessed online at https://prediction-model-aodpzmcnilelv9yb3eabo5.streamlit.app. CatBoost, categorical boosting; LOS, length of stay; SHAP, SHapley Additive exPlanations.

The final prediction model was implemented in a web application to facilitate its utility in clinical scenarios, as shown in Figure 6. When the actual values of the 7 features required for the model are entered, this application automatically predicts the risk of extended LOS for an individual patient and provides an explanation for that specific prediction via a SHAP force plot. This plot visually decomposes how each feature value contributes to shifting the model’s output from a baseline (the average prediction over the dataset) to the final score. Features in red (left side) increase the predicted risk of extended LOS, while features in blue (right side) decrease it. The cumulative effect of all features determines the final position on the output axis, which corresponds to the predicted probability (e.g., 88.39%). This intuitive explanation allows clinicians to understand the key drivers behind the risk assessment for each patient.

### Analysis of the relationship between LOS and total charges

**Figure 7:**
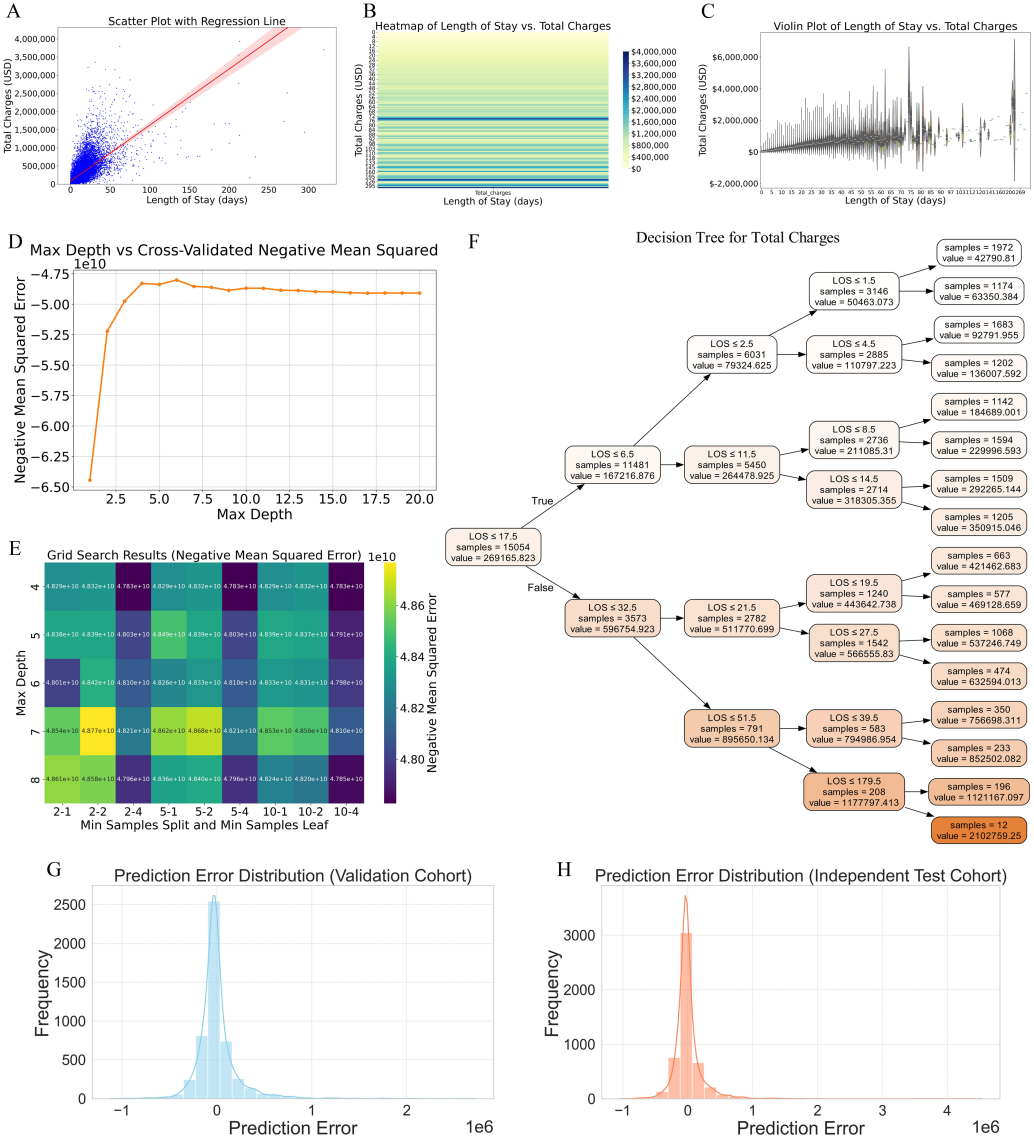
Analysis of the relationship between LOS and total hospital charges, and construction of a predictive model based on LOS. **(A)** Scatter plot with a regression line illustrating the positive correlation between LOS and total hospital charges. The shaded area around the regression line represents the confidence interval, highlighting the strong linear relationship. **(B)** Heatmap visualizing the distribution of total charges across various lengths of stay. Darker shades indicate higher concentrations of data points, confirming the trend that longer hospital stays are associated with higher total charges. **(C)** Violin plot detailing the distribution and variability of total charges for different LOS ranges. This plot reveals that longer stays generally result in higher charges and that there is greater variability in charges for extended LOS, suggesting the influence of individual patient factors and specific treatments. **(D)** Cross-validated negative mean square error plot identifying the optimal maximum depth for the decision tree model, indicating that the error stabilizes between depths of 4 and 8. **(E)** Grid search results confirming that a maximum depth of 4 yields the lowest error for the decision tree model. **(F)** Decision tree diagram constructed with the optimal hyperparameters, depicting how different LOS intervals predict total charges, with specific charge estimates provided at each decision node. **(G)** Prediction error distribution for the validation cohort, showing errors centered around zero, indicating generally accurate model predictions. **(H)** Prediction error distribution for the independent test cohort, demonstrating a narrow distribution of errors, suggesting robust performance and good generalizability to new data. LOS, length of stay.

Our analysis revealed a clear positive correlation between LOS and total hospital charges. The scatter plot with a regression line (Figure 7A) indicated that as the LOS increased, the total charges also tended to rise, with the regression line highlighting this strong linear relationship. The shaded area around the regression line represents the confidence interval, reinforcing the reliability of this correlation. To further illustrate this relationship, a heatmap (Figure 7B) with a gradient color scheme was used to show the distribution of total charges across various lengths of stay, with darker shades indicating higher concentrations of data points. This visualization confirms the trend that longer hospital stays were associated with higher total charges. Additionally, the violin plot (Figure 7C) provides a detailed visualization of the distribution and variability of total charges for different LOS ranges. This reveals that not only did longer stays generally result in higher charges, but there was also greater variability in the charges for extended LOS, suggesting that individual patient factors and specific treatments significantly influence the total cost.

### Construction and validation of a predictive model for total charges based on LOS

To construct a predictive model for total charges based on LOS, we employed the decision tree method because of its interpretability and ability to capture nonlinear relationships without extensive preprocessing. The optimal hyperparameters for the decision tree were determined via cross-validated negative mean square error (Figure 7D) and grid search (Figure 7E). The cross-validated negative mean square error plot revealed that the optimal maximum depth for the decision tree lay between 4 and 8, as the error stabilized within this range. Further refinement via grid search confirmed that a maximum depth of 4 yielded the lowest error. The final decision tree model, constructed using these optimal hyperparameters, is depicted in the decision tree diagram (Figure 7F). This model clearly delineates how different LOS intervals predict total charges, with specific charge estimates provided at each decision node. For example, patients with an LOS of ≤6.5 days typically incurred lower charges, whereas those with longer stays exhibited a tiered increase in charges, making the model highly interpretable and clinically useful.

The prediction error distributions for both the validation and independent test cohorts are illustrated in the respective plots (Figures 7G,H), showing errors centered around zero. This characteristic indicates that the model’s predictions are generally accurate, and the narrow distribution of errors suggests robust performance and good generalizability to new data.

### Factors associated with high hospital charges in patients with extended LOS

As shown in Supplementary Table 8, univariate analysis identified 59 variables with statistically significant differences (*p* < 0.05) among the 90 variables considered. These variables were then included in a multivariate logistic regression model, which narrowed down the factors to 22 variables. Subsequently, these 22 variables were subjected to LASSO regression. Supplementary Figures 7A,B present the LASSO coefficient path and cross-validation deviation plots, respectively. The final set of factors associated with high hospital charges is presented in Supplementary Figure 7C. The variables positively correlated with high charges, in descending order of influence, were: Total number of procedures, Respiratory failure, Tracheostomy, Hospital region, Cerebral vasospasm and vasoconstriction, Sepsis, Pulmonary infection, Race, and Hospital control/ownership. Negative correlations were found with: Fluoroscopy of artery, Age, and Monitoring of arterial pressure.

## Discussion

### Study overview and methodological foundation

This study is among the first to comprehensively construct and validate ML models specifically designed to predict the likelihood of extended LOS in patients diagnosed with SAH. Additionally, we explored the relationship between LOS and total hospital charges, which led to the construction of a predictive model that estimates hospital costs on the basis of LOS. Leveraging data from a large, nationally representative inpatient database, we identified key predictive factors and constructed ML models that integrate both clinical and demographic data. These models were validated on an independent test cohort, offering clinicians valuable tools for making informed decisions in the management of SAH patients, thereby improving patient outcomes and optimizing the allocation of healthcare resources.

The potential of ML to predict clinical outcomes such as extended LOS and hospital costs has been widely recognized for its ability to process complex and diverse datasets, uncovering intricate patterns and relationships that traditional methods may overlook (19–21). This capability is particularly advantageous in clinical environments with large sample sizes and significant variability in patient demographics and clinical characteristics (21, 22). In our study, we rigorously constructed and validated predictive models for extended LOS and total hospital charges in patients with SAH using data from the NIS. Among the 12 ML models evaluated, the CatBoost algorithm emerged as the most effective for predicting extended LOS. Renowned for its ability to handle categorical data and prevent overfitting, the CatBoost model in our study incorporated 7 key variables, achieving an optimal balance between feature quantity and predictive performance (23). The model demonstrated superior AUC values, excellent calibration, and strong clinical applicability, making it a powerful tool for predicting extended LOS in clinical practice. Additionally, we employed a decision tree model to predict hospital charges on the basis of LOS; this model was selected for its interpretability and ability to handle nonlinear relationships without extensive preprocessing (16, 21).

Selecting the optimal number of features for predictive models is challenging, particularly in the absence of established guidelines. While more features can potentially improve accuracy, they may also introduce noise and reduce the model’s clinical utility (24, 25). To ensure a rigorous and statistically sound selection of the most relevant variables, we employed a comprehensive feature selection process. This process began with univariate analysis and correlation analysis, followed by LASSO regression and RFE. Finally, we utilized the SHAP method to refine the selection further, thereby ensuring that only the most predictive and clinically relevant variables were included (26). Our final CatBoost model, which incorporates seven key variables, strikes a balance between predictive performance and simplicity, making it both robust and easy to integrate into clinical practice. This streamlined approach enhances the model’s applicability, providing clinicians with a practical tool for improving decision-making in the management of SAH patients.

### Interpretation of key predictors for extended LOS

The variables included in the final CatBoost model, ranked by importance, are as follows: Total number of diagnoses, Hydrocephalus, Died during hospitalization, Hospital discharge transfer indicator, Mechanical ventilation for greater than 96 consecutive hours, Cerebral vasospasm and vasoconstriction, and Age. The total number of diagnoses emerged as the most crucial predictor, with a greater number of diagnoses strongly correlating with extended LOS owing to the complexity of managing multiple comorbidities, which necessitated additional treatments and prolonged monitoring. This association between comorbidity burden and extended hospitalization has been consistently supported across various studies (27, 28). Hydrocephalus, a common and severe complication of SAH, is closely associated with extended LOS, as its management often requires surgical intervention, such as ventriculoperitoneal shunting, and ongoing monitoring, which naturally prolongs hospitalization. The significant impact of hydrocephalus on both morbidity and LOS in SAH and other neurological conditions has been widely reported (29). Conversely, the variable Died during hospitalization was associated with a tendency toward a normal LOS, reflecting the fact that patients who succumb during their hospital stay often have a shorter LOS because of rapid progression to death. This inverse relationship between mortality and LOS has been observed in various studies (28, 30). The Hospital discharge transfer indicator further differentiates LOS, as patients who are transferred to nonacute care facilities are more likely to have an extended LOS, whereas those who are transferred to acute care facilities or are not transferred generally experience a normal LOS. This reflects the need for ongoing care in less intensive settings, which aligns with studies on discharge practices (31). Mechanical ventilation for more than 96 consecutive hours indicates severe illness and is strongly associated with extended LOS due to complications such as ventilator-associated pneumonia, which prolong ICU stays. This relationship is robustly supported in evaluations of critically ill patients (32). Cerebral vasospasm and vasoconstriction, which are recognized complications of SAH, also contribute to an extended LOS owing to the need for intensive monitoring and medical interventions to manage delayed cerebral ischemia. The association between cerebral vasospasm and prolonged hospitalization is well established in the literature (3, 33). Finally, the variable Age was inversely related to LOS, with older patients being more likely to have shorter or normal hospital stays. This may have been due to a clinical approach that favors less aggressive treatment in elderly patients or an inclination toward earlier discharge to home or palliative care settings. The literature indicates that age influences treatment decisions and LOS, with older patients often receiving less intensive care and having shorter hospitalizations (31, 34). By integrating these variables, our CatBoost model provides valuable insights into the clinical factors driving prolonged hospitalization in SAH patients, enhancing predictive accuracy and supporting informed decision-making in patient management.

### Model performance, advantages, and clinical translation

Compared with the published methods for predicting extended LOS in SAH patients, our model offers significant advancements in terms of both predictive accuracy and clinical applicability (35). Previous studies have often been focused on narrower aspects of SAH management, such as predicting specific complications or outcomes in selected patient cohorts, and have utilized various ML models with mixed results (20, 36). These investigations, while valuable, typically address only parts of the broader challenge of managing SAH, particularly in relation to prolonged hospitalization. In contrast, our model integrates a wider array of clinically relevant variables, providing a more comprehensive tool for predicting extended LOS. Moreover, while earlier models have often lacked full transparency and clinical interpretability, our use of the SHAP method to elucidate the contribution of each variable enhances the model’s utility in clinical settings, allowing practitioners to understand and trust the model’s predictions. This contrasts with many previous studies where the “black box” nature of ML models has limited their practical application (37, 38). Furthermore, our model’s robust performance (AUC of 0.904 in internal validation and 0.910 in hold-out internal validation) underscores its generalizability and reliability, which are critical for clinical adoption.

In addition to its strong predictive performance, the clinical applicability of our model is enhanced by the transparent and interpretable nature of the SHAP method, which addresses one of the key limitations often associated with ML models. Traditional ML models, while powerful, have been criticized for their opacity and are often referred to as “black-box” models because of the difficulty in understanding how specific predictions are made (39). This lack of transparency can lead to hesitation among clinicians to integrate these tools fully into clinical decision-making processes. By employing the SHAP method, we provide both global and local explanations for the model’s predictions. SHAP allows us to determine which variables are most influential across the entire patient population, while locally, it provides insights into how specific predictions are made for individual patients on the basis of their unique data inputs. This dual-level explanation fosters greater trust in the model’s outputs and facilitates its adoption in clinical settings where understanding the rationale behind predictions is crucial (16, 40). Furthermore, SHAP analysis can directly influence clinical decision-making. By identifying high-risk patients for extended LOS, clinicians can prioritize early interventions like tracheostomy or rehabilitation, improving outcomes and reducing costs. Recognizing key features, such as respiratory failure or cerebral vasospasm, allows for more efficient resource allocation, ensuring ICU beds and staff are available for those most in need. This approach optimizes ICU bed management and aligns with value-based care, aiming to improve patient outcomes while controlling healthcare costs.

Moreover, the practical utility of our model is further enhanced by the development of a web-based tool using the Streamlit framework, which allows easy access and broader dissemination among clinicians. This tool enables healthcare providers to input patient data and receive predictions about the likelihood of extended LOS in real time, accompanied by explanations that can guide clinical decision-making (41). The integration of such user-friendly platforms ensures that advanced predictive models are not confined to academic research but are accessible in everyday clinical practice, thereby improving patient care. Compared with earlier studies that either lacked such tools or provided limited interpretability, our approach represents a significant step forward in making ML models more applicable and valuable in the real-world management of SAH (42). By bridging the gap between advanced analytics and clinical usability, our model sets a new standard for the application of ML in predicting extended LOS, offering both accuracy and transparency, which are essential for effective healthcare delivery (7, 11).

### Economic implications of LOS and cost prediction

The observed positive correlation between LOS and total hospital charges incurred by SAH patients underscores the significant impact that prolonged hospitalization has on healthcare costs (4). This relationship highlights the importance of addressing factors that contribute to extended LOS, as they are directly linked to increased resource utilization and increased financial burden on healthcare systems. Consistent with our findings, previous studies have demonstrated that longer hospital stays are associated with increased costs, not only because of the direct expenses of prolonged bed occupancy but also because of the more intensive care required for managing the complications and comorbidities that often accompany extended stays (10, 11). The decision tree model constructed in our study offers a robust tool for predicting total hospital charges on the basis of LOS, with high accuracy across both internal and hold-out internal validation cohorts. The ability of this model to delineate specific LOS intervals and their associated costs provides valuable insights that can inform clinical decision-making and resource allocation. By identifying patients who are at risk for extended stays and higher costs early in their hospital course, healthcare providers can prioritize targeted interventions aimed at reducing LOS, such as enhanced monitoring, early mobilization, and proactive management of potential complications. This approach aligns with the growing emphasis on value-based care (in which the goal is to improve patient outcomes while controlling costs) and has been shown to be effective in reducing overall healthcare expenditures without compromising the quality of care (43, 44). Furthermore, the interpretability of the decision tree model, which clearly illustrates how different LOS intervals contribute to total costs, allows healthcare administrators to better forecast hospital budgets and develop cost-saving strategies that are both data-driven and patient-centered (21).

The factors associated with high hospital charges in patients with extended LOS reflect the complexity of their conditions. Severe complications such as respiratory failure, sepsis, and the total number of procedures are primary drivers of increased costs, as these require intensive care and prolonged hospitalization (4, 21, 45). Regional and ownership-related variations in healthcare infrastructure also contribute to cost differences (46). Interestingly, age and certain procedures like fluoroscopy of the artery were negatively correlated with charges, possibly due to more conservative treatment approaches for older patients and the relatively short duration of some high-cost procedures. These findings highlight the importance of early identification and management of high-risk patients to optimize care and reduce unnecessary costs (47).

Moreover, the findings of this study underscore the potential of predictive models to support the implementation of clinical pathways designed to reduce unnecessary delays and optimize resource use. By incorporating such models into routine clinical practice, healthcare institutions can more effectively manage the care of SAH patients, potentially reducing the length of stay and associated costs. For example, identifying the key drivers of extended LOS through predictive modeling could lead to the development of standardized protocols for the early identification and treatment of complications, which in turn could shorten hospital stays and reduce costs. This approach is supported by evidence from other areas of healthcare, where predictive analytics have been successfully integrated into clinical workflows to improve efficiency and patient outcomes (48, 49). The ability to predict and manage LOS and associated costs not only enhances the quality of care but also aligns with broader healthcare goals of sustainability and cost-effectiveness, particularly in resource-intensive settings such as neurosurgery and critical care (4, 50). Thus, our study not only helps to elucidate the economic burden associated with prolonged LOS in SAH patients but also provides a practical framework for leveraging predictive models to achieve better outcomes in both patient care and financial management.

### Limitations and future directions

We acknowledge key limitations in this study. First, our choice of well-established algorithms (CatBoost and Decision Tree) prioritizes interpretability and robustness on structured data, which is crucial for clinical trust. However, this represents a trade-off, as these models may not capture the most complex, non-linear patterns compared to advanced deep learning architectures, indicating a limitation in methodological frontier exploration. Second, and most critically, our models are derived from U.S. data (NIS), and their direct applicability to populations with fundamentally different healthcare systems—such as China—is severely constrained. This limitation extends beyond genetics to profound differences in clinical pathways, insurance schemes, resource availability, and socioeconomic determinants of health. Consequently, the model’s utility for local clinical decision-making in other settings is unverified and requires deliberate adaptation. Third, the retrospective design lacks granular clinical details and may not account for all relevant confounders.

Future research must bridge these gaps to translate findings into practical, localized utility. (1) The immediate priority is external validation and calibration using high-quality, multi-center data from the target population (e.g., in China) to ensure relevance. (2) Concurrently, exploring and benchmarking more sophisticated models (e.g., deep learning) on enriched datasets is warranted to assess potential gains. (3) The long-term goal should be the development and prospective testing of context-specific, clinically integrated decision-support tools that dynamically learn from local practice.

## Conclusion

In this study, we constructed and validated robust and interpretable ML models to predict extended LOS and associated hospital charges in adult patients with SAH. By utilizing the CatBoost algorithm, our model demonstrated strong predictive accuracy and clinical applicability, successfully identifying key factors contributing to extended hospitalization. Furthermore, the decision tree model and subsequent cost analysis provided valuable insights into the financial implications of prolonged LOS, specifically identifying the patient subgroup at highest risk for incurring disproportionately high costs. These findings collectively emphasize the potential of such models to guide early and targeted interventions, aiming to optimize patient outcomes and healthcare resource allocation. Despite these promising results, the retrospective design of this investigation and the limitations inherent to the NIS database suggest the need for future prospective, multicenter studies to increase the generalizability and real-world applicability of these models.

## Data Availability

The data used in this study are publicly available from the National Inpatient Sample (NIS) database, part of the Healthcare Cost and Utilization Project (HCUP) in the United States. Access to the NIS database is governed by the HCUP data use agreement, and users are required to complete training and certification. The data are not owned by the authors and can be obtained directly from HCUP (https://www.hcup-us.ahrq.gov/), or upon reasonable request to the corresponding author, with the appropriate permissions from HCUP.
