# Supplementary material for: Machine learning models for predicting extended length of stay and hospital charges in nontraumatic subarachnoid hemorrhage
Source: Front Neurol. 2026 Feb 4;17:1737503. doi: 10.3389/fneur.2026.1737503 (PMC12913072; doi:10.3389/fneur.2026.1737503)
Supplement: Supplementary file 1 [file Table_1.docx]

| **Supplementary table 1. Codes corresponding to the diagnoses and procedures collected** | | |
| --- | --- | --- |
| Variable | ICD-10-CM code | ICD-10-PCS code |
| Nontraumatic subarachnoid hemorrhage | I60 |  |
| Hypertension | I10-I15 |  |
| Type II diabetes | E11 |  |
| Coronary heart disease | I20-I25 |  |
| Atrial fibrillation | I48.0-I48.2, I48.91 |  |
| Hyperlipidemia | E78.0-E78.5 |  |
| Elevated blood glucose level | R73 |  |
| Chronic obstructive pulmonary disease | J44 |  |
| Hypothyroidism | E03 |  |
| Anxiety | F41 |  |
| Depression | F32, F33 |  |
| Overweight and obesity | E66 |  |
| Tobacco use | F17.2, Z87.891, Z72.0 |  |
| Alcohol abuse | F10, Z86.41 |  |
| History of transient ischemic attack and cerebral infarction | Z86.73 |  |
| Long term (current) use of anticoagulants and antithrombotic/antiplatelets | Z79.0 |  |
| Long term (current) use of aspirin | Z79.82 |  |
| Contact with and (suspected) exposure to communicable diseases | Z20 |  |
| Kidney failure | N17-N19 |  |
| Hepatic failure | K72 |  |
| Paralytic | G83 |  |
| Disorders of fluid, electrolyte and acid-base balance | E87 |  |
| Shock | R57 |  |
| Respiratory failure | J96 |  |
| Convulsions | R56 |  |
| Muscle spasm | M62.83 |  |
| Pulmonary infection | J12-J16, J18 |  |
| Urinary tract infection | N39.0 |  |
| Intracranial infection | G00-G06 |  |
| Sepsis | A41 |  |
| Cerebral edema | G93.6 |  |
| Hydrocephalus | G91 |  |
| Nausea and vomiting | R11 |  |
| Headache | R51 |  |
| Anemia | D50-D64 |  |
| Gastro-esophageal reflux | K21 |  |
| Dysphagia | R13.1 |  |
| Aphasia | R47.01 |  |
| Nontraumatic intracerebral hemorrhage | I61, I62 |  |
| Elevated white blood cell count | D72.82 |  |
| Thrombocytopenia | D69.6 |  |
| Facial weakness | R29.810 |  |
| Embolism and thrombosis of deep veins of lower extremity | I82.4, I82.5 |  |
| Cerebral aneurysm, no ruptured | I67.1 |  |
| Cerebrovascular arteriovenous malformation | Q28.2 |  |
| Disordered phosphorus metabolism | E83.3 |  |
| Disordered magnesium metabolism | E83.4 |  |
| Cerebral vasospasm and vasoconstriction | I67.84 |  |
| Constipation | K59.0 |  |
| Occlusion of intracranial Artery |  | 03LG*** |
| Restriction of intracranial artery |  | 03VG*** |
| Excision of intracranial artery |  | 03BG*** |
| Bypass operation of intracranial arteries |  | 031G*** |
| Monitoring of arterial pulse |  | 4A130J*, 4A133J*, 4A13XJ* |
| Monitoring of arterial pressure |  | 4A130B*, 4A133B*, 4A13XB* |
| Monitoring of central nervous electrical activity |  | 4A1004*, 4A1034*, 4A1074*, 4A1084*, 4A10X4* |
| Percutaneous ventriculostomy |  | 009630Z, 00963ZZ, 00H632Z |
| Airway intubation |  | 0BH17EZ, 0BH18EZ |
| Tracheostomy |  | 0B110F4, 0B113F4,  0B114F4 |
| Mechanical ventilation |  |  |
| Less than 24 consecutive hours |  | 5A1935Z |
| 24-96 consecutive hours |  | 5A1945Z |
| Greater than 96 consecutive hours |  | 5A1955Z |
| Insertion of feeding device into stomach |  | 0DH6*UZ |
| Introduction of nutritional substance into upper GI |  | 3E0G36Z, 3E0G76Z,  3E0G86Z |
| Lumbar puncture |  | 009U3*, 00JU3ZZ |
| Insertion of infusion device into superior vena cava |  | 02HV03Z, 02HV33Z , 02HV43Z |
| Insertion of monitoring device into upper artery |  | 03HY02Z, 03HY32Z, 03HY42Z |
| Ultrasonography of superior vena cava |  | B548** |
| Fluoroscopy of artery |  | B31****，B41**** |
| Administration of thrombolytics and platelet inhibitors |  | 3E0**16, 3E0**17, 3E0**PZ, |
| Transfusion of blood and blood products |  | 3023**H*, 3023**N*, 3023**P*, 3023**Q*, 3023**R*, 3024**H*, 3024**N*, 3024**P*, 3024**Q*, 3024**R*, 3025**H*, 3025**N*, 3025**P*, 3025**Q*, 3025**R* |
| ICD-10-CM: International Classification of Diseases, Tenth Revision, Clinical Modification; ICD-10-PCS: ICD-10 Procedure Coding System; GI: gastrointestinal.  The symbol “-” indicates a range of codes from the starting code to the ending code.  The symbol “*” represents a single character, which can be either a letter or a number.  GI: gastrointestinal. | | |
