# Supplementary material for: Machine learning models for predicting extended length of stay and hospital charges in nontraumatic subarachnoid hemorrhage
Source: Front Neurol. 2026 Feb 4;17:1737503. doi: 10.3389/fneur.2026.1737503 (PMC12913072; doi:10.3389/fneur.2026.1737503)
Supplement: Supplementary file 2 [file Table_2.docx]

| **Supplementary table S2. Number of actual and missing patients for each variable and their proportion within the actual patient population** | | | |
| --- | --- | --- | --- |
| Variables | Actual Patients | Missing Patients | Proportion |
| **Patient Demographics** |  |  |  |
| Age (years) | 25092 | 0 |  |
| Mean ± SD |  |  | 59.6±16.2 |
| Median (IQR) |  |  | 60.0(49.0,71.0) |
| Gender (%) | 25090 | 2 |  |
| Females |  |  | 15290(60.9) |
| Males |  |  | 9800(39.1) |
| Race (%) | 23938 | 1154 |  |
| White |  |  | 14430(57.5) |
| Black |  |  | 3863(15.4) |
| Hispanic |  |  | 3274(13) |
| Other |  |  | 2371(9.4) |
| Length of stay (days) | 25089 | 3 |  |
| Mean ± SD |  |  | 11.4±74.3 |
| Median (IQR) |  |  | 9.0(3.0, 17.0) |
| Total charges (dollars) | 24802 | 290 |  |
| Mean ± SD |  |  | 269153.1±312267.3 |
| Median (IQR) |  |  | 174235.5(60658.3, 359457.0) |
| Median household income quartile (%) | 24597 | 495 |  |
| 0–25th |  |  | 7235(28.8) |
| 26–50th |  |  | 6286(25.1) |
| 51–75th |  |  | 5882(23.4) |
| 76–100th |  |  | 5194(20.7) |
| Primary expected payer (%) | 25026 | 66 |  |
| Medicare |  |  | 9678(38.6) |
| Medicaid |  |  | 4068(16.2) |
| Private insurance |  |  | 8639(34.4) |
| Other |  |  | 2641(10.5) |
| Non-elective admission | 25040 | 52 | 23970(95.5) |
| Hospitalization year (%) |  |  |  |
| 2016 |  |  | 5037(20.1) |
| 2017 |  |  | 5046(20.1) |
| 2018 |  |  | 5085(20.3) |
| 2019 |  |  | 5140(20.5) |
| 2020 |  |  | 4784(19.1) |
| Hospitalization season (%) | 25077 | 15 |  |
| Spring (March-May) |  |  | 6174(24.6) |
| Summer (June-August) |  |  | 6077(24.2) |
| Fall (September-November) |  |  | 6372(25.4) |
| Winter (December-February) |  |  | 6454(25.7) |
| Hospitalization on weekends (%) | 25092 | 0 | 6671(26.6) |
| Hospital admission transfer indicator (%) |  | 93 |  |
| Not transferred/Standard admission |  |  | 14078(56.1) |
| From acute care hospital |  |  | 9828(39.2) |
| From other facility |  |  | 1093(4.4) |
| Hospital discharge transfer indicator (%) |  | 15 |  |
| Not transferred |  |  | 16302(65) |
| To acute care hospital |  |  | 1259(5) |
| To other facility |  |  | 7516(30) |
| Died during hospitalization | 25077 | 15 | 4679(18.6) |
| **Hospital demographics** (%) | 25092 | 0 |  |
| Hospital region |  |  |  |
| Northeast |  |  | 4377(17.4) |
| Midwest |  |  | 4894(19.5) |
| South |  |  | 9911(39.5) |
| West |  |  | 5910(23.6) |
| Hospital bed size |  |  |  |
| Small |  |  | 1867(7.4) |
| Medium |  |  | 5212(20.8) |
| Large |  |  | 18013(71.8) |
| Hospital location/teaching status |  |  |  |
| Rural |  |  | 423(1.7) |
| Urban nonteaching |  |  | 2287(9.1) |
| Urban teaching |  |  | 22382(89.2) |
| Hospital control/ownership (%) |  |  |  |
| Government, nonfederal |  |  | 3565(14.2) |
| Private, not-profit |  |  | 18729(74.6) |
| Private, invest-own |  |  | 2798(11.2) |
| **Diagnosis, symptoms and complications on admission and during hospitalization** (%) | 25092 | 0 |  |
| Hypertension |  |  | 17537(69.9) |
| Type Ⅱ diabetes |  |  | 4381(17.5) |
| Coronary heart disease |  |  | 3839(15.3) |
| Atrial fibrillation |  |  | 2611(10.4) |
| Hyperlipidemia |  |  | 7887(31.4) |
| Elevated blood glucose level |  |  | 2232(8.9) |
| Chronic obstructive pulmonary disease |  |  | 1965(7.8) |
| Hypothyroidism |  |  | 2344(9.3) |
| Anxiety |  |  | 2367(9.4) |
| Depression |  |  | 2347(9.4) |
| Overweight and obesity |  |  | 3066(12.2) |
| Tobacco use |  |  | 9050(36.1) |
| Alcohol abuse |  |  | 1353(5.4) |
| History of transient ischemic attack and cerebral infarction |  |  | 1659(6.6) |
| Long term (current) use of anticoagulants and antithrombotic/antiplatelets |  |  | 2019(8.0) |
| Long term (current) use of aspirin |  |  | 2846(11.3) |
| Contact with and (suspected) exposure to communicable diseases |  |  | 1264(5.0) |
| Kidney failure |  |  | 4060(16.2) |
| Hepatic failure |  |  | 228(0.9) |
| Paralytic |  |  | 325(1.3) |
| Disorders of fluid, electrolyte and acid-base balance |  |  | 11805(47.0) |
| Shock |  |  | 1076(4.3) |
| Respiratory failure |  |  | 9073(36.2) |
| Convulsions |  |  | 1776(7.1) |
| Muscle spasm |  |  | 132(0.5) |
| Pulmonary infection |  |  | 2427(9.7) |
| Urinary tract infection |  |  | 2965(11.8) |
| Intracranial infection |  |  | 570(2.3) |
| Sepsis |  |  | 1257(5.0) |
| Cerebral edema |  |  | 6940(27.7) |
| Hydrocephalus |  |  | 9133(36.4) |
| Nausea and vomiting |  |  | 656(2.6) |
| Headache |  |  | 3427(13.7) |
| Anemia |  |  | 5493(21.9) |
| Gastro-esophageal reflux |  |  | 3036(12.1) |
| Dysphagia |  |  | 2836(11.3) |
| Aphasia |  |  | 2172(8.7) |
| Nontraumatic intracerebral hemorrhage |  |  | 6128(24.4) |
| Elevated white blood cell count |  |  | 2500(10.0) |
| Thrombocytopenia |  |  | 1036(4.1) |
| Facial weakness |  |  | 1407(5.6) |
| Embolism and thrombosis of deep veins of lower extremity |  |  | 693(2.8) |
| Cerebral aneurysm, no ruptured |  |  | 2350(9.4) |
| Cerebrovascular arteriovenous malformation |  |  | 294(1.2) |
| Disordered phosphorus metabolism |  |  | 3745(14.9) |
| Disordered magnesium metabolism |  |  | 1501(6.0) |
| Cerebral vasospasm and vasoconstriction |  |  | 4786(19.1) |
| Constipation |  |  | 1627(6.5) |
| Total number of diagnoses |  |  |  |
| Mean ± SD |  |  | 15.3±7.0 |
| Median (IQR) |  |  | 15.0(10.0,20.0) |
| **Procedures during hospitalization**(%) | 25092 | 0 |  |
| Occlusion of intracranial artery |  |  | 3932(15.7) |
| Restriction of intracranial artery |  |  | 5462(21.8) |
| Excision of intracranial artery |  |  | 176(0.7) |
| Bypass operation of intracranial arteries |  |  | 44(0.2) |
| Monitoring of arterial pulse |  |  | 1017(4.1) |
| Monitoring of arterial pressure |  |  | 1835(7.3) |
| Monitoring of central nervous electrical activity |  |  | 1278(5.1) |
| Percutaneous ventriculostomy |  |  | 6088(24.3) |
| Airway intubation |  |  | 5010(20.0) |
| Tracheostomy |  |  | 754(3.0) |
| Mechanical ventilation |  |  |  |
| Less than 24 consecutive hours |  |  | 2171(8.7) |
| 24-96 consecutive hours |  |  | 3210(12.8) |
| Greater than 96 consecutive hours |  |  | 3625(14.4) |
| Lumbar puncture |  |  | 1210(4.8) |
| Insertion of feeding device into stomach |  |  | 2019(8.0) |
| Introduction of nutritional substance into upper GI |  |  | 938(3.7) |
| Insertion of monitoring device into upper artery |  |  | 2386(9.5) |
| Insertion of infusion device into superior vena cava |  |  | 4857(19.4) |
| Ultrasonography of superior vena cava |  |  | 914(3.6) |
| Fluoroscopy of artery |  |  | 11088(44.2) |
| Administration of thrombolytics and platelet inhibitors |  |  | 440(1.8) |
| Transfusion of blood and blood products |  |  | 1370(5.5) |
| Total number of procedures |  |  |  |
| Mean ± SD |  |  | 5.6±5.3 |
| Median (IQR) |  |  | 4.0(2.0,8.0) |
| Continuous variables were presented as means [standard deviation (SD)] or medians [interquartile range (IQR)]. Categorical variables were presented as numbers (percentage).  GI: gastrointestinal | | | |
