# Supplementary material for: Machine learning models for predicting extended length of stay and hospital charges in nontraumatic subarachnoid hemorrhage
Source: Front Neurol. 2026 Feb 4;17:1737503. doi: 10.3389/fneur.2026.1737503 (PMC12913072; doi:10.3389/fneur.2026.1737503)
Supplement: Supplementary file 3 [file Table_3.docx]

| **Supplementary table S3. Comparison of patient characteristics between development and test cohorts, and within the development cohort between training and validation cohorts** | | | | | |
| --- | --- | --- | --- | --- | --- |
| Variables | Development Cohort  (80%, N=20073) | | Test cohort  (20%, N=5019) | P^#^ |  |
|  | Training cohort  (75%, N=15054) | Validation cohort  (25%, N=5019) |  |  | P^*^ |
| **Patient Demographics** | | | | | |
| Age (years) |  |  |  | 0.095 | 0.233 |
| Mean ± SD | 59.4±16.2 | 59.7±16.1 | 59.9±16.1 |  |  |
| Median (IQR) | 60.0(49.0,71.0) | 60.0(50.0,72.0) | 60.0(50.0,72.0) |  |  |
| Gender (%) |  |  |  | 0.912 | 0.093 |
| Females | 9121(60.6) | 3108(61.9) | 3062(61.0) |  |  |
| Males | 5933(39.4) | 1911(38.1) | 1957(39.0) |  |  |
| Race (%) |  |  |  | 0.856 | 0.644 |
| White | 9090(60.4) | 2997(59.7) | 3054(60.8) |  |  |
| Black | 2436(16.2) | 806(16.1) | 792(15.8) |  |  |
| Hispanic | 2039(13.5) | 691(13.8) | 679(13.5) |  |  |
| Other | 1489(9.9) | 525(10.5) | 494(9.8) |  |  |
| Length of stay (days) |  |  |  | 0.897 | 0.577 |
| Mean ± SD | 12.2±13.9 | 12.3±12.9 | 12.2±13.3 |  |  |
| Median (IQR) | 9.0(3.0,17.0) | 9.0(3.0,17.0) | 9.0(3.0,17.0) |  |  |
| Total charges (dollars) |  |  |  | 0.640 | 0.672 |
| Mean ± SD | 269165.8±312524.3 | 270825±307386.5 | 268453.8±315431.7 |  |  |
| Median (IQR) | 173856.0(61413.5,360480.0) | 178150.0(59577.5,362816.0) | 172586.0(61615.0,354737.5) |  |  |
| Median household income quartile (%) |  |  |  | 0.258 | 0.295 |
| 0–25th | 4523(30.0) | 1436(28.6) | 1420(28.3) |  |  |
| 26–50th | 3814(25.3) | 1298(25.9) | 1297(25.8) |  |  |
| 51–75th | 3558(23.6) | 1210(24.1) | 1232(24.5) |  |  |
| 76–100th | 3159(21.0) | 1075(21.4) | 1070(21.3) |  |  |
| Primary expected payer (%) |  |  |  | 0.094 | 0.128 |
| Medicare | 5748(38.2) | 1971(39.3) | 1980(39.5) |  |  |
| Medicaid | 2506(16.6) | 777(15.5) | 794(15.8) |  |  |
| Private insurance | 5215(34.6) | 1769(35.2) | 1681(33.5) |  |  |
| Other | 1585(10.5) | 502(10.0) | 564(11.2) |  |  |
| Non-elective admission | 14431(95.9) | 4789(95.4) | 4798(95.6) | 0.658 | 0.177 |
| Hospitalization year (%) |  |  |  | 0.144 | 0.616 |
| 2016 | 2973(19.7) | 1024(20.4) | 1040(20.7) |  |  |
| 2017 | 3081(20.5) | 1008(20.1) | 957(19.1) |  |  |
| 2018 | 3045(20.2) | 1006(20.0) | 1034(20.6) |  |  |
| 2019 | 3128(20.8) | 1009(20.1) | 1003(20.0) |  |  |
| 2020 | 2827(18.8) | 972(19.4) | 985(19.6) |  |  |
| Hospitalization season (%) |  |  |  | 0.136 | 0.549 |
| Spring (March-May) | 3703(24.6) | 1228(24.5) | 1250(24.9) |  |  |
| Summer (June-August) | 3598(23.9) | 1231(24.5) | 1250(24.9) |  |  |
| Fall (September-November) | 3847(25.6) | 1237(24.6) | 1291(25.7) |  |  |
| Winter (December-February) | 3906(25.9) | 1323(26.4) | 1228(24.5) |  |  |
| Hospitalization on weekends (%) | 4035(26.8) | 1305(26) | 1331(26.5) | 0.904 | 0.265 |
| Hospital admission transfer indicator (%) |  |  |  | 0.438 | 0.534 |
| Not transferred/Standard admission | 8448(56.1) | 2858(56.9) | 2817(56.1) |  |  |
| From acute care hospital | 5929(39.4) | 1947(38.8) | 1998(39.8) |  |  |
| From other facility | 677(4.5) | 214(4.3) | 204(4.1) |  |  |
| Hospital discharge transfer indicator (%) |  |  |  | 0.750 | 0.746 |
| Not transferred | 9760(64.8) | 3278(65.3) | 3275(65.3) |  |  |
| To acute care hospital | 761(5.1) | 258(5.1) | 242(4.8) |  |  |
| To other facility | 4533(30.1) | 1483(29.5) | 1502(29.9) |  |  |
| Died during hospitalization | 2813(18.7) | 935(18.6) | 935(18.6) | 0.945 | 0.929 |
| **Hospital demographics** (%) | | | | | |
| Hospital region |  |  |  | 0.091 | 0.748 |
| Northeast | 2601(17.3) | 892(17.8) | 884(17.6) |  |  |
| Midwest | 2984(19.8) | 986(19.6) | 924(18.4) |  |  |
| South | 5926(39.4) | 1942(38.7) | 2043(40.7) |  |  |
| West | 3543(23.5) | 1199(23.9) | 1168(23.3) |  |  |
| Hospital bed size |  |  |  | 0.708 | 0.744 |
| Small | 1122(7.5) | 363(7.2) | 382(7.6) |  |  |
| Medium | 3153(20.9) | 1035(20.6) | 1024(20.4) |  |  |
| Large | 10779(71.6) | 3621(72.1) | 3613(72.0) |  |  |
| Hospital location/teaching status |  |  |  | 0.958 | 0.724 |
| Rural | 259(1.7) | 78(1.6) | 86(1.7) |  |  |
| Urban nonteaching | 1377(9.1) | 457(9.1) | 453(9.0) |  |  |
| Urban teaching | 13418(89.1) | 4484(89.3) | 4480(89.3) |  |  |
| Hospital control/ownership (%) |  |  |  | 0.936 | 0.803 |
| Government, nonfederal | 2132(14.2) | 722(14.4) | 711(14.2) |  |  |
| Private, not-profit | 11227(74.6) | 3747(74.7) | 3755(74.8) |  |  |
| Private, invest-own | 1695(11.3) | 550(11) | 553(11) |  |  |
| **Diagnosis, symptoms and complications on admission and during hospitalization** | | | | | |
| Hypertension | 10589(70.3) | 3496(69.7) | 3452(68.8) | 0.055 | 0.358 |
| Type Ⅱ diabetes | 2614(17.4) | 894(17.8) | 873(17.4) | 0.891 | 0.469 |
| Coronary heart disease | 2318(15.4) | 761(15.2) | 760(15.1) | 0.729 | 0.688 |
| Atrial fibrillation | 1546(10.3) | 543(10.8) | 522(10.4) | 0.989 | 0.270 |
| Hyperlipidemia | 4722(31.4) | 1564(31.2) | 1601(31.9) | 0.426 | 0.786 |
| Elevated blood glucose level | 1340(8.9) | 464(9.2) | 428(8.5) | 0.306 | 0.461 |
| Chronic obstructive pulmonary disease | 1187(7.9) | 385(7.7) | 393(7.8) | 0.998 | 0.625 |
| Hypothyroidism | 1398(9.3) | 472(9.4) | 474(9.4) | 0.780 | 0.804 |
| Anxiety | 1391(9.2) | 510(10.2) | 466(9.3) | 0.687 | 0.054 |
| Depression | 1426(9.5) | 468(9.3) | 453(9.0) | 0.372 | 0.756 |
| Overweight and obesity | 1873(12.4) | 598(11.9) | 595(11.9) | 0.379 | 0.325 |
| Tobacco use | 5424(36) | 1773(35.3) | 1853(36.9) | 0.160 | 0.367 |
| Alcohol abuse | 816(5.4) | 257(5.1) | 280(5.6) | 0.513 | 0.413 |
| History of transient ischemic attack and cerebral infarction | 1005(6.7) | 326(6.5) | 328(6.5) | 0.807 | 0.656 |
| Long term (current) use of anticoagulants and antithrombotic/antiplatelets | 1175(7.8) | 424(8.4) | 420(8.4) | 0.349 | 0.145 |
| Long term (current) use of aspirin | 1721(11.4) | 570(11.4) | 555(11.1) | 0.478 | 0.884 |
| Contact with and (suspected) exposure to communicable diseases | 744(4.9) | 259(5.2) | 261(5.2) | 0.556 | 0.539 |
| Kidney failure | 149(1) | 43(0.9) | 36(0.7) | 0.110 | 0.402 |
| Hepatic failure | 2412(16) | 851(17) | 797(15.9) | 0.518 | 0.121 |
| Paralytic | 205(1.4) | 53(1.1) | 67(1.3) | 0.781 | 0.096 |
| Disorders of fluid, electrolyte and acid-base balance | 7076(47) | 2356(46.9) | 2373(47.3) | 0.711 | 0.939 |
| Shock | 649(4.3) | 212(4.2) | 215(4.3) | 0.986 | 0.792 |
| Respiratory failure | 5438(36.1) | 1847(36.8) | 1788(35.6) | 0.378 | 0.388 |
| Convulsions | 1050(7.0) | 372(7.4) | 354(7.1) | 0.939 | 0.296 |
| Muscle spasm | 78(0.5) | 31(0.6) | 23(0.5) | 0.458 | 0.406 |
| Pulmonary infection | 1442(9.6) | 500(10) | 485(9.7) | 0.980 | 0.426 |
| Urinary tract infection | 1749(11.6) | 624(12.4) | 592(11.8) | 0.958 | 0.122 |
| Intracranial infection | 353(2.3) | 101(2) | 116(2.3) | 0.833 | 0.170 |
| Sepsis | 743(4.9) | 274(5.5) | 240(4.8) | 0.408 | 0.143 |
| Cerebral edema | 4152(27.6) | 1389(27.7) | 1399(27.9) | 0.702 | 0.897 |
| Hydrocephalus | 5447(36.2) | 1844(36.7) | 1842(36.7) | 0.619 | 0.477 |
| Nausea and vomiting | 400(2.7) | 132(2.6) | 124(2.5) | 0.475 | 0.918 |
| Headache | 5712(37.9) | 1931(38.5) | 1920(38.3) | 0.816 | 0.503 |
| Anemia | 3293(21.9) | 1115(22.2) | 1085(21.6) | 0.600 | 0.613 |
| Gastro-esophageal reflux | 1209(8.0) | 407(8.1) | 403(8.0) | 0.961 | 0.860 |
| Dysphagia | 1703(11.3) | 572(11.4) | 561(11.2) | 0.755 | 0.871 |
| Aphasia | 1282(8.5) | 428(8.5) | 462(9.2) | 0.122 | 0.980 |
| Nontraumatic intracerebral hemorrhage | 3662(24.3) | 1225(24.4) | 1241(24.7) | 0.575 | 0.907 |
| Elevated white blood cell count | 1519(10.1) | 486(9.7) | 495(9.9) | 0.790 | 0.405 |
| Thrombocytopenia | 616(4.1) | 210(4.2) | 210(4.2) | 0.826 | 0.776 |
| Facial weakness | 856(5.7) | 277(5.5) | 274(5.5) | 0.610 | 0.657 |
| Embolism and thrombosis of deep veins of lower extremity | 431(2.9) | 131(2.6) | 131(2.6) | 0.463 | 0.347 |
| Cerebral aneurysm, no ruptured | 1399(9.3) | 506(10.1) | 445(8.9) | 0.175 | 0.099 |
| Cerebrovascular arteriovenous malformation | 181(1.2) | 60(1.2) | 53(1.1) | 0.394 | 0.969 |
| Disordered phosphorus metabolism | 1948(12.9) | 676(13.5) | 611(12.2) | 0.089 | 0.336 |
| Disordered magnesium metabolism | 934(6.2) | 278(5.5) | 289(5.8) | 0.455 | 0.087 |
| Cerebral vasospasm and vasoconstriction | 2899(19.3) | 975(19.4) | 912(18.2) | 0.069 | 0.793 |
| Constipation | 978(6.5) | 320(6.4) | 329(6.6) | 0.819 | 0.763 |
| Total number of diagnoses |  |  |  | 0.724 | 0.181 |
| Mean ± SD | 15.3±7 | 15.5±7.1 | 15.3±7 |  |  |
| Median (IQR) | 15(10,20) | 15(10,20) | 15(10,20) |  |  |
| **Procedures during hospitalization** | | | | | |
| Occlusion of intracranial artery | 2357(15.7) | 784(15.6) | 791(15.8) | 0.845 | 0.951 |
| Restriction of intracranial artery | 3275(21.8) | 1109(22.1) | 1078(21.5) | 0.578 | 0.613 |
| Excision of intracranial artery | 111(0.7) | 36(0.7) | 29(0.6) | 0.241 | 0.885 |
| Bypass operation of intracranial arteries | 23(0.2) | 9(0.2) | 12(0.2) | 0.228 | 0.683 |
| Monitoring of arterial pulse | 611(4.1) | 204(4.1) | 202(4.0) | 0.909 | 0.986 |
| Monitoring of arterial pressure | 1113(7.4) | 356(7.1) | 366(7.3) | 0.950 | 0.479 |
| Monitoring of central nervous electrical activity | 745(4.9) | 252(5) | 281(5.6) | 0.069 | 0.839 |
| Percutaneous ventriculostomy | 3649(24.2) | 1220(24.3) | 1219(24.3) | 0.963 | 0.922 |
| Airway intubation | 2990(19.9) | 1029(20.5) | 991(19.7) | 0.661 | 0.326 |
| Tracheostomy | 455(3.0) | 137(2.7) | 162(3.2) | 0.301 | 0.288 |
| Mechanical ventilation |  |  |  |  |  |
| Less than 24 consecutive hours | 1306(8.7) | 435(8.7) | 430(8.6) | 0.811 | 0.985 |
| 24-96 consecutive hours | 1976(13.1) | 608(12.1) | 626(12.5) | 0.447 | 0.064 |
| Greater than 96 consecutive hours | 2145(14.2) | 767(15.3) | 713(14.2) | 0.587 | 0.072 |
| Lumbar puncture | 690(4.6) | 266(5.3) | 254(5.1) | 0.378 | 0.039 |
| Insertion of feeding device into stomach | 1209(8) | 407(8.1) | 403(8) | 0.961 | 0.860 |
| Introduction of nutritional substance into upper GI | 539(3.6) | 191(3.8) | 208(4.1) | 0.090 | 0.461 |
| Insertion of monitoring device into upper artery | 1405(9.3) | 499(9.9) | 482(9.6) | 0.799 | 0.202 |
| Insertion of infusion device into superior vena cava | 2976(19.8) | 924(18.4) | 957(19.1) | 0.562 | 0.035 |
| Ultrasonography of superior vena cava | 545(3.6) | 176(3.5) | 193(3.8) | 0.391 | 0.708 |
| Fluoroscopy of artery | 6699(44.5) | 2173(43.3) | 2216(44.2) | 0.953 | 0.137 |
| Administration of thrombolytics and platelet inhibitors | 260(1.7) | 95(1.9) | 85(1.7) | 0.717 | 0.441 |
| Transfusion of blood and blood products | 812(5.4) | 260(5.2) | 298(5.9) | 0.096 | 0.560 |
| Total number of procedures |  |  |  | 0.772 | 0.336 |
| Mean ± SD | 5.5±5.2 | 5.6±5.3 | 5.6±5.3 |  |  |
| Median (IQR) | 4.0(1.0,8.0) | 4.0(2.0,8.0) | 4.0(2.0,8.0) |  |  |
| Continuous variables were presented as means [standard deviation (SD)] or medians [interquartile range (IQR)]. Categorical variables were presented as numbers (percentage).  P^#^: Comparison of parameters between the Development cohort and Test cohort.  P*: Comparison of parameters between the Training cohort and Validation cohort. | | | | | |

GI: gastrointestinal
