# Supplementary material for: Machine learning models for predicting extended length of stay and hospital charges in nontraumatic subarachnoid hemorrhage
Source: Front Neurol. 2026 Feb 4;17:1737503. doi: 10.3389/fneur.2026.1737503 (PMC12913072; doi:10.3389/fneur.2026.1737503)
Supplement: Supplementary file 4 [file Table_4.docx]

| **Supplementary table 4. Performance of the ML models for extended LOS prediction** | | | | | | | |
| --- | --- | --- | --- | --- | --- | --- | --- |
| Models | AUC | Sensitivity | Specificity | PPV | NPV | Accuracy | F1 score |
| LightGBM | 0.931 | 0.693 | 0.938 | 0.781 | 0.904 | 0.878 | 0.735 |
| Catboost | 0.931 | 0.680 | 0.944 | 0.796 | 0.901 | 0.879 | 0.734 |
| GBM | 0.930 | 0.680 | 0.943 | 0.794 | 0.901 | 0.879 | 0.733 |
| XGBoost | 0.929 | 0.683 | 0.941 | 0.790 | 0.902 | 0.878 | 0.733 |
| AdaBoost | 0.929 | 0.677 | 0.944 | 0.796 | 0.900 | 0.879 | 0.732 |
| RF | 0.928 | 0.667 | 0.947 | 0.803 | 0.898 | 0.879 | 0.729 |
| ET | 0.928 | 0.660 | 0.945 | 0.792 | 0.896 | 0.875 | 0.720 |
| LR | 0.927 | 0.671 | 0.942 | 0.790 | 0.899 | 0.876 | 0.726 |
| SVM | 0.925 | 0.656 | 0.949 | 0.806 | 0.895 | 0.878 | 0.724 |
| KNN | 0.921 | 0.607 | 0.958 | 0.822 | 0.883 | 0.872 | 0.698 |
| ANN | 0.910 | 0.668 | 0.930 | 0.755 | 0.897 | 0.866 | 0.709 |
| DT | 0.885 | 0.617 | 0.939 | 0.764 | 0.884 | 0.860 | 0.683 |
| AUC: the area under receiver operating characteristic curve; AdaBoost: adaptive boosting; ANN: artificial neural network; CatBoost: categorical boosting; DT: decision tree; ET: extra tree; FB: gradient boosting machine; GBM: gradient boosting machine; KNN: K-nearest neighbor; LightGBM: light gradient boosting machine; LR: logistic regression; ML: machine learning; NPV: negative predictive value; PPV: positive predictive value; RF: random forest; SVM: support vector machine; XGBoost: eXtreme gradient boosting. | | | | | | | |
