# Supplementary material for: Machine learning models for predicting extended length of stay and hospital charges in nontraumatic subarachnoid hemorrhage
Source: Front Neurol. 2026 Feb 4;17:1737503. doi: 10.3389/fneur.2026.1737503 (PMC12913072; doi:10.3389/fneur.2026.1737503)
Supplement: Supplementary file 5 [file Table_5.docx]

| **Supplementary table S5. AUC values of the top five ML models with increasing number of features ranked by importance for LOS prediction** | | | | | |
| --- | --- | --- | --- | --- | --- |
| Feature numbers | LightGBM | CatBoost | GBM | XGBoost | AdaBoost |
| 1 | 0.587 | 0.587 | 0.587 | 0.587 | 0.727 |
| 2 | 0.748 | 0.748 | 0.748 | 0.748 | 0.748 |
| 3 | 0.829 | 0.823 | 0.823 | 0.829 | 0.829 |
| 4 | 0.864 | 0.866 | 0.865 | 0.865 | 0.865 |
| 5 | 0.880 | 0.881 | 0.881 | 0.881 | 0.880 |
| 6 | 0.888 | 0.898 | 0.889 | 0.891 | 0.894 |
| 7 | 0.894 | 0.904 | 0.897 | 0.897 | 0.900 |
| 8 | 0.907 | 0.908 | 0.901 | 0.907 | 0.908 |
| 9 | 0.910 | 0.910 | 0.910 | 0.910 | 0.911 |
| 10 | 0.911 | 0.913 | 0.912 | 0.912 | 0.916 |
| 11 | 0.917 | 0.919 | 0.918 | 0.918 | 0.917 |
| 12 | 0.918 | 0.921 | 0.920 | 0.920 | 0.920 |
| 13 | 0.912 | 0.922 | 0.923 | 0.922 | 0.921 |
| 14 | 0.921 | 0.924 | 0.923 | 0.923 | 0.922 |
| 15 | 0.916 | 0.924 | 0.924 | 0.923 | 0.923 |
| 16 | 0.919 | 0.926 | 0.925 | 0.924 | 0.925 |
| 17 | 0.919 | 0.926 | 0.925 | 0.925 | 0.925 |
| 18 | 0.920 | 0.928 | 0.926 | 0.926 | 0.927 |
| 19 | 0.921 | 0.928 | 0.927 | 0.926 | 0.927 |
| 20 | 0.922 | 0.928 | 0.928 | 0.927 | 0.927 |
| 25 | 0.931 | 0.931 | 0.930 | 0.929 | 0.929 |
| AUC: the area under receiver operating characteristic curve; AdaBoost: adaptive boosting; CatBoost: categorical boosting; GBM: gradient boosting machine; LightGBM: light gradient boosting machine; LOS: length of stay; ML: machine learning; XGBoost: eXtreme gradient boosting. | | | | | |
