# Supplementary material for: Machine learning models for predicting extended length of stay and hospital charges in nontraumatic subarachnoid hemorrhage
Source: Front Neurol. 2026 Feb 4;17:1737503. doi: 10.3389/fneur.2026.1737503 (PMC12913072; doi:10.3389/fneur.2026.1737503)
Supplement: Supplementary file 6 [file Table_6.docx]

| **Supplementary table S6. Performance metrics of the CatBoost model with increasing number of features ranked by importance for extended LOS prediction** | | | | | | | |
| --- | --- | --- | --- | --- | --- | --- | --- |
| Feature numbers | AUC | Sensitivity | Specificity | PPV | NPV | Accuracy | F1 Score |
| 1 | 0.587 | 0.000 | 1.000 | 0.000 | 0.756 | 0.756 | 0.000 |
| 2 | 0.748 | 0.638 | 0.815 | 0.527 | 0.874 | 0.772 | 0.577 |
| 3 | 0.823 | 0.462 | 0.911 | 0.627 | 0.840 | 0.802 | 0.532 |
| 4 | 0.866 | 0.566 | 0.928 | 0.717 | 0.869 | 0.839 | 0.632 |
| 5 | 0.881 | 0.580 | 0.934 | 0.738 | 0.873 | 0.847 | 0.650 |
| 6 | 0.898 | 0.638 | 0.931 | 0.750 | 0.888 | 0.860 | 0.689 |
| 7 | 0.904 | 0.640 | 0.937 | 0.767 | 0.890 | 0.865 | 0.698 |
| 8 | 0.908 | 0.646 | 0.934 | 0.761 | 0.891 | 0.864 | 0.698 |
| 9 | 0.910 | 0.651 | 0.933 | 0.758 | 0.892 | 0.864 | 0.701 |
| 10 | 0.913 | 0.659 | 0.935 | 0.765 | 0.895 | 0.867 | 0.708 |
| 11 | 0.919 | 0.654 | 0.939 | 0.775 | 0.894 | 0.869 | 0.709 |
| 12 | 0.921 | 0.666 | 0.937 | 0.774 | 0.897 | 0.871 | 0.716 |
| 13 | 0.922 | 0.680 | 0.939 | 0.783 | 0.901 | 0.876 | 0.728 |
| 14 | 0.924 | 0.682 | 0.940 | 0.785 | 0.902 | 0.877 | 0.730 |
| 15 | 0.924 | 0.668 | 0.940 | 0.783 | 0.898 | 0.874 | 0.721 |
| 16 | 0.926 | 0.676 | 0.943 | 0.792 | 0.900 | 0.877 | 0.729 |
| 17 | 0.926 | 0.673 | 0.940 | 0.784 | 0.899 | 0.875 | 0.724 |
| 18 | 0.928 | 0.674 | 0.941 | 0.788 | 0.900 | 0.876 | 0.727 |
| 19 | 0.928 | 0.678 | 0.941 | 0.789 | 0.900 | 0.877 | 0.729 |
| 20 | 0.928 | 0.684 | 0.941 | 0.791 | 0.902 | 0.879 | 0.733 |
| 25 | 0.931 | 0.680 | 0.944 | 0.796 | 0.901 | 0.879 | 0.733 |
| AUC: the area under receiver operating characteristic curve; CatBoost: categorical boosting; LOS: length of stay; NPV: negative predictive value; PPV: positive predictive value. | | | | | | | |
