# Supplementary material for: Machine learning models for predicting extended length of stay and hospital charges in nontraumatic subarachnoid hemorrhage
Source: Front Neurol. 2026 Feb 4;17:1737503. doi: 10.3389/fneur.2026.1737503 (PMC12913072; doi:10.3389/fneur.2026.1737503)
Supplement: Supplementary file 7 [file Table_7.docx]

| **Supplementary table 7. Comparison of the 7-variable model (based on full hospitalization data) and the 25-early-hospitalization variable model in predicting extended LOS.** | | | | |
| --- | --- | --- | --- | --- |
| **Performance metric** | **7 variables model (validation)** | 7 variables model (test) | 25 variables model (validation) | 25 variables model(test) |
| **AUC** | 0.904 | 0.910 | 0.651 | 0.652 |
| **Sensitivity** | 0.640 | 0.620 | 0.001 | 0.001 |
| **Specificity** | 0.937 | 0.942 | 0.999 | 0.999 |
| **PPV** | 0.767 | 0.768 | 1.000 | 0.500 |
| **NPV** | 0.890 | 0.889 | 0.756 | 0.763 |
| **Accuracy** | 0.865 | 0.866 | 0.756 | 0.763 |
| **F1 Score** | 0.698 | 0.686 | 0.002 | 0.002 |
| AUC: the area under receiver operating characteristic curve; NPV: negative predictive value; PPV: positive predictive value; | | | | |
