# Supplementary material for: Machine learning models for predicting extended length of stay and hospital charges in nontraumatic subarachnoid hemorrhage
Source: Front Neurol. 2026 Feb 4;17:1737503. doi: 10.3389/fneur.2026.1737503 (PMC12913072; doi:10.3389/fneur.2026.1737503)
Supplement: Supplementary file 8 [file Table_8.docx]

| **Supplementary table 8. Demographic and clinical characteristics of patients with normal and high hospital charges based on extended LOS, and results of univariate and multivariate analyses** | | | | | | | |
| --- | --- | --- | --- | --- | --- | --- | --- |
| Variables (%) | Normal charge  (≤743018.5)  (n = 4490) | High charge  (>743018.5)  (n = 1497) | Univariate analysis |  | Multivariate analysis | | |
|  |  |  | P |  | B | OR(95%CI) | P |
| **Patient Demographics** |  |  |  |  |  |  |  |
| Age (years) |  |  | **0.046** |  | -0.012 | 0.988(0.981-0.994) | **＜0.001** |
| Mean ± SD | 57.5±14.0 | 54.9±14.7 |  |  |  |  |  |
| Median (IQR) | 58.0(49.0,67.0) | 57.0(46.0,65.0) |  |  |  |  |  |
| Gender (%) |  |  | 0.447 |  |  |  |  |
| Females | 1559(34.7) | 536(35.8) |  |  |  |  |  |
| Males | 2931(65.3) | 961(64.2) |  |  |  |  |  |
| Race (%) |  |  | **<0.001** |  |  |  |  |
| White | 2553(56.9) | 714(47.7) |  |  |  | 1.000 | **0.010** |
| Black | 898(20.0) | 261(17.4) |  |  | 0.119 | 1.126(0.918-1.381) | 0.254 |
| Hispanic | 563(12.5) | 325(21.7) |  |  | 0.345 | 1.411(1.148-1.735) | **0.001** |
| Other | 476(10.6) | 197(13.2) |  |  | 0.023 | 1.024(0.815-1.285) | 0.840 |
| Length of stay (days) |  |  | **<0.001** |  | - | - | - |
| Mean ± SD | 25.9±10.4 | 38.6±28.6 |  |  |  |  |  |
| Median (IQR) | 23.0(20.0,28.0) | 30.0(23.0,42.0) |  |  |  |  |  |
| Total charges (dollars) |  |  | **<0.001** |  | - | - | - |
| Mean ± SD | 414238.2±164250.1 | 1150711.8±453106.7 |  |  |  |  |  |
| Median (IQR) | 404266.5(288665.3, 539692.3) | 1012407.0(850759.0, 1290173.0) |  |  |  |  |  |
| Median household income quartile (%) | | | **<0.001** |  |  |  |  |
| 0–25th | 1444(32.2) | 429(28.7) |  |  |  | 1.000 | **0.035** |
| 26–50th | 1160(25.8) | 353(23.6) |  |  | 0.109 | 1.115(0.916-1.357) | 0.278 |
| 51–75th | 1062(23.7) | 359(24.0) |  |  | 0.039 | 1.040(0.852-1.269) | 0.700 |
| 76–100th | 824(18.4) | 356(23.8) |  |  | 0.296 | 1.345(1.088-1.663) | **0.006** |
| Primary expected payer (%) |  |  | **<0.001** |  |  |  |  |
| Medicare | 1458(32.5) | 389(26.0) |  |  |  | 1.000 | 0.658 |
| Medicaid | 944(21.0) | 415(27.7) |  |  | 0.152 | 1.164(0.918-1.477) | 0.210 |
| Private insurance | 1641(36.5) | 546(36.5) |  |  | 0.084 | 1.088(0.883-1.341) | 0.430 |
| Other | 447(10.0) | 147(9.8) |  |  | 0.066 | 1.069(0.794-1.437) | 0.661 |
| Non-elective admission | 4342(96.7) | 1446(96.6) | 0.836 |  |  |  |  |
| Hospitalization year (%) |  |  |  |  |  |  |  |
| 2016 | 960(21.4) | 243(16.2) |  |  |  |  |  |
| 2017 | 940(20.9) | 257(17.2) |  |  |  |  |  |
| 2018 | 871(19.4) | 335(22.4) |  |  |  |  |  |
| 2019 | 901(20.1) | 343(22.9) |  |  |  |  |  |
| 2020 | 818(18.2) | 319(21.3) |  |  |  |  |  |
| Hospitalization season (%) |  |  | 0.725 |  |  |  |  |
| Spring (March-May) | 1087(24.2) | 346(23.1) |  |  |  |  |  |
| Summer (June-August) | 1103(24.6) | 362(24.2) |  |  |  |  |  |
| Fall (September-November) | 1159(25.8) | 405(27.1) |  |  |  |  |  |
| Winter (December-February) | 1141(25.4) | 384(25.7) |  |  |  |  |  |
| Hospitalization on weekends (%) | 1246(27.8) | 431(28.8) | 0.438 |  |  |  |  |
| Hospital admission transfer indicator (%) | | | 0.205 |  |  |  |  |
| Not transferred/Standard admission | 2153(48.0) | 773(51.6) |  |  |  |  |  |
| From acute care hospital | 2163(48.2) | 660(44.1) |  |  |  |  |  |
| From other facility | 174(3.9) | 64(4.3) |  |  |  |  |  |
| Hospital discharge transfer indicator (%) |  |  | **<0.001** |  |  |  |  |
| Not transferred | 1587(35.3) | 387(25.9) |  |  |  | 1.000 | **0.021** |
| To acute care hospital | 93(2.1) | 63(4.2) |  |  | 0.350 | 1.419(0.924-2.178) | 0.109 |
| To other facility | 2810(62.6) | 1047(69.9) |  |  | 0.272 | 1.313(1.077-1.600) | **0.007** |
| Died during hospitalization | 192(4.3) | 103(6.9) | **<0.001** |  | 0.286 | 1.332(0.937-1.892) | 0.110 |
| **Hospital demographics** (%) | | | | | | | |
| Hospital region |  |  | **0.004** |  |  |  |  |
| Northeast | 854(19.0) | 318(21.2) |  |  |  | 1.000 | **＜0.001** |
| Midwest | 947(21.1) | 129(8.6) |  |  | -1.438 | 0.238(0.182-0.310) | **＜0.001** |
| South | 1882(41.9) | 476(31.8) |  |  | -0.821 | 0.440(0.358-0.541) | **＜0.001** |
| West | 807(18.0) | 574(38.3) |  |  | 0.445 | 1.561(1.269-1.919) | **＜0.001** |
| Hospital bed size |  |  | **0.003** |  |  |  |  |
| Small | 196(4.4) | 88(5.9) |  |  |  | 1.000 | 0.926 |
| Medium | 743(16.5) | 285(19.0) |  |  | -0.017 | 0.983(0.689-1.404) | 0.926 |
| Large | 3551(79.1) | 1124(75.1) |  |  | 0.021 | 1.021(0.736-1.418) | 0.901 |
| Hospital location/teaching status |  |  | 0.081 |  |  |  |  |
| Rural | 27(0.6) | 0(0.0) |  |  |  |  |  |
| Urban nonteaching | 236(5.3) | 108(7.2) |  |  |  |  |  |
| Urban teaching | 4227(94.1) | 1389(92.8) |  |  |  |  |  |
| Hospital control/ownership (%) |  |  | **<0.001** |  |  |  |  |
| Government, nonfederal | 786(17.5) | 160(10.7) |  |  |  | 1.000 | **＜0.001** |
| Private, not-profit | 3436(76.5) | 970(64.8) |  |  | 0.572 | 1.772(1.425-2.203) | **＜0.001** |
| Private, invest-own | 268(6.0) | 367(24.5) |  |  | 2.743 | 15.535(11.566-20.866) | **＜0.001** |
| **Diagnosis, symptoms and complications on admission and during hospitalization** (%) | | | | | | | |
| Hypertension | 3244(72.2) | 1019(68.1) | **0.002** |  | -0.097 | 0.908(0.773-1.067) | 0.240 |
| Type Ⅱ diabetes | 751(16.7) | 242(16.2) | 0.614 |  |  |  |  |
| Coronary heart disease | 662(14.7) | 232(15.5) | 0.479 |  |  |  |  |
| Atrial fibrillation | 420(9.4) | 144(9.6) | 0.761 |  |  |  |  |
| Hyperlipidemia | 1218(27.1) | 317(21.2) | **<0.001** |  | -0.205 | 0.815(0.679-0.977) | **0.027** |
| Elevated blood glucose level | 537(12.0) | 179(12.0) | 0.998 |  |  |  |  |
| Chronic obstructive pulmonary disease | 375(8.4) | 98(6.5) | **0.025** |  | -0.261 | 0.770(0.578-1.027) | 0.075 |
| Hypothyroidism | 349(7.8) | 86(5.7) | **0.009** |  | -0.096 | 0.909(0.676-1.221) | 0.525 |
| Anxiety | 449(10.0) | 112(7.5) | **0.004** |  | -0.191 | 0.826(0.629-1.086) | 0.171 |
| Depression | 443(9.9) | 118(7.9) | **0.023** |  | -0.073 | 0.930(0.710-1.218) | 0.597 |
| Overweight and obesity | 635(14.1) | 221(14.8) | 0.553 |  |  |  |  |
| Tobacco use | 1625(36.2) | 439(29.3) | **<0.001** |  | 0.002 | 1.002(0.852-1.178) | 0.982 |
| Alcohol abuse | 339(7.6) | 111(7.4) | 0.863 |  |  |  |  |
| History of transient ischemic attack and cerebral infarction | 149(3.3) | 54(3.6) | 0.593 |  |  |  |  |
| Long term (current) use of anticoagulants and antithrombotic/antiplatelets | 204(4.5) | 49(3.3) | **0.034** |  | -0.199 | 0.819(0.558-1.203) | 0.309 |
| Long term (current) use of aspirin | 410(9.1) | 94(6.3) | **0.001** |  | -0.172 | 0.842(0.632-1.123) | 0.242 |
| Contact with and (suspected) exposure to communicable diseases | 228(5.1) | 96(6.4) | **0.048** |  | 0.286 | 1.331(0.981-1.807) | 0.067 |
| Kidney failure | 26(0.6) | 15(1.0) | 0.086 |  |  |  |  |
| Hepatic failure | 749(16.7) | 334(22.3) | **<0.001** |  | 0.104 | 1.110(0.916-1.345) | 0.287 |
| Paralytic | 69(1.5) | 18(1.2) | 0.525 |  |  |  |  |
| Disorders of fluid, electrolyte and acid-base balance | 3232(72.0) | 1142(76.3) | **<0.001** |  | 0.025 | 1.026(0.865-1.215) | 0.771 |
| Shock | 184(4.1) | 111(7.4) | **<0.001** |  | 0.082 | 1.086(0.806-1.462) | 0.588 |
| Respiratory failure | 2520(56.1) | 1190(79.5) | **<0.001** |  | 0.479 | 1.615(1.329-1.963) | **＜0.001** |
| Convulsions | 403(9.0) | 163(10.9) | **0.028** |  | -0.090 | 0.914(0.724-1.154) | 0.449 |
| Muscle spasm | 31(0.7) | 4(0.3) | 0.663 |  |  |  |  |
| Pulmonary infection | 977(21.8) | 562(37.5) | **<0.001** |  | 0.185 | 1.203(1.020-1.419) | **0.029** |
| Urinary tract infection | 1099(24.5) | 382(25.5) | 0.419 |  |  |  |  |
| Intracranial infection | 268(6.0) | 125(8.4) | **0.001** |  | 0.248 | 1.282(0.971-1.692) | 0.080 |
| Sepsis | 417(9.3) | 289(19.3) | **<0.001** |  | 0.411 | 1.508(1.223-1.860) | **＜0.001** |
| Cerebral edema | 1733(38.6) | 753(50.3) | **<0.001** |  | 0.129 | 1.137(0.978-1.322) | 0.094 |
| Hydrocephalus | 3066(68.3) | 1155(77.2) | **<0.001** |  | -0.253 | 0.776(0.227-2.661) | 0.687 |
| Nausea and vomiting | 106(2.4) | 22(1.5) | **0.039** |  | -0.24 | 0.787(0.420-1.474) | 0.454 |
| Headache | 3096(69.0) | 1160(77.5) | **<0.001** |  | 0.610 | 1.840(0.532-6.360) | 0.335 |
| Anemia | 1585(35.3) | 665(44.4) | **<0.001** |  | 0.241 | 1.273(1.095-1.479) | **0.002** |
| Gastro-esophageal reflux | 1058(23.6) | 565(37.7) | **<0.001** |  | -0.008 | 0.992(0.831-1.185) | 0.933 |
| Dysphagia | 1244(27.7) | 467(31.2) | **0.010** |  | -0.091 | 0.913(0.773-1.077) | 0.280 |
| Aphasia | 645(14.4) | 223(14.9) | 0.613 |  |  |  |  |
| Nontraumatic intracerebral hemorrhage | 1354(30.2) | 539(36.0) | **<0.001** |  | -0.091 | 0.913(0.773-1.077) | 0.280 |
| Elevated white blood cell count | 542(12.1) | 151(10.1) | **0.038** |  | -0.091 | 0.913(0.773-1.077) | 0.280 |
| Thrombocytopenia | 222(4.9) | 95(6.3) | **0.036** |  | -0.091 | 0.913(0.773-1.077) | 0.280 |
| Facial weakness | 331(7.4) | 91(6.1) | 0.091 |  |  |  |  |
| Embolism and thrombosis of deep veins of lower extremity | 283(6.3) | 168(11.2) | **<0.001** |  | 0.374 | 1.453(1.076-1.964) | **0.015** |
| Cerebral aneurysm, no ruptured | 575(12.8) | 204(13.6) | 0.414 |  |  |  |  |
| Cerebrovascular arteriovenous malformation | 68(1.5) | 31(2.1) | 0.144 |  |  |  |  |
| Disordered phosphorus metabolism | 882(19.6) | 373(24.9) | **<0.001** |  | 0.146 | 1.157(0.939-1.425) | 0.171 |
| Disordered magnesium metabolism | 489(10.9) | 106(7.1) | **<0.001** |  | -0.552 | 0.576(0.441-0.752) | **＜0.001** |
| Cerebral vasospasm and vasoconstriction | 1817(40.5) | 766(51.2) | **<0.001** |  | 0.364 | 1.438(1.235-1.675) | **＜0.001** |
| Constipation | 384(8.6) | 101(6.7) | **0.027** |  | 0.009 | 1.009(0.767-1.327) | 0.951 |
| Total number of diagnoses |  |  | **0.026** |  | 0.025 | 1.025(1.009-1.041) | **0.002** |
| Mean ± SD | 19.5±6.5 | 21.6±6.1 |  |  |  |  |  |
| Median (IQR) | 19.0(15.0,25.0) | 22.0(18.0,25.0) |  |  |  |  |  |
| **Procedures during hospitalization**(%) | | | | | | | |
| Occlusion of intracranial artery | 1247(27.8) | 458(30.6) | **0.036** |  | 0.234 | 1.264(1.055-1.514) | **0.011** |
| Restriction of intracranial artery | 1771(39.4) | 648(43.3) | **0.009** |  | 0.308 | 1.360(1.150-1.610) | **＜0.001** |
| Excision of intracranial artery | 53(1.2) | 27(1.8) | 0.069 |  |  |  |  |
| Bypass operation of intracranial arteries | 12(0.3) | 16(1.1) | **<0.001** |  | 0.904 | 2.469(0.981-6.215) | 0.055 |
| Monitoring of arterial pulse | 293(6.5) | 72(4.8) | **0.016** |  | -0.172 | 0.842(0.544-1.302) | 0.439 |
| Monitoring of arterial pressure | 550(12.2) | 129(8.6) | **<0.001** |  | -0.393 | 0.675(0.486-0.938) | **0.019** |
| Monitoring of central nervous electrical activity | 474(10.6) | 173(11.6) | 0.281 |  |  |  |  |
| Percutaneous ventriculostomy | 2352(52.4) | 858(57.3) | **0.001** |  | -0.074 | 0.929(0.790-1.091) | 0.369 |
| Airway intubation | 1379(30.7) | 655(43.8) | **<0.001** |  | 0.153 | 1.165(0.983-1.382) | 0.079 |
| Tracheostomy | 355(7.9) | 330(22.0) | **<0.001** |  | 0.687 | 1.987(1.603-2.462) | **＜0.001** |
| Mechanical ventilation |  |  |  |  |  |  |  |
| Less than 24 consecutive hours | 259(5.8) | 54(3.6) | **0.001** |  | -0.528 | 0.590(0.411-0.847) | **0.004** |
| 24-96 consecutive hours | 761(16.9) | 217(14.5) | **0.026** |  | -0.310 | 0.734(0.588-0.915) | **0.006** |
| Greater than 96 consecutive hours | 1422(31.7) | 865(57.8) | **<0.001** |  | 0.096 | 1.100(0.901-1.343) | 0.348 |
| Lumbar puncture | 318(7.1) | 82(5.5) | **0.031** |  | -0.444 | 0.641(0.474-0.868) | **0.004** |
| Insertion of feeding device into stomach | 1649(36.7) | 565(37.7) | 0.236 |  |  |  |  |
| Introduction of nutritional substance into upper GI | 473(10.5) | 159(10.6) | 0.925 |  |  |  |  |
| Insertion of monitoring device into upper artery | 701(15.6) | 259(17.3) | 0.123 |  |  |  |  |
| Insertion of infusion device into superior vena cava | 1649(36.7) | 592(39.5) | 0.051 |  |  |  |  |
| Ultrasonography of superior vena cava | 310(6.9) | 117(7.8) | 0.235 |  |  |  |  |
| Fluoroscopy of artery | 2616(58.3) | 805(53.8) | **0.002** |  | -0.463 | 0.629(0.539-0.735) | ＜0.001 |
| Administration of thrombolytics and platelet inhibitors | 146(3.3) | 51(3.4) | 0.771 |  |  |  |  |
| Transfusion of blood and blood products | 419(9.3) | 140(9.4) | 0.981 |  |  |  |  |
| Total number of procedures |  |  | **<0.001** |  | 0.085 | 1.089(1.074-1.104) | ＜0.001 |
| Mean ± SD | 9.6±5.7 | 13.2±6.7 |  |  |  |  |  |
| Median (IQR) | 9.0(6.0,13.0) | 13.0(7.0,18.0) |  |  |  |  |  |
| Continuous variables were presented as means [standard deviation (SD)] or medians [interquartile range (IQR)]. Categorical variables were presented as numbers (percentage). Bold values indicate statistically significant differences (P < 0.05).  GI: gastrointestinal; LOS: length of stay. | | | | | | | |
