# Supplementary material for: Machine learning models for predicting extended length of stay and hospital charges in nontraumatic subarachnoid hemorrhage
Source: Front Neurol. 2026 Feb 4;17:1737503. doi: 10.3389/fneur.2026.1737503 (PMC12913072; doi:10.3389/fneur.2026.1737503)
Supplement: Supplementary file 9 [file Presentation_1.pdf]

Supplementary Material 1

MATERIALS AND METHODS

Data source and patient population

Our study utilized data from the National Inpatient Sample (NIS) database, which is part of the Healthcare Cost and Utilization Project (HCUP) in the United States. The NIS is one of the largest publicly available all-payer inpatient healthcare databases, capturing approximately 7 to 8 million hospital admissions annually (http://www.hcup-us.ahrq.gov/overview.jsp). For the derivation and validation of predictive models, we conducted a retrospective cohort study using the NIS database, focusing on adult patients (aged 18 years and older) with a primary diagnosis of Nontraumatic subarachnoid hemorrhage (SAH) between 2016 and 2020. Patients were identified via the International Classification of Diseases, Tenth Revision, Clinical Modification (ICD-10-CM) diagnosis code I60 for SAH. To ensure that our study had broad generalizability and robustness, we included all adult patients with a primary diagnosis of SAH without excluding any cases. In the HCUP inpatient database, the first listed diagnosis is considered the primary diagnosis, defined as the condition established after the study to be chiefly responsible for occasioning the admission of the patient to the hospital for care. Patients were dichotomized on the basis of whether they had an extended postoperative hospital length of stay (LOS) , defined as an LOS greater than the 75th percentile for the entire cohort(1), which in this study was greater than 17 days. Clinically, a stay exceeding this upper-quartile threshold often signifies a severe disease course, including major complications (e.g., symptomatic vasospasm requiring intervention, shunt-dependent hydrocephalus), extended intensive care needs, or complex discharge planning(2, 3). Identifying this patient subgroup early is crucial for proactive resource allocation and tailored clinical management(4). All analyses involving the NIS database were conducted following the completion of the required training and certification for the proper use of the HCUP database (Certificate ID: HCUP-873CVV79I).

Data collection, processing, and cohort division

The variables considered in our study included age, gender, race (White, Black, Hispanic, Other), median household income quartile (0-25th, 26-50th, 51-75th, 76-100th percentiles), primary expected payer (Medicare, Medicaid, Private Insurance,

Other), hospital region (Northeast, Midwest, South, West), hospital bed size (small, medium, large), hospital teaching status (rural, urban nonteaching, urban teaching), hospital ownership (government, nonfederal, private not-for-profit, private for-profit), admission type (elective, non-elective), hospitalization season (spring [March-May], summer [June-August], fall [September-November], Winter [December-February]), weekend admissions (yes, no), hospital admission transfer indicators (not transferred/standard admission, from acute care hospital, from other facility), hospital discharge transfer indicators (not transferred, to acute care hospital, to other facility), LOS, total charges, and in-hospital mortality.

In addition to the abovementioned variables, we listed all diagnoses and procedures for each patient, calculated their frequency across the entire patient population, and selected those diagnoses and procedures that accounted for more than 5% and 3% of the patient population, respectively. Additionally, on the basis of clinical experience and understanding of the disease, we included certain variables that fell below these thresholds to ensure a comprehensive analysis. Diagnoses were identified via the ICD-10-CM codes, and procedures were identified via the ICD-10 Procedure Coding System (ICD-10-PCS). The codes corresponding to the collected diagnoses and procedures are provided in Supplementary Table 1.

The collected data on comorbidities and complications present at admission and during hospitalization included hypertension, type II diabetes, coronary heart disease, atrial fibrillation, hyperlipidemia, elevated blood glucose level, chronic obstructive pulmonary disease, hypothyroidism, anxiety, depression, overweight and obesity, tobacco use, alcohol abuse, history of transient ischemic attack and cerebral infarction, long-term (current) use of anticoagulants and antithrombotic/antiplatelets, long-term (current) use of aspirin, contact with and (suspected) exposure to communicable diseases, kidney failure, hepatic failure, paralytic disorders, fluid, electrolyte, and acid-base imbalance, shock, respiratory failure, convulsions, muscle spasm, pulmonary infection, urinary tract infection, intracranial infection, sepsis, cerebral edema, hydrocephalus, nausea and vomiting, headache, anemia, gastro-esophageal reflux, dysphagia, aphasia, nontraumatic intracerebral hemorrhage, elevated white blood cell count, thrombocytopenia, facial weakness, embolism and thrombosis of deep veins of lower extremity, cerebral aneurysm (not ruptured), cerebrovascular arteriovenous malformation, disordered phosphorus metabolism, disordered

magnesium metabolism, cerebral vasospasm and vasoconstriction, and constipation. The procedures performed during hospitalization included occlusion, restriction, or excision of intracranial arteries; bypass operation of intracranial arteries; monitoring of arterial pulse; monitoring of arterial pressure; monitoring of central nervous electrical activity; percutaneous ventriculostomy; airway intubation; tracheostomy; mechanical ventilation (less than 24 consecutive hours, 24–96 consecutive hours, greater than 96 consecutive hours); lumbar puncture; insertion of a feeding device into the stomach; introduction of nutritional substance into upper GI; insertion of a monitoring device into the upper artery; insertion of an infusion device into the superior vena cava; ultrasonography of the superior vena cava; fluoroscopy of the artery; administration of thrombolytics and platelet inhibitors; and transfusion of blood and blood products.

Regarding the predictive utility of the model, while we recognize the value of very early prediction (e.g., within 48 hours of admission), the nature of the NIS database, which excels in capturing diagnoses and procedures across the entire hospitalization but lacks granular early physiological metrics (such as hemorrhage volume or early GCS score), guided our approach. We therefore included variables measured throughout the hospitalization to comprehensively capture the impact of evolving complications and clinical events—the primary drivers of extended LOS. This design positions the model as a robust tool for dynamic risk assessment during the hospital course, aiding in prognosis and resource planning once a patient's clinical trajectory becomes clearer.

The number of actual patients and that of missing patients corresponding to each variable are shown in Supplementary Table 2, which also presents the number and proportion of each variable within the actual patient population (with continuous variables displayed via descriptive statistical indicators). To ensure the completeness of the dataset and facilitate smooth analysis and modeling, we used the multiple imputation by chained equations method, specifically employing the Markov chain Monte Carlo (McMC) technique, to fill in the missing values of the variables(5). The imputation process was implemented using the mice R package, with 5 iterations and 5 imputed datasets to ensure the accuracy and robustness of the results.

In the cohort division process, we first split all patients into a development cohort and a test cohort at an 8:2 ratio. The patients in the development cohort were

subsequently further divided into a training cohort and a validation cohort at a 7.5:2.5 ratio. The training cohort was used to construct various machine learning (ML) models, whereas the validation cohort was utilized for validating and comparing these models. The test cohort was reserved for the final evaluation of the selected model(6).

Initial variable screening, model construction, and model comparison for predicting extended LOS

To identify the variables for constructing the extended LOS prediction model, we performed initial variable screening on the development cohort. This process This process involves several steps: first, univariate analysis was conducted to assess the relationship between each variable and extended LOS. For continuous variables, we used the independent samples t-test (or Mann-Whitney U test for non-normally distributed variables), while for categorical variables, we applied the chi-square test (or Fisher's exact test when expected frequencies were low). Next, Spearman correlation analysis was performed to evaluate the monotonic relationships between continuous variables, helping to identify highly correlated variables and reduce multicollinearity. We chose Spearman's correlation because it is a non-parametric test that measures the strength and direction of the monotonic relationship between two variables, without assuming a linear relationship or normal distribution. This is particularly useful when the data does not meet the assumptions of Pearson correlation, such as linearity or normality, ensuring a more robust analysis. Then, least absolute shrinkage and selection operator (LASSO) regression was used for variable selection, where a penalty is applied to the regression coefficients to shrink less important variables toward zero, thereby excluding them from the model. The optimal penalty parameter (λ) was determined using 10-fold cross-validation, and the final λ value was selected based on the minimum standard error (λ - 1SE) approach, which helps to avoid overfitting while retaining essential variables. Finally, recursive feature elimination (RFE) was employed to iteratively remove the least important variables, further refining the feature set. Through these steps, we selected a set of variables that were most predictive of extended LOS while minimizing overfitting(7, 8).

Using the initial variables identified through our screening process, we trained and compared various ML models for predicting extended LOS. The training cohort was utilized to construct 12 different models: Random Forest (RF), Support Vector Machine (SVM), Gradient Boosting Machine (GBM), Adaptive Boosting (AdaBoost),

Artificial Neural Network (ANN), Decision Tree (DT), Extra Tree (ET), K-Nearest Neighbor (KNN), Light Gradient Boosting Machine (LightGBM), Logistic Regression (LR), Categorical Boosting (CatBoost), and eXtreme Gradient Boosting (XGBoost). A detailed description of these machine learning models can be found in Supplementary Material 2. A grid search combined with manual fine-tuning was applied to optimize the hyperparameters for each model(9).

The validation cohort was used to evaluate and compare the performance of these models. We employed a suite of complementary metrics, each addressing a different aspect of predictive performance, to establish a comprehensive comparison standard. The area under the receiver operating characteristic curve (AUC) served as our primary metric for overall model discriminative ability. We then examined operating characteristics at the optimal probability threshold (maximizing Youden's index): sensitivity and specificity to assess the trade-off in correctly identifying patients with and without extended LOS, and the positive and negative predictive values (PPV, NPV) to understand the clinical consequence of predictions in our cohort's context. Finally, the F1 score provided a balanced measure of precision and recall, which is particularly informative for imbalanced outcomes. This multi-faceted approach allowed us to select the most effective model not merely based on a single high AUC, but by ensuring a balanced and clinically interpretable performance profile across all relevant metrics for the clinical prediction task at hand(10).

Feature selection, model validation and model explanation for predicting extended LOS

ML model interpretation can be challenging. To address this difficulty, we employed the SHapley Additive exPlanations (SHAP) method, which ranks the importance of input features and explains prediction results, helping to overcome the "black-box" issue(11).

In our study, we aimed to construct a model that not only demonstrated excellent predictive performance but also remained clinically applicable by keeping the number of features manageable. The feature selection process began with the use of SHAP values to rank all the input features, which allowed us to systematically evaluate the impact of each feature on the model's predictions. We then incrementally inserted features into the prediction model on the basis of their importance, we started with the top 1 feature and added the top 2, top 3, and so on, up to the top 20 features. For each

set of included features, we assessed the model's performance via metrics such as the AUC. The final model was selected on the basis of its predictive ability and the number of features, ensuring a high AUC and other performance metrics with a minimal number of features.

To validate the performance of the final model, we used a validation cohort for internal validation and a test cohort for hold-out internal validation. A variety of techniques were used, including receiver operating characteristic (ROC) curves, precision–recall (P-R) curves, calibration curves, and decision curve analysis (DCA), to assess the performance of the model fully(10, 12).

To compare the predictive performance of the final model with the model using early hospitalization-related variables, we trained two CatBoost models using the same training set. The second model included early hospitalization-related variables, comprising a total of 25 variables: age, gender, race, median household income quartile, primary expected payer, hospital region, hospital bed size, hospital teaching status, hospital ownership, admission type, hospitalization season, weekend admissions, hospital admission transfer indicators, hypertension, type II diabetes, coronary heart disease, hyperlipidemia, hypothyroidism, anxiety, depression, overweight and obesity, tobacco use, alcohol abuse and history of transient ischemic attack and cerebral infarction. Both models were evaluated using the same validation and test cohorts, and their performance was compared using various metrics.

The SHAP method provides both global and local explanations(11). Global explanations included SHAP summary plots (the variable importance plots and the beeswarm plots) and SHAP dependence plots, which offered consistent and accurate attribution values for each feature and illustrated associations between the features and extended LOS. Local explanations, using waterfall plots and force plots, demonstrated how specific data inputs led to individual predictions, aiding in clinical decision-making by clarifying why a certain prediction was made for a specific patient. A representative interactive force plot for the validation cohort can be accessed at https://walkerdii.github.io/shap-force-plot/.

Web-based tool for predicting extended LOS using a streamlit framework

To facilitate the clinical use of our extended LOS prediction model, we implemented it in a user-friendly web application via the streamlit framework(13). This tool allowed healthcare providers to input patient data and quickly receive predictions

about the likelihood of extended LOS. By entering the relevant features from the final model, the application provides the probability of extended LOS and a force plot to explain the individual prediction, enhancing the interpretability and supporting clinical decision-making. This web application is publicly accessible at https://prediction-model-aodpzmcnilelv9yb3eabo5.streamlit.app.

Relationship between LOS and total charges and construction of a model for predicting total charges based on the LOS

To explore the relationship between LOS and total charges, we used scatter plots with regression lines, heatmaps, and violin plots. These visualizations highlighted trends and distributions, providing a comprehensive overview of the correlation between LOS and hospital charges.

Given that both LOS and total charges are continuous variables, we employed the decision tree method for predictive modeling. Decision trees are advantageous because of their interpretability, ability to handle numerical and categorical data, and capacity to capture nonlinear relationships without extensive preprocessing(14).

To determine the optimal hyperparameters for the decision tree, we performed hyperparameter tuning in two stages, using the negative mean squared error (NMSE) as the evaluation metric. First, to identify a suitable range for tree complexity, we evaluated models with maximum depths from 1 to 20 using 5-fold cross-validation on the training cohort. The depth yielding the highest cross-validated NMSE was selected. Subsequently, a more comprehensive grid search was conducted using GridSearchCV (with 5-fold cross-validation) to simultaneously optimize three key parameters: max_depth (4, 5, 6, 7, 8), min_samples_split (2, 5, 10), and min_samples_leaf (1, 2, 4). The combination of parameters that maximized the cross-validated NMSE was chosen as the final model configuration. This process ensured the selection of a model that balanced predictive performance and generalizability. We then constructed the decision tree model using the optimized hyperparameters and LOS data from the training cohort and validated its performance by plotting the distribution of prediction errors via both the validation and test cohorts, which further ensured the robustness and generalizability of our predictive model.

Factors associated with high hospital charges in patients with extended LOS

To identify factors contributing to high hospital charges among patients with extended LOS, we compared high-charge and normal-charge groups within the extended LOS

cohort, defined by the 75th percentile of total charges. Univariate analysis was first performed on all 90 variables, and statistically significant variables ($P < 0.05$) were included in a multivariate logistic regression model. To further refine the model and address multicollinearity, LASSO regression was applied, with the optimal penalty parameter (λ) determined through 10-fold cross-validation using the minimum standard error (λ - 1SE) approach. This process identified the final set of factors most strongly associated with high charges.

Statistical analysis

The data preparation, including data imputation, cohort division, and analysis, was conducted through SPSS (version 25.0, https://www.ibm.com/spss) and R (version 4.2.0, http://www.r-project.org). Continuous variables are presented herein as the means [standard deviations (SDs)] or medians [interquartile ranges (IQRs)] and were compared via the independent samples t test or the Mann–Whitney U test. Categorical variables are presented as numbers (percentages) and were compared via the chi-square test or Fisher's exact test.

For subsequent variable selection, relationship description, model construction, validation, interpretation, and clinical application, we primarily used Python (version 3.10.9, https://www.python.org), except for the DCA, which was performed using R. The AUC and P-R AUC were employed to evaluate the predictive power of the models. The AUC was calculated by plotting the true positive rate against the false positive rate at various threshold settings, whereas the P-R AUC was calculated by plotting the precision against the recall. The AUC values of two ROC curves were compared via the DeLong test, a nonparametric statistical method for comparing correlated ROC curves(15). A two-tailed P value < 0.05 was considered statistically significant, and a Hosmer–Lemeshow (HL) test P value > 0.05 indicated good model fit.

1. Collins TC, Daley J, Henderson WH, Khuri SF. Risk factors for prolonged length of stay after major elective surgery. Ann Surg. 1999;230(2):251-9.

2. Gershman B, Moreira DM, Boorjian SA, Lohse CM, Cheville JC, Costello BA, et al. Comprehensive Characterization of the Perioperative Morbidity of Cytoreductive Nephrectomy. Eur Urol. 2016;69(1):84-91.

3. Lai JC, Shui AM, Duarte-Rojo A, Ganger DR, Rahimi RS, Huang CY, et al. Frailty, mortality, and health care utilization after liver transplantation: From the Multicenter Functional Assessment in Liver Transplantation (FrAILT) Study. Hepatology. 2022;75(6):1471-9.
4. Krell RW, Girotti ME, Dimick JB. Extended length of stay after surgery: complications, inefficient practice, or sick patients? JAMA Surg. 2014;149(8):815-20.
5. van Buuren S, Groothuis-Oudshoorn K. mice: Multivariate Imputation by Chained Equations in R. Journal of Statistical Software. 2011;45(3):1 - 67.
6. Cabitza F, Campagner A, Soares F, García de Guadiana-Romualdo L, Challa F, Sulejmani A, et al. The importance of being external. methodological insights for the external validation of machine learning models in medicine. Computer methods and programs in biomedicine. 2021;208:106288.
7. Handorf E, Yin Y, Slifker M, Lynch S. Variable selection in social-environmental data: sparse regression and tree ensemble machine learning approaches. BMC Med Res Methodol. 2020;20(1):302.
8. Degenhardt F, Seifert S, Szymczak S. Evaluation of variable selection methods for random forests and omics data sets. Brief Bioinform. 2019;20(2):492-503.
9. Bischl B, Binder M, Lang M, Pielok T, Richter J, Coors S, et al. Hyperparameter optimization: Foundations, algorithms, best practices, and open challenges. WIREs: Data Mining & Knowledge Discovery. 2023;13(2):1-43.
10. Carrington AM, Manuel DG, Fieguth PW, Ramsay T, Osmani V, Wernly B, et al. Deep ROC Analysis and AUC as Balanced Average Accuracy, for Improved Classifier Selection, Audit and Explanation. IEEE Trans Pattern Anal Mach Intell. 2023;45(1):329-41.
11. Lundberg SM, Erion G, Chen H, DeGrave A, Prutkin JM, Nair B, et al. From Local Explanations to Global Understanding with Explainable AI for Trees. Nat Mach Intell. 2020;2(1):56-67.
12. Schetinin V, Jakaite L, Krzanowski W. Bayesian averaging over decision tree models: An application for estimating uncertainty in trauma severity scoring. Int J Med Inform. 2018;112:6-14.
13. Bedoya AD, Economou-Zavlanos NJ, Goldstein BA, Young A, Jelovsek JE, O'Brien C, et al. A framework for the oversight and local deployment of safe and high-quality prediction models. J Am Med Inform Assoc. 2022;29(9):1631-6.

14. Sagi O, Rokach L. Explainable decision forest: Transforming a decision forest into an interpretable tree. Information Fusion. 2020;61:124-38.
15. Demler OV, Pencina MJ, D'Agostino RB, Sr. Misuse of DeLong test to compare AUCs for nested models. Stat Med. 2012;31(23):2577-87.
