# Supplementary material for: Machine learning models for predicting extended length of stay and hospital charges in nontraumatic subarachnoid hemorrhage
Source: Front Neurol. 2026 Feb 4;17:1737503. doi: 10.3389/fneur.2026.1737503 (PMC12913072; doi:10.3389/fneur.2026.1737503)
Supplement: Supplementary file 10 [file Presentation_2.pdf]

Supplementary Material 2

Detailed Description of Machine Learning Models

1. Random Forest (RF)

1.1 Algorithm Overview

Random Forest (RF) is an ensemble learning method designed for both classification and regression tasks(1). It operates by constructing a multitude of decision trees during training. The fundamental principle behind its robustness is the combination of bagging (Bootstrap Aggregating) and random feature selection. Each tree is trained on a different bootstrap sample of the original dataset, and at each split within a tree, only a random subset of features is considered. This randomness decorrelates the individual trees, reducing overfitting. For a binary classification task (e.g., extended vs. normal LOS), the final prediction is obtained by majority voting across all trees.

1.2 Algorithm Formulation and Key Process

For a binary classification task, the Random Forest model aggregates predictions from *B* decision trees. The predicted probability $\hat{p}_{RF}$ for the positive class (e.g., extended LOS) for a sample x is the proportion of trees that predict the positive class:

$$\hat{p}_{RF}(\mathbf{x})=\frac{1}{B}\sum_{b=1}^{B} I(\,T_b(\mathbf{x})="Positive"\,)$$

Where T_b(x) is the class prediction from the *b*-th decision tree, and $I(\cdot)$ is an indicator function that returns 1 if the condition is true and 0 otherwise. The final class label is then determined by applying a threshold (e.g., 0.5) to $\hat{p}_{RF}$.

The training process involves two key steps:

1) **Bootstrap Sampling:** For each tree, a training subset is created by randomly drawing samples from the original training set with replacement.
2) **Feature Randomization:** When splitting a node, the algorithm selects a random subset of features and determines the best split only from within this subset.

1.3. Key Hyperparameters Optimized in This Study

In our implementation, the following core hyperparameters were tuned via grid search:

1) n_estimators: The number of decision trees in the forest.

2) max_depth: The maximum depth of each tree.
3) max_features: The number of features to consider for the best split.
4) min_samples_split: The minimum number of samples required to split an internal node.

2. Support Vector Machine (SVM)

2.1. Algorithm Overview

The Support Vector Machine (SVM) is a powerful supervised learning algorithm primarily used for classification tasks(2). Its core objective is to find an optimal hyperplane in a high-dimensional feature space that best separates data points of different classes. This optimal hyperplane is the one that achieves the maximum margin, defined as the distance between the hyperplane and the nearest data points from each class, known as support vectors. SVMs can handle both linearly separable and non-linearly separable data through the use of kernel functions, which implicitly map the original features into a higher-dimensional space where a linear separation becomes possible.

2.2. Algorithm Formulation and Key Process

For a binary classification task with training data $\{(\mathbf{x}_i,y_i)\}_{i=1}^{N}$ $where\ y_i \in \{-1,+1\}$, the standard linear SVM aims to find the hyperplane defined by $\mathbf{w}^T\mathbf{z}+b=0$. The decision function for a new sample $\mathbf{z}$ is:

$$f(\mathbf{z})=sign(\mathbf{w}^T\mathbf{z}+b)$$

To maximize the margin, SVM solves the following primal optimization problem (for the linearly separable case):

$$\min_{\mathbf{w},b} \ \frac{1}{2}\|\mathbf{w}\|^2$$

$$subject\ to \quad y_i(\mathbf{w}^T\mathbf{x}_i+b)\geq 1, \quad \forall i$$

In practice, data is often not perfectly separable. The *soft-margin* SVM introduces slack variables ξ_i to allow some misclassification, governed by a regularization parameter C:

$$\min_{\mathbf{w},b,\xi} \ \frac{1}{2}\|\mathbf{w}\|^2+C\sum_{i=1}^{N}\xi_i$$

$$subject\ to \quad y_i(\mathbf{w}^T\mathbf{x}_i+b)\geq 1-\xi_i, \quad \xi_i\geq 0, \quad \forall i$$

For non-linear problems, the kernel trick is applied. The data is mapped via a kernel

function $K(\mathbf{x}_i,\mathbf{x}_j)=\phi(\mathbf{x}_i)^T\phi(\mathbf{x}_j)$, and the decision function becomes:

$$f(\mathbf{z})=sign\left(\sum_{i=1}^{N}\alpha_i y_i K(\mathbf{x}_i,\mathbf{z})+b\right)$$

where α_i are Lagrange multipliers obtained from solving the dual optimization problem.

Key Process: The training involves solving the convex optimization problem to identify the support vectors ($\alpha_i > 0$) and the parameters defining the optimal hyperplane. Prediction for a new sample is based on which side of the hyperplane it lies.

2.3. Key Hyperparameters Optimized in This Study

In our implementation, the following core hyperparameters were tuned via grid search:

1) C: The regularization parameter. It controls the trade-off between achieving a large margin and minimizing classification error on the training data.
2) kernel: The kernel function type used (e.g., linear, radial basis function 'rbf', polynomial).
3) gamma: (For 'rbf' and 'poly' kernels) The kernel coefficient, which defines how far the influence of a single training example reaches.

3. Gradient Boosting Machine (GBM)

3.1. Algorithm Overview

Gradient Boosting Machine (GBM) is a powerful ensemble learning technique that builds predictive models in a stage-wise fashion, typically using decision trees as base learners(3). Unlike bagging methods (e.g., Random Forest) that train models independently, boosting methods like GBM train models sequentially. Each new model is trained to correct the errors made by the ensemble of all previous models. This is achieved by fitting the new model to the negative gradient (i.e., the residuals) of the loss function with respect to the current ensemble's predictions. GBM is highly effective for both regression and classification tasks and is known for its predictive accuracy.

3.2. Algorithm Formulation and Key Process

For a dataset with N samples $\{(\mathbf{x}_i,y_i)\}_{i=1}^{N}$, GBM constructs an additive model of M

base learners (e.g., decision trees) in a forward stage-wise manner:

$$F_M(\mathbf{x})=F_0(\mathbf{x})+v\sum_{m=1}^{M}\mathrm{h}_m(\mathbf{x})$$

where $F_0(\mathbf{x})$ is an initial guess (e.g., the mean response for regression or log-odds for classification), $\mathrm{h}_m(\mathbf{x})$ is the base learner (a decision tree) fitted at the m-th stage, and v is the *learning rate* or shrinkage parameter that controls the contribution of each tree.

At each stage m, for a given loss function $L(y, F)$ (e.g., squared error for regression, logistic loss for classification), the algorithm:

1) Computes the negative gradient (pseudo-residuals) r_{im} for each sample i:

$$r_{im}=-\left[\frac{\partial L(y_i, F(\mathbf{x}_i))}{\partial F(\mathbf{x}_i)}\right]_{F(\mathbf{x})=F_{m-1}(\mathbf{x})}$$

2) Fits a new base learner $\mathrm{h}_m(\mathbf{x})$ to these pseudo-residuals $\{r_{im}\}_{i=1}^{N}$.
3) Updates the ensemble model:

$$F_m(\mathbf{x})=F_{m-1}(\mathbf{x})+v\cdot\ \mathrm{h}_m(\mathbf{x})$$

The learning rate v scales the contribution of the new tree, providing regularization.

For binary classification (with labels $y_i \in \{0, 1\}$), the final output $F_M(\mathbf{x})$ is typically transformed via a logistic function to obtain the predicted probability:

$$\hat{p}(\mathbf{x})=\frac{1}{1+\exp(-F_M(\mathbf{x}))}$$

3.3. Key Hyperparameters Optimized in This Study

In our implementation, the following core hyperparameters were tuned via grid search:

1) n_estimators: The number of boosting stages (trees) to perform (M).
2) learning_rate (v): The shrinkage parameter that scales the contribution of each tree.
3) max_depth: The maximum depth of each individual decision tree base learner (h_m).
4) min_samples_split: The minimum number of samples required to split an internal node in a tree.
5) subsample: The fraction of samples to be used for fitting each individual base learner (stochastic gradient boosting).

4. Adaptive Boosting (AdaBoost)

4.1. Algorithm Overview

Adaptive Boosting (AdaBoost) is a seminal ensemble meta-algorithm primarily designed for binary classification(4). Its core idea is to combine multiple weak classifiers (e.g., shallow decision trees, often called "decision stumps") into a single strong classifier. AdaBoost operates sequentially. In each iteration, it trains a new weak classifier by placing higher emphasis on training samples that were misclassified by the ensemble of previous classifiers. This is achieved by maintaining and updating a weight for each training sample. Samples that are harder to classify correctly receive increasing weights, forcing subsequent weak learners to focus more on them. The final prediction is a weighted majority vote of all weak classifiers' predictions.

4.2. Algorithm Formulation and Key Process

Given a training set $\{(\mathbf{x}_i,y_i)\}_{i=1}^{N}$ *where* $y_i \in \{-1,+1\}$, AdaBoost proceeds for M rounds (or until a convergence criterion is met):

1) **Initialize weights**: $w_i^{(1)}=\frac{1}{N},\quad for\ i=1,2,\dots,N$
2) **For** $m = 1$ *to* M:

  a. **Train a weak classifier** $G_m(\mathbf{x})$using the current sample weights $\{w_i^{(m)}\}$.

  b. **Compute the weighted error** ϵ_m of $G_m(\mathbf{x}_i)$:

$$\epsilon_m=\frac{\sum_{i=1}^{N} w_i^{(m)}\cdot\ I(y_i\neq G_m(\mathbf{x}_i))}{\sum_{i=1}^{N} w_i^{(m)}}$$

  c. **Compute the classifier weight** α_m:

$$\alpha_m=\frac{1}{2}\ln\left(\frac{1-\epsilon_m}{\epsilon_m}\right)$$

This gives higher weight to more accurate classifiers.

  d. **Update the sample weights** for the next round:

$$w_i^{(m+1)}=w_i^{(m)}\cdot\ \exp\left(-\alpha_m\cdot\ y_i\cdot\ G_m(\mathbf{x}_i)\right)$$

This increases the weight of misclassified samples $y_i\neq G_m(\mathbf{x}_i)$ and decreases the weight of correctly classified ones.

  e. **Renormalize the weights** so that $\sum_{i=1}^{N} w_i^{(m)}=1$.

3) **Output the final strong classifier** $F(\mathbf{x})$:

$$F(\mathbf{x})=sign\left(\sum_{m=1}^{M} \alpha_m G_m(\mathbf{x})\right)$$

4.3. Key Hyperparameters Optimized in This Study

In our implementation, the following core hyperparameters were tuned via grid search:

1) n_estimators: The maximum number of weak classifiers (M) to train.
2) learning_rate: A multiplicative factor applied to the classifier weight α_m. Shrinking this weight provides regularization, making the learning process more conservative.

5. Artificial Neural Network (ANN)

5.1. Algorithm Overview

An Artificial Neural Network (ANN) is a computational model inspired by biological neural networks, designed to approximate complex, non-linear functions(5). It is a foundational algorithm in deep learning and is highly effective for both regression and classification tasks. An ANN consists of interconnected layers of processing units called neurons. Information flows from the input layer, through one or more hidden layers, to the output layer. Each connection has an associated weight, and each neuron applies a non-linear activation function to its weighted sum of inputs. The network learns by iteratively adjusting these weights to minimize the difference between its predictions and the true labels, a process typically performed via backpropagation and gradient-based optimization.

5.2. Algorithm Formulation and Key Process

Consider a feedforward ANN with L layers. Let the l-th layer contain n_l neurons, and let $\mathbf{z}^{(l)}$ and $\mathbf{a}^{(l)}$ denote its pre-activation and post-activation (output) vectors, respectively.

1) **Forward Propagation:** For a given input sample $\mathrm{x} = \mathbf{a}^{(0)}$, the information propagates forward layer by layer:

$$\mathbf{z}^{(l)} = \mathbf{W}^{(l)}\mathbf{a}^{(l\text{-}1)} + \mathbf{b}^{(l)}$$
$$\mathbf{a}^{(l)} = g^{(l)}(\mathbf{z}^{(l)})$$

where $\mathbf{W}^{(l)}$ is the weight matrix, $\mathbf{b}^{(l)}$ is the bias vector, and $g^{(l)}$ is the activation

function (e.g., *ReLU* for hidden layers, *sigmoid* for binary classification output). The final network output for a binary classification task is the scalar $a^{(L)}=\hat{p}(\mathbf{x})$, representing the predicted probability of the positive class.

2) **Learning via Backpropagation:** The network is trained by minimizing a loss function $J(\mathbf{W},\mathbf{b})$ (e.g., binary cross-entropy) over the training set. The gradients of the loss with respect to all parameters are calculated efficiently using the **backpropagation** algorithm, which applies the chain rule of calculus backward through the network:

$$\delta^{(L)}=\nabla_{\mathbf{a}^{(L)}}J\odot(g^{(L)})'(\mathbf{z}^{(L)})$$

$$\delta^{(l)}=((\mathbf{W}^{(l+1)})^T\delta^{(l+1)})\odot(g^{(l)})'(\mathbf{z}^{(l)}),\quad \textit{for } l=L\text{-}1,\ldots,1$$

$$\frac{\partial J}{\partial \mathbf{W}^{(l)}}=\delta^{(l)}(\mathbf{a}^{(l\text{-}1)})^T,\ \frac{\partial J}{\partial \mathbf{b}^{(l)}}=\delta^{(l)}$$

where $\odot$ denotes the element-wise (Hadamard) product. The weights are then updated iteratively via an optimization algorithm (e.g., Stochastic Gradient Descent, Adam) using these gradients.

5.3. Key Hyperparameters Optimized in This Study

In our implementation, the following core architectural and training hyperparameters were tuned via grid search:

1) Network Architecture: The number of hidden layers and the number of neurons per layer (defining the matrices $\mathbf{W}^{(l)}$).
2) Activation Function ($g^{(l)}$): The non-linear function used in hidden layers (e.g., ReLU, tanh).
3) Optimizer: The algorithm used for weight updates (e.g., Adam, SGD).
4) Learning Rate: The step size controlling the magnitude of weight updates during optimization.
5) Regularization: Techniques like L2 penalty (weight decay) or Dropout rate to prevent overfitting.

6. Decision Tree (DT)

6.1. Algorithm Overview

A Decision Tree (DT) is a non-parametric, hierarchical model used for both classification and regression tasks(6). It mimics a flowchart-like structure, where an internal node represents a test on a feature, a branch represents the outcome of the test,

and a leaf node holds a class label (for classification) or a continuous value (for regression). The model is constructed by recursively splitting the data into purer subsets based on the values of the input features. Decision Trees are highly interpretable due to their transparent, rule-based logic, and they form the foundational building block for many ensemble methods like Random Forests and Gradient Boosting.

6.2. Algorithm Formulation and Key Process (for Classification)

The construction of a classification tree is a recursive, top-down process that aims to partition the feature space into homogeneous regions. The core of the algorithm involves selecting the optimal split at each node. This is typically done by maximizing a measure of node purity. Two common impurity metrics are the Gini Index and Information Gain (based on Entropy).

1) **Gini Index:** For a node t containing a set of samples with class proportions p_k for $k = 1,...,K$, the Gini impurity is:

$$I_G(t)=1-\sum_{k=1}^{K} p_k^2$$

A perfectly pure node (all samples from one class) has a Gini index of 0.

2) **Information Gain:** The entropy of node t is defined as:

$$H(t)=-\sum_{k=1}^{K} p_k \log_2(p_k)$$

The *Information Gain* for a potential split s that divides node t into left and right child nodes t_L and t_R is the reduction in entropy:

$$IG(s,t)=H(t)-\left(\frac{N_L}{N}H(t_L)+\frac{N_R}{N}H(t_R)\right)$$

where N, N_L, and N_R are the sample counts in the parent, left, and right nodes, respectively. The split that maximizes $IG(s, t)$ is chosen.

Key Process: Starting from the root node containing the entire training set, the algorithm iteratively:

a. Searches over all features and possible split thresholds.
b. Selects the split that results in the greatest reduction in impurity (highest Information Gain or largest decrease in Gini Index).
c. Partitions the data according to the chosen split, creating child nodes.
d. Recursively applies steps 1-3 to each child node until a stopping criterion is met

(e.g., maximum depth is reached, node is pure, or node contains too few samples).

6.3. Key Hyperparameters Optimized in This Study

In our implementation, the following core hyperparameters were tuned via grid search to control tree growth and prevent overfitting:

1) max_depth: The maximum allowed depth of the tree.
2) min_samples_split: The minimum number of samples required to split an internal node.
3) min_samples_leaf: The minimum number of samples required to be at a leaf node.
4) max_features: The number of features to consider when looking for the best split.

7. Extra Tree (ET)

7.1. Algorithm Overview

Extra Trees (Extremely Randomized Trees) is an ensemble learning method that, like RF, builds multiple decision trees and aggregates their predictions(7). However, ET introduces an additional layer of randomness during tree construction to further reduce model variance and computational cost. While RF searches for the optimal split point for each candidate feature subset, ET selects the split points completely at random for each feature. This extreme randomization decorrelates the trees more strongly than RF, often leading to smoother decision boundaries and robustness against noisy features. ET is efficient and particularly effective for high-dimensional datasets.

7.2. Algorithm Formulation and Key Process

The algorithm for constructing an Extra Tree ensemble closely follows that of Random Forest but differs in the node splitting procedure.

Given a training set, the algorithm builds B decision trees. For each tree:

1) A bootstrap sample (or the entire training set) is used as the tree's growing data.
2) At each node, a random subset of K features is selected (similar to RF).
3) For each feature in this subset, a split value is chosen uniformly at random from the feature's range (observed in the node's samples), without calculating the optimal split based on impurity (e.g., Gini or MSE).
4) The best split among these randomly generated candidates is selected. This "best"

split is typically the one that yields the highest reduction in impurity, but the candidate pool is generated randomly.

The final prediction for a sample **x** in a regression task is the average of all tree predictions:

$$\hat{y}_{ET}(\mathbf{x})=\frac{1}{B}\sum_{b=1}^{B} T_b\,(\mathbf{x};\Theta_b)$$

For classification, it is the majority vote (mode) of the class predictions from all trees.

Key Difference from Random Forest: The core distinction lies in step 3. RF performs a (often exhaustive) search for the optimal split point within the feature subset, while ET picks split points randomly. This makes ET faster to train and can lead to better generalization in some cases by trading a small amount of bias for a larger reduction in variance.

7.3. Key Hyperparameters Optimized in This Study

In our implementation, the following core hyperparameters were tuned via grid search:

1) n_estimators: The number of trees in the ensemble (B).
2) max_depth: The maximum depth of each tree.
3) max_features (K): The size of the random subset of features to consider for splitting a node.
4) min_samples_split: The minimum number of samples required to split an internal node.

8. K-Nearest Neighbor (KNN)

8.1. Algorithm Overview

K-Nearest Neighbors (KNN) is a simple, instance-based, and non-parametric learning algorithm used for both classification and regression tasks(8). Unlike other models that construct a generalizing model from the training data, KNN memorizes the entire training dataset. Prediction for a new sample is made by examining the “k” training samples that are most similar (nearest) to it according to a predefined distance metric (e.g., Euclidean distance). For classification, the most common class among these “k” neighbors is assigned; for regression, the average of their target values is used. KNN's performance heavily depends on the choice of “k”, the distance metric, and the

scaling of the features.

8.2. Algorithm Formulation and Key Process

For a binary classification task, given a training set $\{(\mathbf{x}_i,y_i)\}_{i=1}^{N}$ and a new query point $\mathbf{x}_q$, the KNN algorithm proceeds as follows:

1) **Distance Calculation:** Compute the distance $d_p(\mathbf{x}_q,\mathbf{x}_i)$ between the query point and every training sample. A common choice is the **Minkowski distance**, a generalization that includes Euclidean and Manhattan distances as special cases:

$$d_p(\mathbf{x}_q,\mathbf{x}_i)=\left(\sum_{j=1}^{D}|x_{qj}-x_{ij}|^p\right)^{1/p}$$

where D is the number of features, and p is a parameter. For $p = 2$, this is the **Euclidean distance**; for $p = 1$, it is the **Manhattan distance**.

2) **Neighbor Identification:** Identify the set $N_k(\mathbf{x}_q)$ containing the indices of the "k" training points with the smallest distances to $\mathbf{x}_q$.

3) **Prediction (Majority Vote):** The predicted class label $\hat{y}_q$ for the query point is determined by a majority vote among its "k" nearest neighbors:

$$\hat{y}_q= arg\max_{c\in Y}\sum_{i\in N_k(\mathbf{x}_q)} I(y_i=c)$$

where Y is the set of possible class labels, and $I(\cdot)$ is the indicator function returning 1 if the argument is true and 0 otherwise.

4) **(Optional) Weighted Voting:** Neighbors can be weighted such that closer points have a greater influence on the vote. A common scheme is to use inverse distance weights: $w_i=1/d(\mathbf{x}_q,\mathbf{x}_i)$.

Key Process: The algorithm essentially defines a decision boundary based on local neighborhoods in the feature space. It is a lazy learner because all computation is deferred until the prediction phase.

8.3. Key Hyperparameters Optimized in This Study

In our implementation, the following core hyperparameters were tuned via grid search:

1) n_neighbors ("k"): The number of nearest neighbors to use for prediction. This is the most critical parameter, balancing bias and variance.
2) weights: The weighting function used in prediction. Options are uniform (all neighbors weigh equally) or distance (weight by the inverse of their distance).

3) p: The power parameter for the Minkowski distance metric (e.g., p=2 for uclidean, p=1 for Manhattan).

9. Light Gradient Boosting Machine (LightGBM)

9.1. Algorithm Overview

Light Gradient Boosting Machine (LightGBM) is a highly efficient, distributed gradient boosting framework developed by Microsoft(9). It is designed for speed, scalability, and lower memory usage while maintaining high predictive accuracy. LightGBM introduces two key innovations: Gradient-based One-Side Sampling (GOSS) and Exclusive Feature Bundling (EFB). GOSS retains instances with large gradients (which are harder to fit) and randomly samples instances with small gradients, focusing computational effort on informative samples. EFB bundles mutually exclusive sparse features into fewer dense features, effectively reducing the dimensionality. Combined with its core histogram-based algorithm and leaf-wise (best-first) tree growth strategy, LightGBM often outperforms other GBM implementations in terms of training speed and model performance, especially on large datasets.

9.2. Algorithm Formulation and Key Process

LightGBM builds upon the standard Gradient Boosting framework but optimizes it significantly. For a dataset $\{(\mathbf{x}_i, y_i)\}_{i=1}^{N}$, the objective at the m-th iteration to fit a new base learner $\mathrm{h}_m(\mathbf{x})$ (a decision tree) is:

$$\mathrm{L}^{(m)} \approx \sum_{i=1}^{N} \left[L(y_i, F_{m-1}(\mathbf{x}_i)) + g_i \mathrm{h}_m(\mathbf{x}_i) + \frac{1}{2} \mathrm{h}_i \mathrm{h}_m^2(\mathbf{x}_i) \right] + \Omega(\mathrm{h}_m)$$

where g_i and h_i are the first and second-order gradients of the loss function L with respect to $F_{m-1}(\mathbf{x}_i)$, and Ω is the regularization term on the tree's complexity (e.g., number of leaves, L2 regularization on leaf scores).

1) Histogram-based Learning: Instead of examining all possible split points for continuous features, LightGBM bins feature values into discrete histograms. The optimal split is found based on these histograms, drastically reducing computational cost.
2) Leaf-wise (Best-first) Tree Growth: Unlike level-wise growth that expands all leaves at the same depth, LightGBM grows the tree by splitting the leaf that

yields the largest gain in the loss function at each step. This often leads to deeper, more imbalanced, but significantly more accurate trees.

3) Gradient-based One-Side Sampling (GOSS): To select samples for training a new tree, GOSS keeps all instances with large gradients ($|g_i|) \geq \theta$ and randomly samples a fraction a of the remaining instances with small gradients. The sampled small-gradient instances are then weighted by a factor of $(1\text{-}a) / a$ to compensate for the changed data distribution.
4) Exclusive Feature Bundling (EFB): Many high-dimensional features are sparse and mutually exclusive (i.e., they rarely take non-zero values simultaneously). EFB identifies and bundles these features together without significant information loss, reducing the effective number of features.

9.3. Key Hyperparameters Optimized in This Study

In our implementation, the following core hyperparameters were tuned via grid search:

1) num_leaves: The maximum number of leaves in one tree. This is the primary parameter to control tree complexity in leaf-wise growth.
2) max_depth: An alternative constraint on tree depth to prevent overfitting.
3) learning_rate: The shrinkage factor applied to each tree's contribution.
4) n_estimators: The number of boosting iterations (trees).
5) min_data_in_leaf: The minimum number of samples in a leaf. A larger value helps prevent overfitting.
6) feature_fraction: The fraction of features randomly selected for building each tree (similar to max_features in RF).
7) bagging_fraction & bagging_freq: Parameters for stochastic gradient boosting, specifying the sample fraction and frequency for bagging.

10. Logistic Regression (LR)

Logistic Regression (LR) is a widely used statistical model for binary classification tasks(10). Despite its name, it is a linear model for classification rather than regression. LR models the probability that a given input belongs to a particular class (e.g., the positive class) by applying the logistic function (sigmoid) to a linear combination of the input features. The model is interpretable, efficient to train, and

often serves as a strong baseline. It can be extended to multi-class classification via the one-vs-rest or softmax approaches.

10.2. Algorithm Formulation and Key Process

Given a feature vector $x \in \mathrm{R}^D$, the logistic regression model computes the probability of the positive class ($y = 1$) as:

$$P(y=1 \mid \mathbf{x}) = \sigma(\mathbf{w}^T\mathbf{x}+b) = \frac{1}{1+\exp(-(\mathbf{w}^T\mathbf{x}+b))}$$

where $x \in \mathrm{R}^D$ is the weight vector, $b \in \mathrm{R}$ is the bias term, and $\sigma(\cdot)$ is the logistic (sigmoid) function that maps any real number to the range (0, 1).

The model parameters($\mathbf{w}$,b) are estimated by minimizing the negative log-likelihood, which for a dataset $\{(\mathbf{x}_i, y_i)\}_{i=1}^{N}$ with $y_i \in \{-1,+1\}$, leads to the **binary cross-entropy loss**:

$$J(\mathbf{w},b) = -\frac{1}{N}\sum_{i=1}^{N}[y_i \log(p_i)+(1-y_i)\log(1-p_i)]$$

where $p_i = P(y_i=1 \mid \mathbf{x}_i) = \sigma(\mathbf{w}^T\mathbf{x}_i+b)$.

The minimization is typically performed using gradient-based optimization algorithms such as gradient descent, stochastic gradient descent, or more advanced variants like L-BFGS. The gradients of the loss with respect to the parameters are:

$$\nabla_{\mathbf{w}} J = \frac{1}{N}\sum_{i=1}^{N}(p_i - y_i)\mathbf{x}_i$$

$$\frac{\partial J}{\partial b} = \frac{1}{N}\sum_{i=1}^{N}(p_i - y_i)$$

Regularization terms (L1 or L2) are often added to the loss function to prevent overfitting and improve generalization.

10.3. Key Hyperparameters Optimized in This Study

In our implementation, the following core hyperparameters were tuned via grid search:

1) C: The inverse of regularization strength (like$1/\lambda$). A smaller value specifies stronger regularization.
2) penalty: The norm used for regularization (e.g., L1 or L2).
3) solver: The optimization algorithm used to minimize the loss function (e.g., lbfgs, liblinear, sag, saga).

4) max_iter: The maximum number of iterations for the solver to converge.

11. Categorical Boosting (CatBoost)

11.1. Algorithm Overview

CatBoost, short for Categorical Boosting, is a high-performance, open-source gradient boosting library developed by Yandex(11). It is specifically designed to handle categorical features natively and efficiently, without the need for extensive preprocessing like one-hot encoding, which can lead to a high-dimensional and sparse feature space. Its two most significant innovations are the use of Ordered Boosting and advanced methods for processing categorical features using Ordered Target Statistics. These innovations effectively reduce overfitting and target leakage (a common pitfall when applying standard methods to categorical data), making CatBoost robust, accurate, and efficient for a wide range of tasks.

11.2. Algorithm Formulation and Key Process

CatBoost builds upon the gradient boosting framework but modifies it substantially to handle ordered data (e.g., time series or randomly permuted rows) and categorical features.

1) **Prediction Function:** Like other GBM variants, it builds an ensemble of Mdecision trees in an additive manner:

$$F_M(\mathbf{x})=F_0(\mathbf{x})+v\sum_{m=1}^{M}\mathrm{h}_m(\mathbf{x})$$

where F_0 is the initial prediction, h_m is the m-th tree, and v is the learning rate.

2) **Ordered Boosting:** To prevent target leakage—where information from the sample being predicted is inadvertently used during its own training—CatBoost employs a permutation-driven approach. A random permutation σ of the training examples is generated. When training the m-th tree, the model uses only the first i samples in the permutation σ to calculate the gradient for the i-th sample. This creates a chain of dependencies that simulates a leave-one-out style validation for each sample, making the gradient estimates unbiased and reducing overfitting.

3) **Processing Categorical Features:** Instead of one-hot encoding, CatBoost transforms a categorical feature into numerical values using **Ordered Target**

Statistics. For a given categorical feature value, its numerical representation is calculated based on the target values of *only those training samples that have appeared before the current sample* in the permutation σ. A common formula involves a combination of prior probability and the observed frequency:

$$TS=\frac{\sum_{j=1}^{p\text{-}1}[x_{\sigma_j,k}=x_{\sigma_p,k}]\cdot\ y_{\sigma_j}+a\cdot\ P}{\sum_{j=1}^{p\text{-}1}[x_{\sigma_j,k}=x_{\sigma_p,k}]+a}$$

where $x_{\sigma_j,k}$ is the categorical value for the *p-t*h sample in permutation σ for feature k, σ is a prior weight (smoothing parameter), and P is a prior value (e.g., the average target in the dataset). This method provides a regularized, order-aware encoding.

4) **Symmetric Tree Structure:** CatBoost uses oblivious trees (or symmetric trees), where all nodes at the same depth use the same feature and splitting condition. This structure speeds up prediction and reduces overfitting.

11.3. Key Hyperparameters Optimized in This Study

In our implementation, the following core hyperparameters were tuned via grid search:

1) iterations: The number of boosting trees to build (M).
2) learning_rate (v): The step size shrinkage used to prevent overfitting.
3) depth: The maximum depth of the symmetric trees.
4) l2_leaf_reg: The coefficient for L2 regularization of the leaf values.
5) cat_features: The specification of which features are categorical (handled automatically from the dataset).
6) random_strength: The amount of randomness to use in scoring splits to prevent overfitting.

12. eXtreme Gradient Boosting (XGBoost)

12.1. Algorithm Overview

eXtreme Gradient Boosting (XGBoost) is a highly optimized, scalable, and widely adopted implementation of gradient boosting machines(12). It has become a benchmark algorithm in machine learning competitions and practical applications due to its exceptional performance, speed, and robustness. XGBoost enhances the standard gradient boosting framework by incorporating regularization terms in its

objective function to control model complexity and prevent overfitting. It also utilizes a second-order Taylor expansion to approximate the loss function, which allows for more precise optimization. Additional innovations include efficient handling of missing values, block-based parallelization for tree construction, and a novel sparsity-aware algorithm for sparse data.

12.2. Algorithm Formulation and Key Process

Given a dataset with N samples and D features, XGBoost builds an ensemble of K additive decision trees:

$$\hat{y}_i = \phi(\mathbf{x}_i) = \sum_{k=1}^{K} f_k(\mathbf{x}_i), \quad f_k \in \mathrm{F}$$

where F is the space of regression trees (CART). The model is trained by minimizing the following **regularized objective function**:

$$\mathrm{L}(\phi) = \sum_{i=1}^{N} l(y_i, \hat{y}_i) + \sum_{k=1}^{K} \Omega(f_k)$$

The first term l is a differentiable convex loss function (e.g., logistic loss for classification). The second term Ω penalizes model complexity:

$$\Omega(f) = \gamma T + \frac{1}{2}\lambda \|\mathbf{w}\|^2$$

Here, T is the number of leaves in the tree, **w** is the vector of leaf scores (weights), γ is a complexity penalty for adding a new leaf, and λ is the L2 regularization term on the leaf weights.

1) **Approximate Training (Second-Order Optimization):** XGBoost does not use only the first-order gradient. At the t-th iteration, the objective is approximated via a second-order Taylor expansion around the prediction from the previous iteration $\hat{y}^{(t-1)}$:

$$\mathrm{L}^{(t)} \approx \sum_{i=1}^{N} \left[g_i f_t(\mathbf{x}_i) + \frac{1}{2} \mathrm{h}_i f_t^2(\mathbf{x}_i) \right] + \Omega(f_t)$$

where $g_i = \partial_{\hat{y}^{(t-1)}} l(y_i, \hat{y}^{(t-1)})$ *and* $\mathrm{h}_i = \partial^2_{\hat{y}^{(t-1)}} l(y_i, \hat{y}^{(t-1)})$ are the first and second-order gradients (gradient and Hessian) of the loss function.

2) **Optimal Leaf Weight and Split Finding:** For a fixed tree structure q that maps samples to leaves, let $I_j = \{i \mid q(\mathbf{x}_i) = j\}$ be the set of samples in leaf j. The optimal weight w_j^* for that leaf and the corresponding optimal objective value are:

$$w_j^*=-\frac{\sum_{i\in I_j} g_i}{\sum_{i\in I_j} \mathrm{h}_i+\lambda},\quad \text{and } \tilde{\mathrm{L}}^{(t)}(q)=-\frac{1}{2}\sum_{j=1}^{T}\frac{(\sum_{i\in I_j} g_i)^2}{\sum_{i\in I_j} \mathrm{h}_i+\lambda}+\gamma T$$

The **split gain** formula, used to evaluate the quality of a candidate split that partitions a parent node I into left and right children I_L and I_R, is:

$$G_{split}=\frac{1}{2}\left[\frac{(\sum_{i\in I_L} g_i)^2}{\sum_{i\in I_L} \mathrm{h}_i+\lambda}+\frac{(\sum_{i\in I_R} g_i)^2}{\sum_{i\in I_R} \mathrm{h}_i+\lambda}-\frac{(\sum_{i\in I} g_i)^2}{\sum_{i\in I} \mathrm{h}_i+\lambda}\right]-\gamma$$

The algorithm searches for the split that maximizes this gain. A negative gain indicates the split is not beneficial, and the node becomes a leaf.

12.3. Key Hyperparameters Optimized in This Study

In our implementation, the following core hyperparameters were tuned via grid search:

1) n_estimators / num_boost_round: The number of boosting rounds (trees, K).
2) learning_rate (η): Step size shrinkage, applied to the leaf weights (w_j^*) after calculation.
3) max_depth: Maximum depth of a tree. Controls model complexity.
4) min_child_weight: Minimum sum of instance weight (Hessian, h_i) needed in a child node. A larger value makes the algorithm more conservative.
5) gamma (γ): Minimum loss reduction required to make a further partition on a leaf node.
6) subsample: Subsample ratio of the training instances for each tree (stochastic boosting).
7) colsample_bytree: Subsample ratio of columns (features) for each tree.
8) reg_lambda (λ): L2 regularization term on leaf weights.

Supplementary Material 2 References

1. Breiman L. Random Forests. Machine Learning. 2001;45(1):5-32.

2. Cortes C, Vapnik V. Support-vector networks. Machine Learning. 1995;20(3):273-97.

3. Friedman JH. Greedy function approximation: A gradient boosting machine. The Annals of Statistics. 2001;29(5):1189-232, 44.

4. Freund Y, Schapire RE. A Decision-Theoretic Generalization of On-Line

Learning and an Application to Boosting. Journal of Computer and System Sciences. 1997;55(1):119-39.

5. Rumelhart DE, Hinton GE, Williams RJ. Learning representations by back-propagating errors. Nature. 1986;323(6088):533-6.

6. Quinlan JR, Morris G, Jackson J, O'Connell P. C4.5 : programs for machine learning. San Mateo, Calif: Morgan Kaufmann Publishers; 1993.

7. Geurts P, Ernst D, Wehenkel L. Extremely randomized trees. Machine Learning. 2006;63(1):3-42.

8. Cover T, Hart P. Nearest neighbor pattern classification. IEEE Transactions on Information Theory. 1967;13(1):21-7.

9. Ke G, Meng Q, Finley T, Wang T, Chen W, Ma W, et al., editors. LightGBM: A Highly Efficient Gradient Boosting Decision Tree. Advances in Neural Information Processing Systems; 2017: Curran Associates, Inc.

10. Hosmer DW, Lemeshow S, Sturdivant RX, ProQuest. Applied logistic regression. Third edition ed. Hoboken, N.J: Wiley; 2013.

11. Prokhorenkova L, Gusev G, Vorobev A, Dorogush AV, Gulin A. CatBoost: unbiased boosting with categorical features. 2019.

12. Chen T, Guestrin C, editors. XGBoost: A Scalable Tree Boosting System. Proceedings of the 22nd ACM SIGKDD International Conference on Knowledge Discovery and Data Mining; 2016/august: ACM.
