# Supplementary figures and images for: Machine learning models for predicting extended length of stay and hospital charges in nontraumatic subarachnoid hemorrhage
Source: Front Neurol. 2026 Feb 4;17:1737503. doi: 10.3389/fneur.2026.1737503 (PMC12913072; doi:10.3389/fneur.2026.1737503)

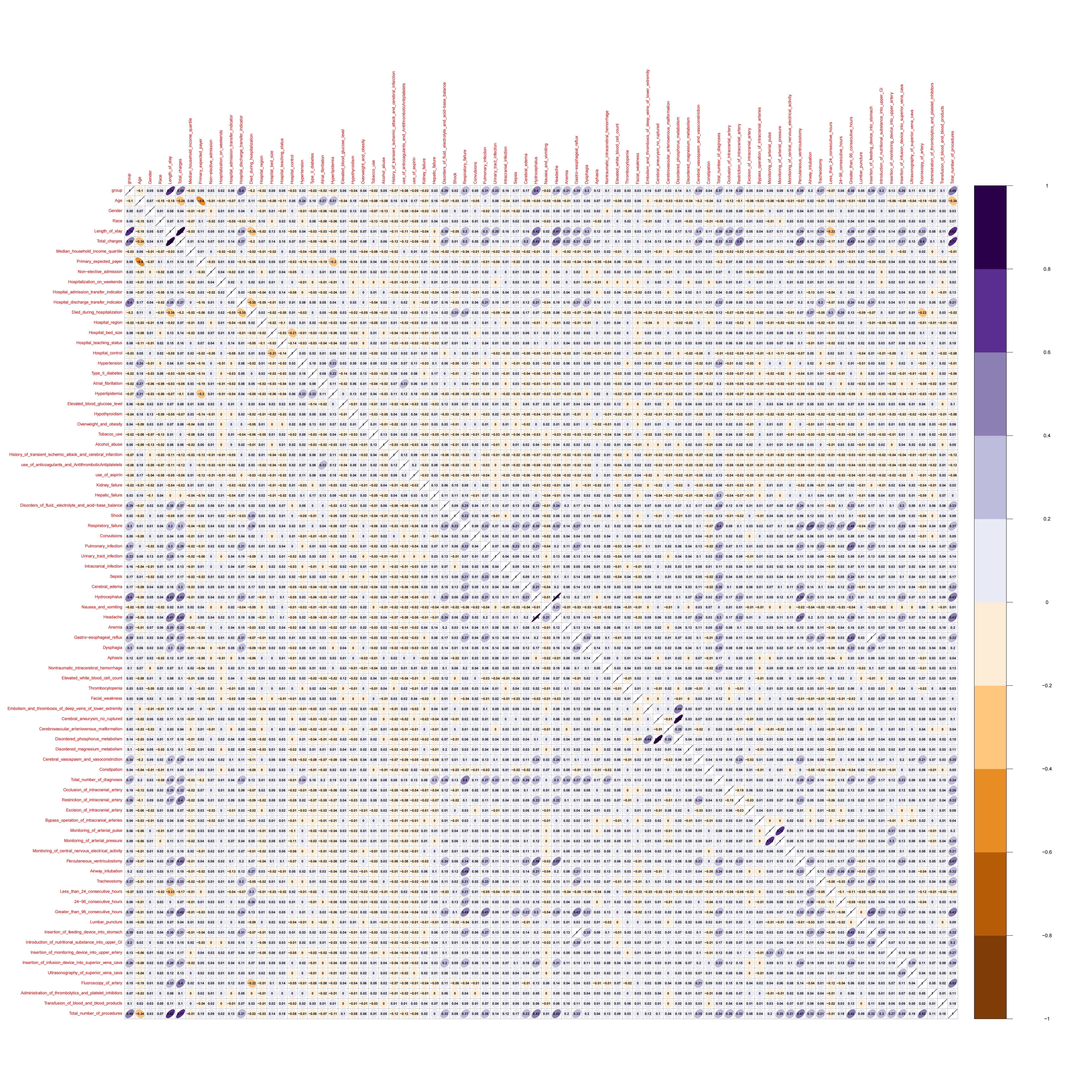

Supplement: Supplementary file 11 [file Image_1.jpeg]

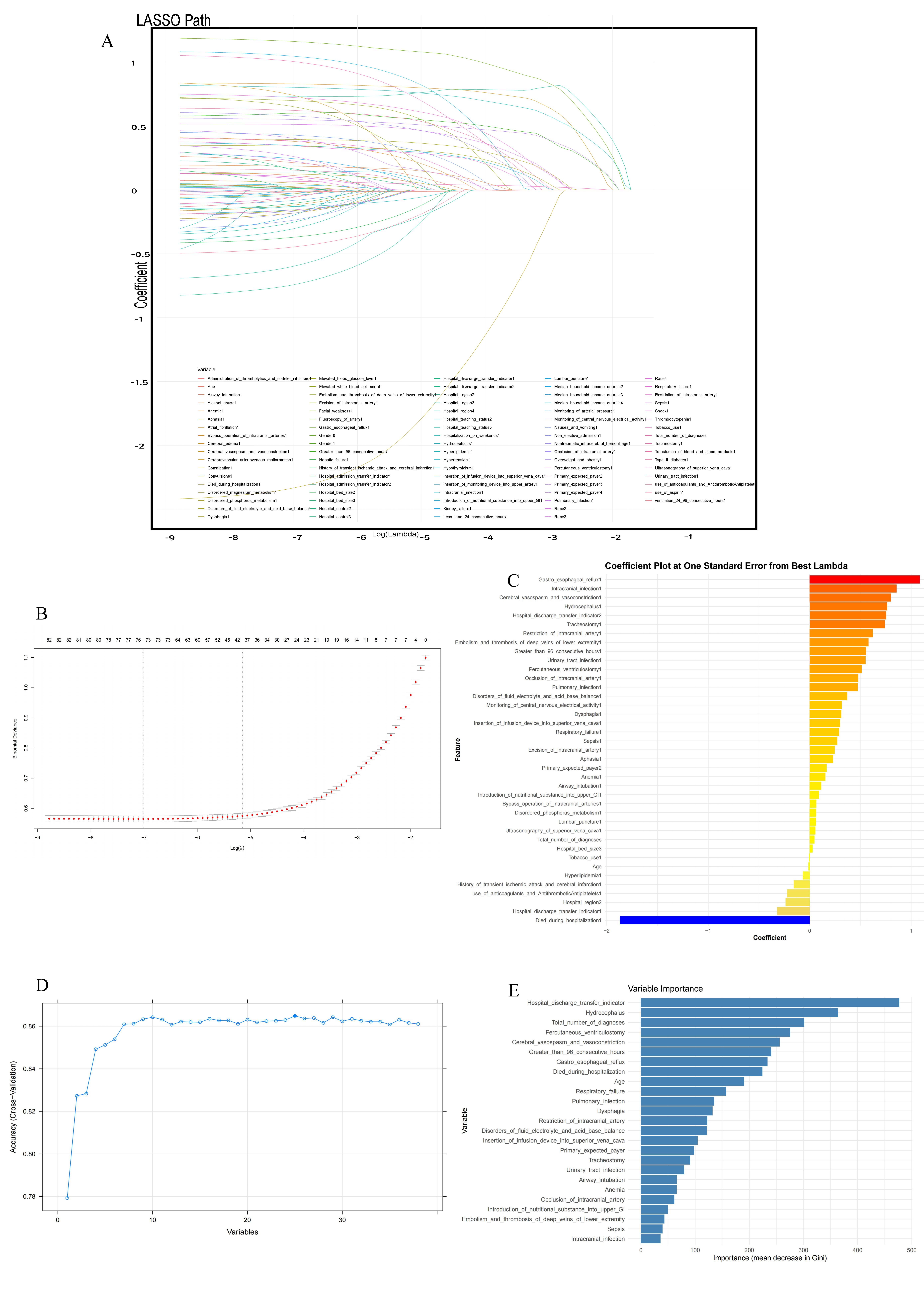

Supplement: Supplementary file 12 [file Image_2.jpeg]

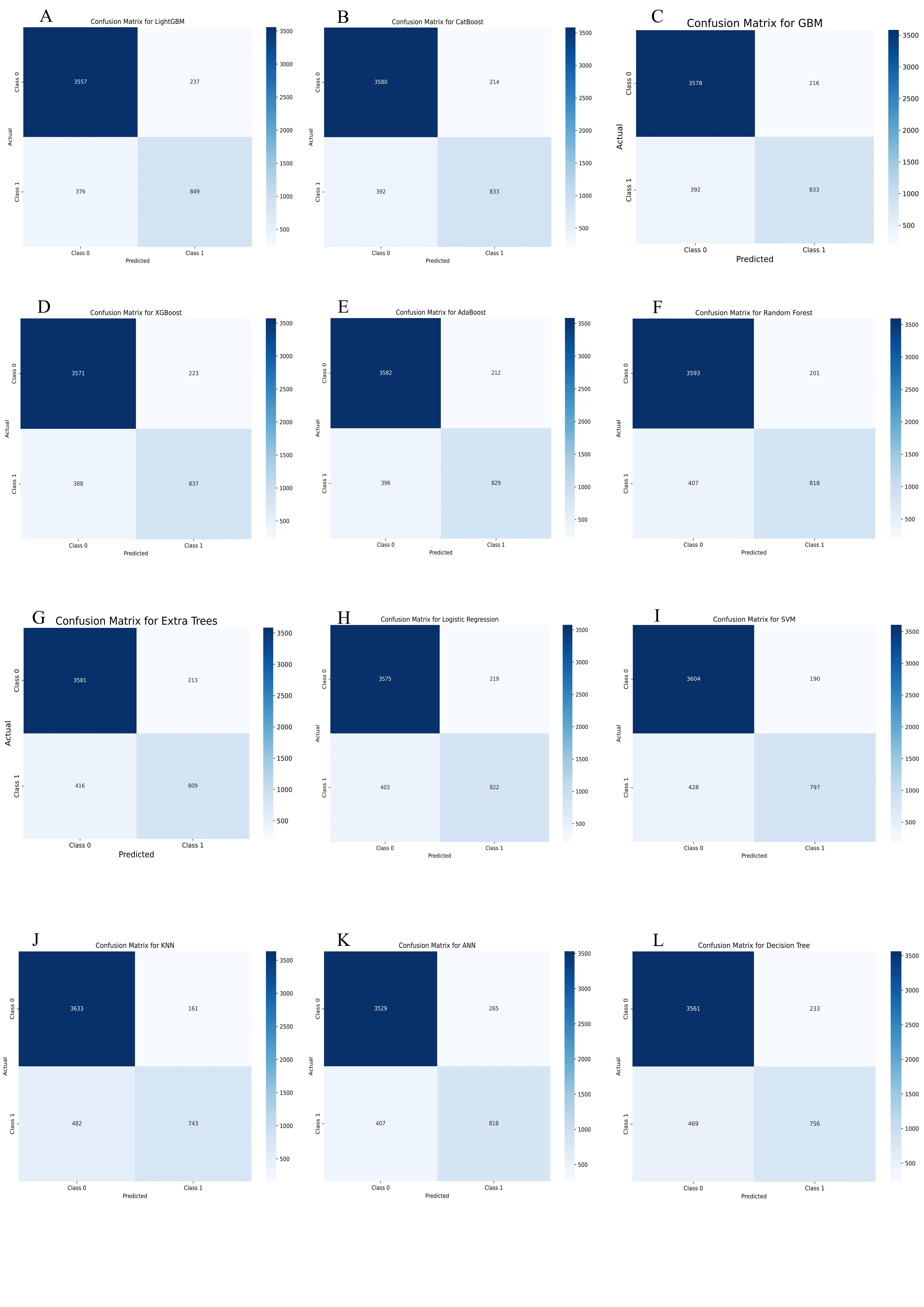

Supplement: Supplementary file 13 [file Image_3.jpeg]

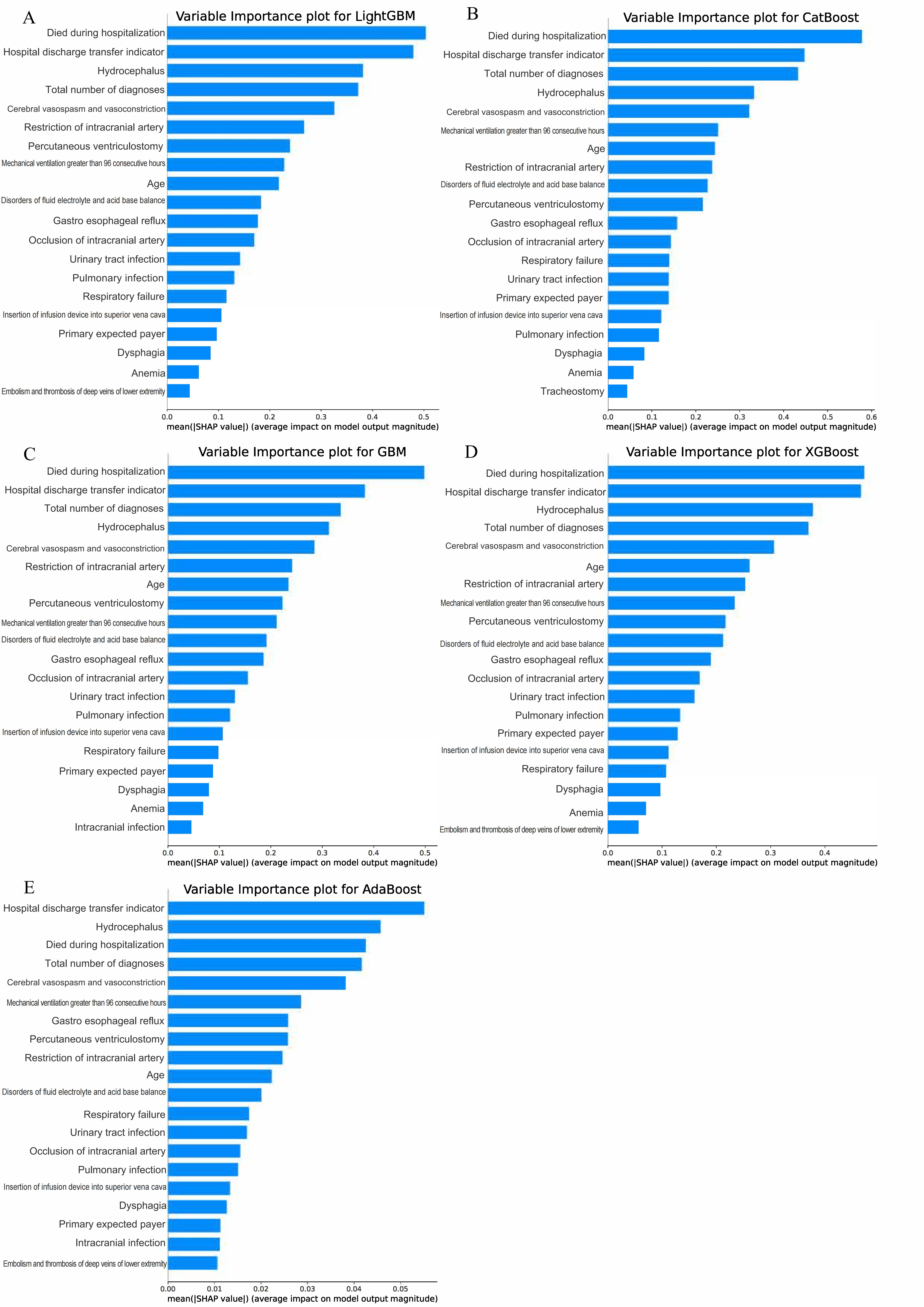

Supplement: Supplementary file 14 [file Image_4.jpeg]

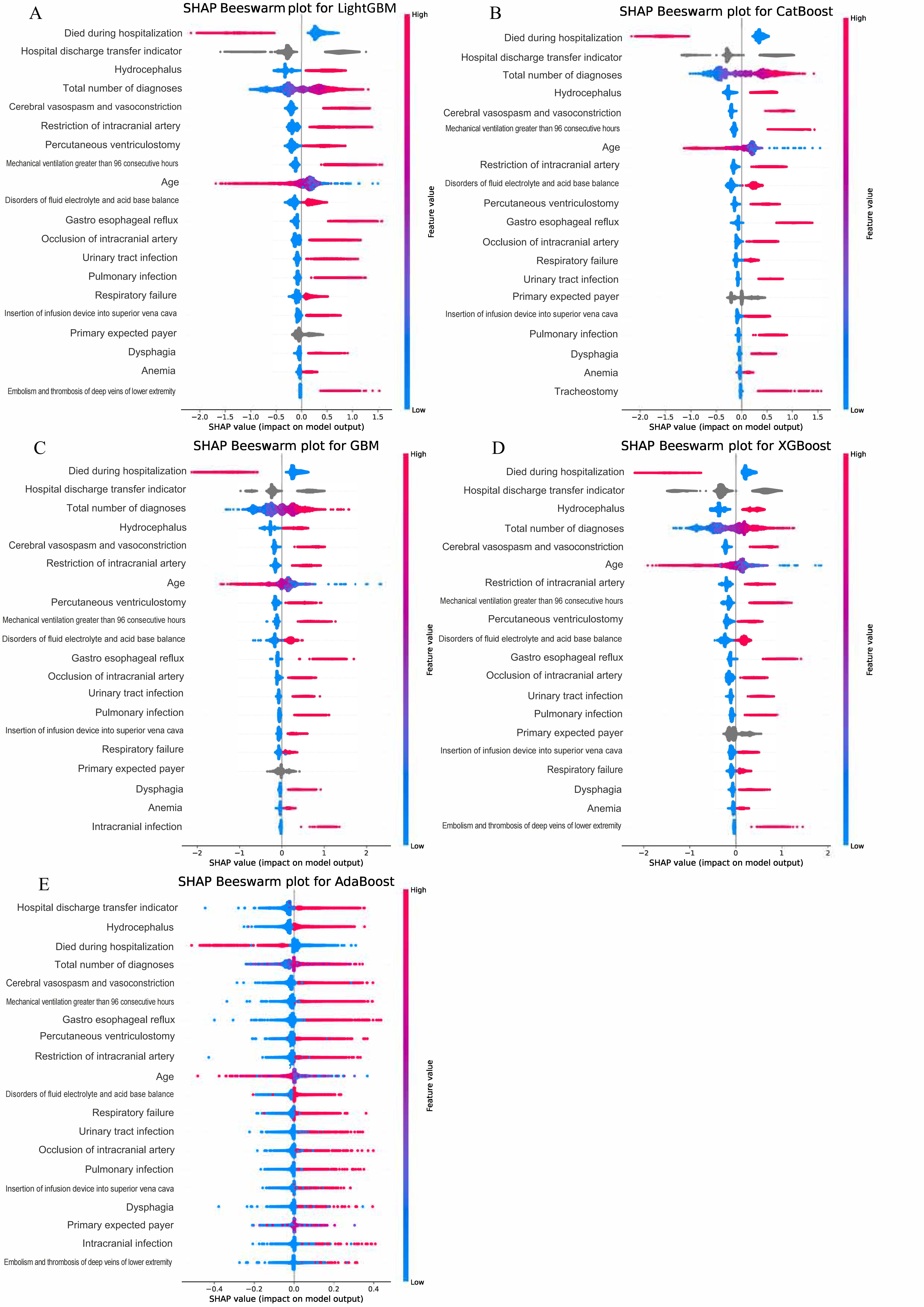

Supplement: Supplementary file 15 [file Image_5.jpeg]

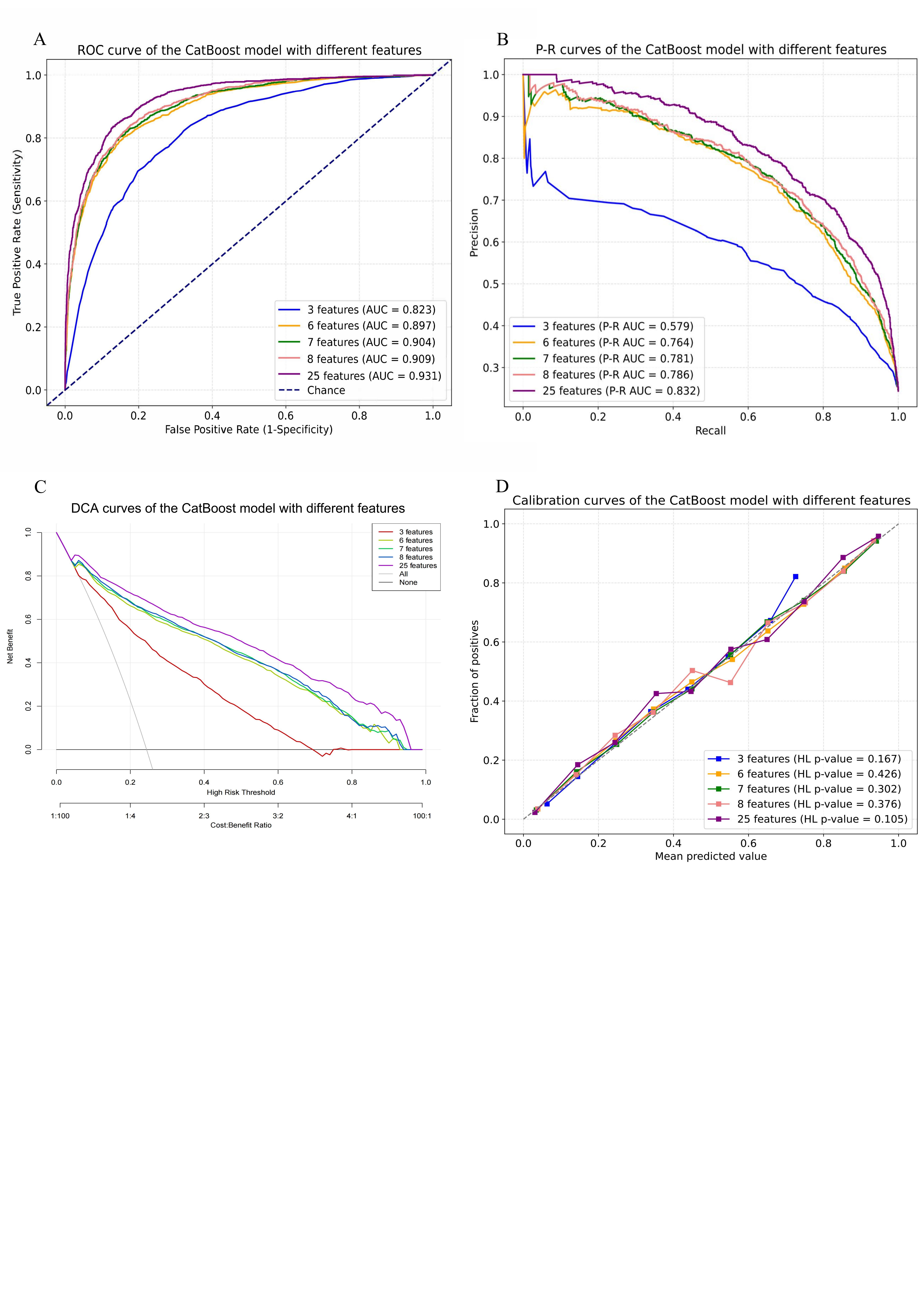

Supplement: Supplementary file 16 [file Image_6.jpeg]

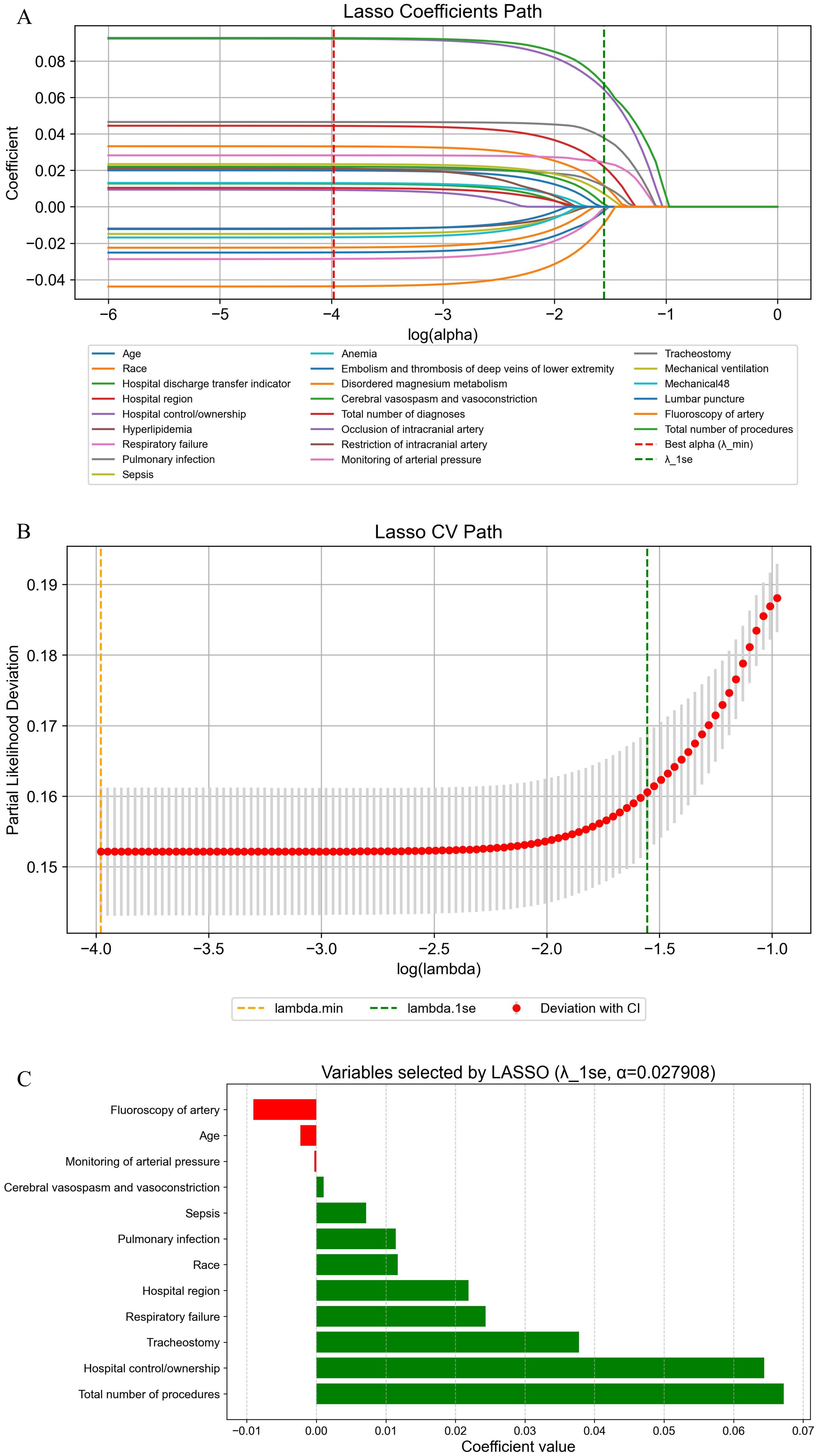

Supplement: Supplementary file 17 [file Image_7.jpeg]
